# An Algorithm to Automatically Generate the Combinatorial Orbit Counting Equations

**DOI:** 10.1371/journal.pone.0147078

**Published:** 2016-01-21

**Authors:** Ine Melckenbeeck, Pieter Audenaert, Tom Michoel, Didier Colle, Mario Pickavet

**Affiliations:** 1 Department of Information Technology (INTEC), Ghent University-iMinds, Ghent, Belgium; 2 The Roslin Institute, University of Edinburgh, Easter Bush, Midlothian, Scotland, United Kingdom; Nankai University, CHINA

## Abstract

Graphlets are small subgraphs, usually containing up to five vertices, that can be found in a larger graph. Identification of the graphlets that a vertex in an explored graph touches can provide useful information about the local structure of the graph around that vertex. Actually finding all graphlets in a large graph can be time-consuming, however. As the graphlets grow in size, more different graphlets emerge and the time needed to find each graphlet also scales up. If it is not needed to find each instance of each graphlet, but knowing the number of graphlets touching each node of the graph suffices, the problem is less hard. Previous research shows a way to simplify counting the graphlets: instead of looking for the graphlets needed, smaller graphlets are searched, as well as the number of common neighbors of vertices. Solving a system of equations then gives the number of times a vertex is part of each graphlet of the desired size. However, until now, equations only exist to count graphlets with 4 or 5 nodes. In this paper, two new techniques are presented. The first allows to generate the equations needed in an automatic way. This eliminates the tedious work needed to do so manually each time an extra node is added to the graphlets. The technique is independent on the number of nodes in the graphlets and can thus be used to count larger graphlets than previously possible. The second technique gives all graphlets a unique ordering which is easily extended to name graphlets of any size. Both techniques were used to generate equations to count graphlets with 4, 5 and 6 vertices, which extends all previous results. Code can be found at https://github.com/IneMelckenbeeck/equation-generator and https://github.com/IneMelckenbeeck/graphlet-naming.

## Introduction

A multitude of domains use graphs as a modeling tool. Obvious uses include the modeling of networks, such as social, communications and transport networks. Plenty of metrics exist to characterize them, for instance lengths of shortest paths or size of clusters of vertices. In recent years, though, graphlets are gaining more popularity as a method of characterizing graphs.

The following symbols will be used throughout this paper for clarity. A graph *G* consists of a set of vertices, called *V*, and a set of edges *E*, so that each edge connects two vertices. As such, an edge can be notated by listing the couple of vertices it connects:

$$\begin{array}{c} e=\{x,y\}:x,y\in V(G). \end{array}$$ (1)

The graph is notated as

$$\begin{array}{c} G=(V,E). \end{array}$$ (2)

The number of vertices in a graph is called its *order*, the number of edges its *size*. Isomorphisms between two graphs are bijections which map one graph’s vertices to the other’s so that both edge sets are the same. The set of isomorphisms between two graphs *G* and *H* is given by

$$\begin{array}{c} Iso(G,H)=\{f:V(G)\to V(H)|\{u,v\}\in E(G)↔\{f(u),f(v)\}\in E(H)\}. \end{array}$$ (3)

Graphs are isomorphic if they have at least one isomorphism: *G* ≃ *H* ⇔ *Iso*(*G*, *H*)≠∅. Isomorphisms from a graph to itself are called automorphisms:

$$\begin{array}{c} Aut(G)=Iso(G,G). \end{array}$$ (4)

**Fig 1:**
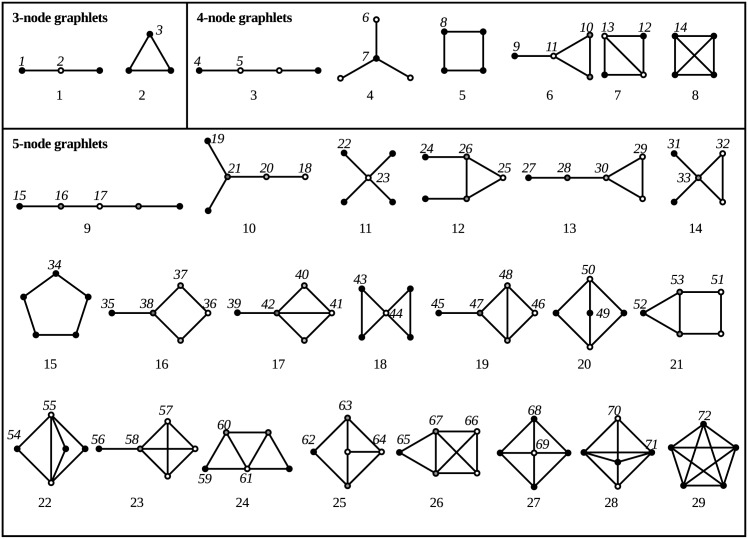
All graphlets up to order 5. The numbers in normal font are Pržulj’s graphlet ordering. Within each graphlet, the vertices with equal color are in the same orbit. The numbers in italic font are Pržulj’s orbit ordering.

Pržulj, Corneil and Jurisica define graphlets in [1] as connected graphs with a small number of vertices. All of these graphlets containing up to order 5 can be seen in Fig 1. The x^th^ graphlet in this numbering will be called *graphlet x*, all graphlets of order *n* are called *n-graphlets*. An induced subgraph of a graph is defined as a subgraph containing a collection of vertices and all edges between those vertices. A graph *H* is an induced subgraph of graph *G* = (*V*, *E*) if

$$\begin{array}{c} H=(V^{\prime }\subseteq V,\left\{\left\{a,b\right\}\in E|a,b\in V^{\prime }\right\}) \end{array}$$ (5)

As graphlets are themselves graphs, a graph in which graphlets are searched will further be called the *explored graph*. Specific induced subgraphs can be searched within an explored graph, either listing each occurrence of the subgraph or simply counting them. When these induced subgraphs of the explored graph are isomorphic to a graphlet, all vertices in such an induced subgraph are said to touch that graphlet. The induced subgraph itself is then also said to be an instance of that graphlet.

The vertices of a graphlet can be subdivided into different orbits [2], which are sets of vertices which are mapped onto each other by the graphlet’s automorphisms. The orbit of a vertex *x* is given by

$$\begin{array}{c} Orb(x)=\{y\in V(G)|\exists g\in Aut(G):y=g(x)\}. \end{array}$$ (6)

In Fig 1, vertices in the same orbit have the same color. Przulj [2] ordered the orbits and gave each of them a number for easy identification. These numbers can also be seen in Fig 1. Similarly to graphlets, the orbit with number n will be called *orbit n* and vertices of an explored graph touching a graphlet are also said to touch the orbit. The graphlet degree distribution (GDD) for a specific orbit *o* and a certain number *k* is defined as the number of vertices in the explored graph that touch orbit *o* exactly *k* times [2]. These GDDs are used to obtain information about the local structure in graphs.

**Fig 2:**
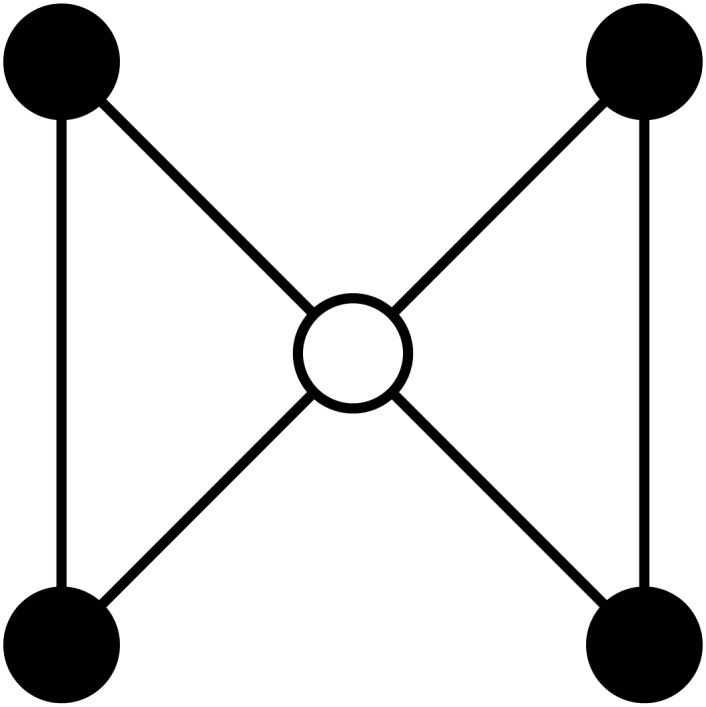
Graphlet 18. The black outer vertices form orbit 43, the white inner vertex is orbit 44.

An example of a graphlet can be seen in Fig 2. This graphlet will be used to demonstrate different concepts in this article. In Fig 1, it can be seen that this graphlet is graphlet 18. No other vertex is symmetric to the middle one, which alone forms orbit 44. The left pair of black vertices can be swapped without the structure of the graphlet changing, as can the right pair. Both pairs can likewise be interchanged without structural change. Therefore, all of these vertices belong to the same orbit, which is called orbit 43.

The more vertices are allowed in a graphlet, the more different graphlets there are. Within a simple graph with n vertices, there are (n2) possible edges, which can independently be present or absent. The number of possible graphs on n vertices is therefore O(2(n2)). This is a loose upper bound for the number of possible graphlets, because not all of these graphs will be connected. Many of these graphlets will be isomorphic to each other, further reducing the number of actual graphlets.

**Fig 3:**
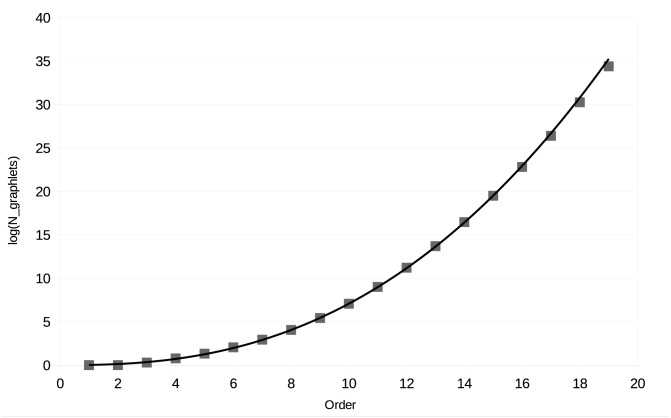
The number of graphlets for each order. The logarithm of the number of graphlets is plotted against the number of vertices in each graphlet. The curve is an exponential fit: *f*(*n*) = 0.022*n*^2.50^, which has a coefficient of determination *R*^2^ = 0.9989.

The actual number of graphlets containing up to 19 vertices can be found at the Online Encyclopedia of Integer Sequences [3] and is shown in Table 1. Fig 3 shows the logarithm of the number of graphlets in function of the number of vertices. These are fitted by a power function. Its coefficient of determination is 0.9989, meaning it is a good fit for the number of graphlets of order less than 20. The number of graphlets of a certain order grows exponentially with growing order, therefore no algorithms running over all possible graphlets of a certain order can have a complexity that is smaller than exponential in the number of vertices in a graphlet.

**Table 1:** The number of graphlets with n nodes.

Order	Number of graphlets
1	1
2	1
3	2
4	6
5	21
6	112
7	853
8	11117
9	261080
10	11716571

**Fig 4:**
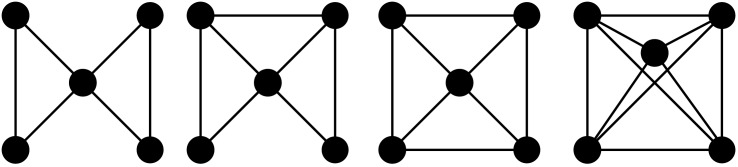
A motif isomorphic to graphlet 18. All other graphs contain instances of this motif, but would not if it were a graphlet. This is not a complete list of possible instances of this motif.

Motifs are a concept related to, but distinct from graphlets. Motifs are any connected subgraphs of a larger graph, not only *induced* subgraphs, that occur statistically significantly more in the explored graph, compared to what would be expected in a random graph of equal size and order. The difference between motifs and graphlets is shown in Fig 4: if the first graph is a motif, all of the other graphs are different instances of it; if it is a graphlet, none of the others are an instance of it. A more formal definition: graph *G* is an instance of motif *M* if and only if

$$\begin{array}{c} \exists f:V(M)\to V(G)|\{u,v\}\in E(M)\Rightarrow \{f(u),f(v)\}\in E(G). \end{array}$$ (7)

Since only certain significant subgraphs are required, not all motifs are counted. ISMAGS [4, 5] is one motif finding algorithm which lists each occurrence of a specific motif within an explored graph quickly. As there is no distinction between which graphlets are ‘significant’ and which not, counting all graphlets at the same time can be more useful than counting each of them apart.

ORCA [6] uses combinatorial calculations to simplify computing the number of times each vertex of the explored graph touches each orbit. It counts the number of times each vertex touches each orbit without actually finding the corresponding graphlets. It calculates this for all orbits of a certain order at the same time. Exploring the different ways a new vertex can be added to a graphlet, a system of equations was composed that reduces counting graphlets to finding graphlets of smaller order and finding common neighbors of vertices. This way, if one wants to know all GDDs of order n, it suffices to find all graphlets of order n-1 and solve the system of equations for each vertex. This greatly improves the time needed to find those graphlets.

ORCA, however, has the shortcoming that is is not easily scaleable. Generally, connected networks with up to 5 vertices are considered graphlets. This is due to the fact that the number of different graphlets increases quickly with each added vertex, though there is no hard limit on the number of vertices in a graphlet. ORCA’s equations were composed by hand, meaning that anyone wanting to use graphlets of a larger order would need to compose a new system of equations from scratch.

In this paper, a procedure to automatically generate ORCA’s equations for any order is presented. Step by step, the theory that will allow the automatic counting of graphlets will be built. In order to count the graphlets, an expandable naming system for graphlets is introduced. This naming system copies some features from Pržulj’s commonly used naming system, but it is more adapted to automatic expansion to graphlets of larger order.

## Methods

### Representing graphs, graphlets and orbits

Graphs are collections of vertices and edges, where each edge must connect exactly two vertices, but each vertex can connect to any number of edges. As such, graphs can be represented just like that: a set of vertices and a set of edges. Within a graphlet, there is no real reason to name the vertices anything in particular. It suffices to save the number of vertices (*n*), thereby implying the vertices are called 0, 1, …, n-1. As each edge connects two vertices, it can be represented by listing the indices of two vertices. One possible set of edges corresponding to graphlet 18 is:

$$\begin{array}{c} \{\{0,1\},\{0,2\},\{0,3\},\{0,4\},\{1,4\},\{2,3\}\}. \end{array}$$ (8)

**Fig 5:**
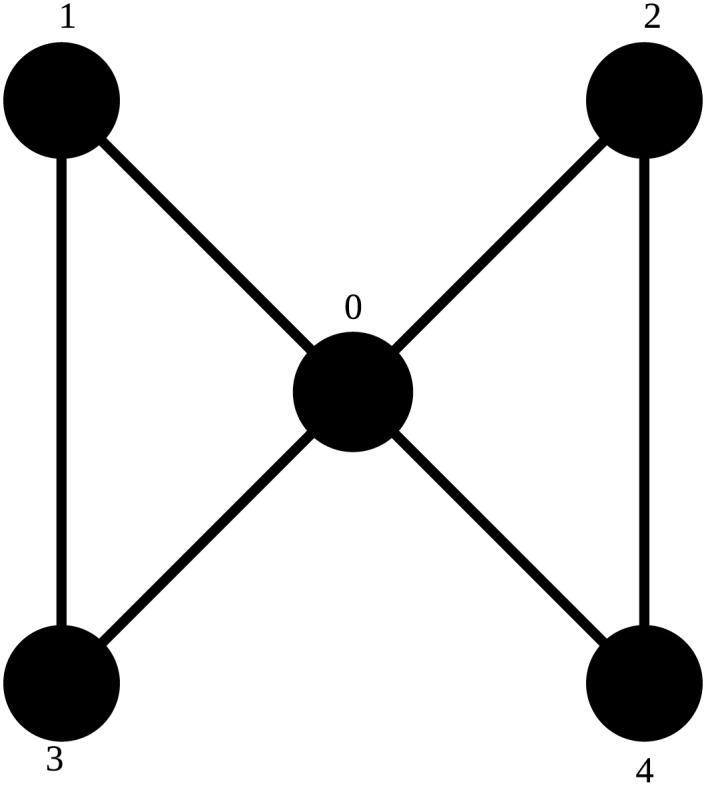
Graphlet 18 with numbered vertices. The graphlet has all edges in Eq (8).

This graphlet can be seen in Fig 5.

As *G* ≃ *H* ⇔ *Iso*(*G*, *H*)≠∅, when checking isomorphisms it is necessary to check whether any permutation of the vertices of one graphlet changes its edge set into the other’s edge set. Both graphlets’ vertices are named identically, so it is actually needed to check whether any of one graphlet’s automorphisms creates an edge set which is identical to the other’s. To avoid recalculation, all different sets of edges obtained by these automorphisms are also saved in the graphlet object. All possible edge sets for graphlet 18 that are generated are shown in Table 2.

**Table 2:** All edge sets that are isomorphic to graphlet 18.

{{0,1}, {0,2}, {0,3}, {0,4}, {1,2}, {3,4}}
{{0,1}, {0,2}, {0,3}, {0,4}, {1,3}, {2,4}}
{{0,1}, {0,2}, {0,3}, {0,4}, {1,4}, {2,3}}
{{0,1}, {0,2}, {1,2}, {1,3}, {1,4}, {3,4}}
{{0,1}, {0,3}, {1,2}, {1,3}, {1,4}, {2,4}}
{{0,1}, {0,4}, {1,2}, {1,3}, {1,4}, {2,3}}
{{0,1}, {0,2}, {1,2}, {2,3}, {2,4}, {3,4}}
{{0,2}, {0,3}, {1,2}, {1,4}, {2,3}, {2,4}}
{{0,2}, {0,4}, {1,2}, {1,3}, {2,3}, {2,4}}
{{0,1}, {0,3}, {1,3}, {2,3}, {2,4}, {3,4}}
{{0,2}, {0,3}, {1,3}, {1,4}, {2,3}, {3,4}}
{{0,3}, {0,4}, {1,2}, {1,3}, {2,3}, {3,4}}
{{0,1}, {0,4}, {1,4}, {2,3}, {2,4}, {3,4}}
{{0,2}, {0,4}, {1,3}, {1,4}, {2,4}, {3,4}}
{{0,3}, {0,4}, {1,2}, {1,4}, {2,4}, {3,4}}

When permuting the vertices, the orbits of the graphlet can also be calculated. When interchanging some vertices does not change the edge set of a graphlet, an automorphism is found. All vertices that were changed must then belong to the same orbit.

#### Orbit representatives

Using a good way to represent and identify orbits can simplify the problem greatly. An orbit is a set of vertices from a graphlet which can be mapped onto each other by an automorphism of the graphlet. As an orbit is meaningless without its graphlet, an orbit can be represented as a graphlet with one marked vertex, which will be excluded whenever the vertices of the graphlet are permuted. This graphlet will then be called an orbit representative, as the marked vertex will be a representative for all vertices in the same orbit. An orbit representative will be noted as *G*(*x*), with *x* ∈ *V* the marked vertex. For ease of use, the marked vertex is called vertex 0. The formula for the set of isomorphisms between two orbit representatives then becomes:

$$\begin{array}{ccc} Iso(G(x),H(y)) & = & \{f:V(G(x))\to V(H(y))|\{u,v\}\in E(G)↔\\ & & \{f(u),f(v)\}\in E(H)\wedge f(x)=y\}. \end{array}$$ (9)

**Fig 6:**
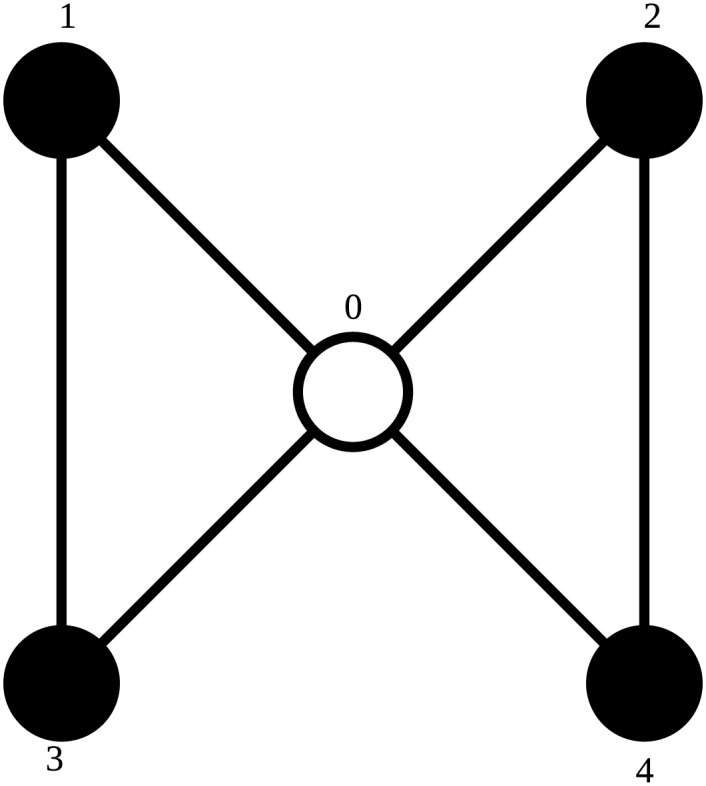
Orbit representative 44. The central vertex is marked and called vertex 0, to indicate that it cannot be interchanged with any other vertex.

Indeed, this way two orbit representatives are equal if and only if the list of edges is equal under some permutation of their vertices, excluding vertex 0. When talking about an orbit representative, that representative will get the same name as the orbit it represents. For example, a representative for orbit 44 will be called orbit representative 44. An illustration of this orbit representative can be seen in Fig 6. The isomorphic edge sets can be seen in Table 3.

**Table 3:** All edge sets that are isomorphic to orbit representative 44.

{{0,1}, {0,2}, {0,3}, {0,4}, {1,2}, {3,4}}
{{0,1}, {0,2}, {0,3}, {0,4}, {1,3}, {2,4}}
{{0,1}, {0,2}, {0,3}, {0,4}, {1,4}, {2,3}}

The other action permutations are used for, the calculation of orbits, can also be used within the orbit representatives. The definition of automorphisms and orbits are analogous to before,

$$\begin{array}{c} Aut(G(n))=Iso(G(n),G(n)) \end{array}$$ (10)

$$\begin{array}{c} Orb(x)=\{y\in V(G(n))|\exists g\in Aut(G(n)):y=g(x)\} \end{array}$$ (11)

but the changed definition of an isomorphism also changes the outcome. The orbits found in this way are different from the orbits found when all vertices are included in the permutations. As the orbits will be subdivided into smaller orbits, these groups will be called *suborbits*. These suborbits will come in handy when composing the equations, as they contain groups of vertices within the orbit representative that are equivalent as seen from the marked node.

**Fig 7:**
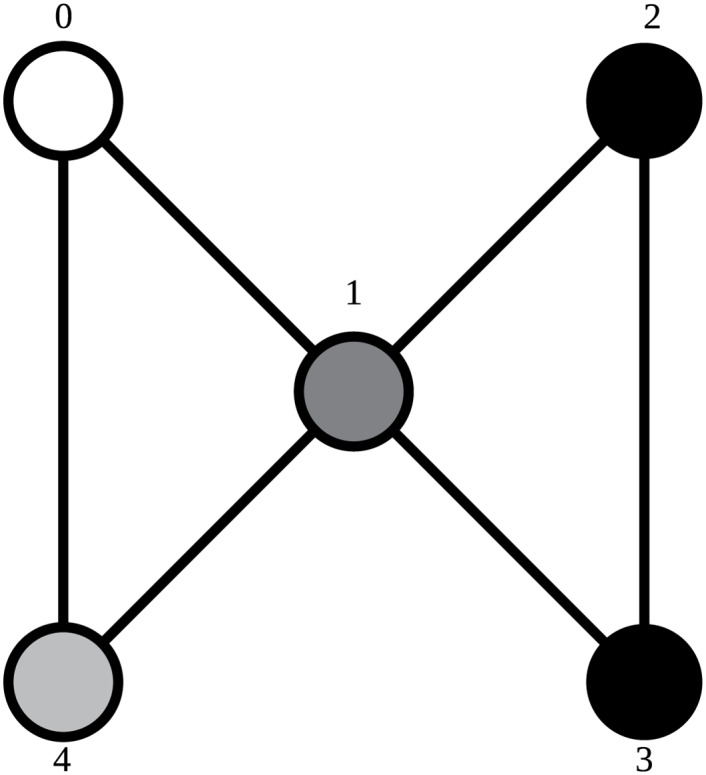
Orbit representative 43. Vertex 0 is marked and cannot be permuted. Vertices colored the same shade of gray are in the same suborbit.

The suborbits of orbit representative 44 are the same as the orbits of graphlet 18: in Fig 6 the central, marked vertex is one suborbit, the other vertices constitute another. In orbit representative 43, the suborbits are different, despite both orbit representatives being part of the same graphlet. In Fig 7, the upper left vertex is marked and called vertex 0, and cannot be interchanged with any other vertex during permutations. Vertex 1 is still in an orbit alone, but not all other vertices are together in an orbit. Vertex 0 is never interchanged, so it is the only vertex in its orbit by definition. No matter which of the allowed automorphisms is used, vertex 4 cannot be mapped on any other vertex, putting it in its own orbit as well. Vertices 2 and 3 can still be swapped, so they belong in the same orbit.

In [6], equations are composed by considering different ways vertices can be added to graphlets. Likewise, in creating the equations, vertices will be added to orbit representatives. This will be noted as follows:

$$\begin{array}{ccc} G\left(n\right)\cup \{\left\{x,v_{1}\right\},\left\{x,v_{2}\right\},...|v_{m}\in V\left(G\left(n\right)\right)\} & = & \{V(G(n))\cup x,\\ & & E\left(G\left(n\right)\right)\cup \left\{\left\{x,v_{1}\right\},\left\{x,v_{2}\right\},...\right\}\}. \end{array}$$ (12)

This means a new vertex *x* is added to the graphlet, along with a set of edges connecting the new vertex to some of the graphlet’s other vertices. Likewise, edges can be added to the graphlet:

$$\begin{array}{ccc} G\left(n\right)\cup \{\left\{v_{1},v_{2}\right\},\left\{v_{3},v_{4}\right\},...|v_{m}\in V\left(G\left(n\right)\right)\} & = & \{V(G(n)),\\ & & E\left(G\left(n\right)\right)\cup \left\{\left\{v_{1},v_{2}\right\},\left\{v_{3},v_{4}\right\},...\right\}\}. \end{array}$$ (13)

These new edges then each connect two vertices that were already present in the graphlet.

### Two examples of equation construction

Now that the needed theory is shown, the equations to count graphlets can be composed. The following two examples illustrate how an equation can be constructed. Both show an orbit representative in a 3-graphlet to which an extra vertex is added. When the vertices being part of such an orbit representative can be identified within an explored graph, adding common neighbors of those vertices to the 3-graphlets forms various 4-graphlets.

#### A simple example

**Fig 8:**
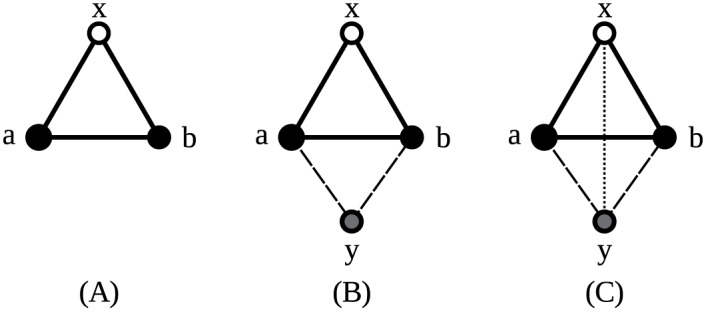
Construction of an equation. The white vertex is the orbit representative’s marked vertex, black vertices were present in the original orbit representative and the gray vertex is the new vertex. Full lines indicate the original edges, dashed lines the edges added as part of the common neighbors and dotted lines the vertices that are added afterwards. (A) *G*(*x*)≃ Orbit representative 3. (B) *G*(*x*)∪{{*y*, *a*}, {*y*, *b*}}≃ Orbit representative 12. (C) *G*(*x*)∪{{*y*, *a*}, {*y*, *b*}, {*y*, *x*}}≃ Orbit representative 14.

In Fig 8, the construction of an equation is shown. Panel A shows orbit representative 3 (seen in Fig 1), to which a new vertex will be added. In panel B, the new vertex is added, with edges to vertices *a* and *b*. These edges can be chosen at will; connecting the new vertex to a different set of neighbors results in a different equation. In panel C, it is additionally connected to vertex *x*, after which there are no more vertices it can be connected to.

As such, all orbits involved in the construction of this equation are known. If a vertex *x* within an explored graph touches orbit 3, and the other two vertices of that orbit representative have a certain number of common neighbors, these common neighbors will transform orbit 3 either in orbit 12 or 14. However, vertex *x* is itself a common neighbor of *a* and *b*, as well. This means that *x* will also be counted when searching for the common neighbors of those vertices within the explored graph. Therefore, the actual number of common neighbors available to create orbits 12 and 14 will be one less.

There still is one catch: to avoid counting graphlets twice, the vertices a and b will have a unique ordering. As they are in the same suborbit, they are interchangeable and if no unique ordering is imposed on the indices of the vertices mapped on them while finding the graphlets, each instance of the graphlet will be found twice. Therefore, *a* and *b* need a fixed ordering. No such ordering is imposed on the newly added vertex, however. So, assume we are looking at a graph in which vertices with indices 0, 1, 2, 3 form orbit representative 14. Orbit representative 3 is formed by vertices 0, 1, 2. Vertices 1 and 2 have common neighbors 0 and 3, of which we discard one. Likewise, vertices 0, 1, 3 form orbit representative 3, in which we can find common vertex 2 and discard 0. The same story applies to vertices 0, 2, 3. No further orbit representatives 3 are found because of the ordering imposed on vertex *a* and *b*. However, the same orbit representative was counted three times. To compensate for this, the term containing orbit 14 will need to be multiplied by 3.

Now all possible symmetrical situations have been explored. The resulting equation reads:

$$\begin{array}{c} o_{12}+3o_{14}=\sum_{\left\{x,a,b\right\}=P_{3}}\left(c\left(a,b\right)-1\right) \end{array}$$ (14)

in which *o*_12_ and *o*_14_ are the number of times a chosen vertex *x* in the explored graph touches orbits 12 and 14, respectively. The range of the sum, {*x*, *a*, *b*} = *P*_3_, means all sets of vertices that form a graphlet in which node x touches orbit 3. In this case, these are all instances of graphlet 2 that are touched by node *x*. In less symmetric graphlets, only the instances in which node *x* touches the specific orbit are counted. The number of common neighbors of a and b is notated as *c*(*a*, *b*).

**Fig 9:**
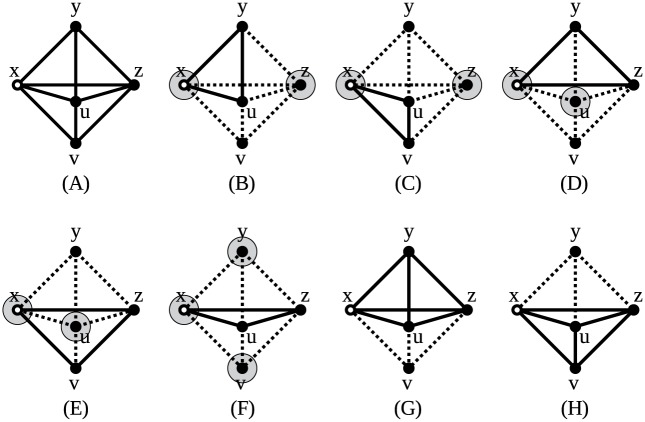
Use of an equation. (A) The explored graph in which Eq (14) is tested. Vertex *x*, colored white, is the inspected vertex. (B-F) The graph is shown in dotted lines, edges in full lines show all different graphlets where vertex *x* touches orbit 3. Vertices on a gray background are common neighbors of both other vertices of those graphlets. (G-H) The two graphlets in which vertex *x* touches orbit 14.

As a test for Eq (14), Fig 9 shows how the equation would be solved in a small graph, shown in panel A. Vertex *x* is the inspected vertex. The right-hand side of Eq (14) indicates that all graphlets in which vertex *x* touches orbit 3 must be listed. Panels B to F show all different triangles vertex *x* touches, thereby touching orbit 3. Furthermore, all common neighbors of the other two vertices in the triangle have a gray background. As can be seen, panels B to E all show 2 common neighbors, while panel F shows three. As the right-hand side of the equation dictates that these should all be subtracted by 1, then added, the right-hand side sums up to 6.

The number of times vertex *a* touches orbit 14 is 2, as can be seen in Fig 9, panel G and H. Plugging this value in Eq (14) gives

$$\begin{array}{c} o_{12}+3o_{14}=\sum_{P_{3}}\left(c\left(a,b\right)-1\right) \end{array}$$ (15)

$$\begin{array}{c} o_{12}+3*2=6 \end{array}$$ (16)

$$\begin{array}{c} o_{12}=0 \end{array}$$ (17)

meaning vertex *a* does not touch orbit 12. Indeed, vertex a has an edge to every other vertex in the graphlet, which means it can not touch orbit 12.

#### A more complicated example

**Fig 10:**
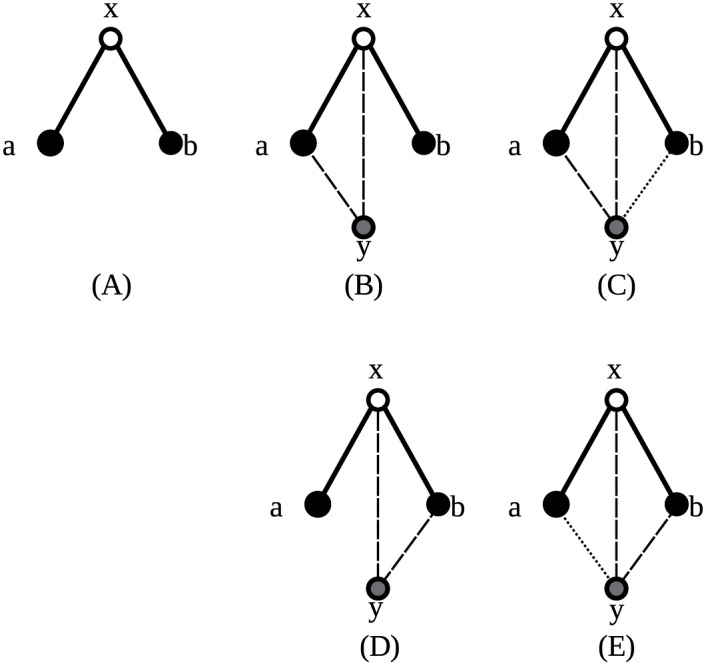
Construction of an equation. (A) *G*(*x*)≃ Orbit representative 2. (B) *G*′(*x*) = *G*(*x*)∪{{*y*, *x*}, {*y*, *a*}}≃ Orbit representative 11. (C) *G*′(*x*)∪{*y*, *b*}≃ Orbit representative 13. (D) *G*′′(*x*) = *G*(*x*)∪{{*y*, *x*}, {*y*, *b*}}≃ Orbit representative 11. (E) *G*′′(*x*)∪{*y*, *a*} = *G*′(*x*)∪{*y*, *b*}.

A second example shows another complication. Fig 10 shows how orbit representatives 11 and 13 are created by adding a vertex to orbit representative 2. In panel A, the new vertex is connected to vertices *x* and *a* of orbit representative 2, creating orbit representative 11. However, this is not the only way this orbit representative can be created from orbit representative 2. Adding a new vertex connected to vertices *x* and *b*, as is done in panel C, also creates a valid orbit representative 11. Indeed, because vertices *a* and *b* are in the same suborbit of orbit representative 2, they will have a unique ordering. In orbit representative 11, they are not in the same suborbit, so they need to be considered separately. Therefore, the sum in the right-hand side of the equation will contain two different terms: one for either way the new vertex can be connected.

As in the previous example, the added vertex shares a suborbit of orbit representative 11 with another vertex. Therefore, the term counting orbit representative 11 needs to be multiplied by 2.

When the new vertex is additionally connected to vertex *b*, as is done in panel B, orbit representative 13 is created. This way, the added vertex is not in the same suborbit as any other vertex anymore. Similarly, the orbit representative 11 in panel C becomes orbit representative 13 in panel D with an additional edge to vertex *a*. Now those two orbit representatives are exactly the same. In other words, any orbit representatives 13 are counted twice in this way. The solution is to multiply the orbit representative 13 term by 2 to compensate.

The previous reasoning gives rise to the equation:

$$\begin{array}{c} 2o_{11}+2o_{13}=\sum_{P_{2}}c\left(x,a\right)+c\left(x,b\right) \end{array}$$ (18)

### Generating the equations

Now the reasoning of the previous section can be generalized. An orbit representative in a (n-1)-graphlet serves as base to construct some equations for counting vertices touching orbits in n-graphlets. A new vertex is added to the orbit representative, having edges to a set of some, but not all, other vertices in the orbit representative. Then, all possible combinations of other edges are added to the orbit representative. Each different orbit representative which is created in this way will appear in a term in the left-hand side of the equation. Even if multiple isomorphic orbit representatives are created multiple times, this only creates a single term. This is due to the fact that those different situations will all be seen as the same orbit when counting how many times a vertex within an explored graph touches an orbit.

**function** generateEquation(OrbitRepresentative start, Set ⟨Vertex⟩ neighbors)

 Set⟨OrbitRepresentative⟩ lhsOrbits := ∅

 **for all** Set⟨Vertex⟩ s **in** (start.vertices—neighbors) **do**

  Set ⟨Vertex⟩ connections := neighbors ∪ s

  lhsOrbits := lhsOrbits ∪ (start ∪ connections)

 **end for**

 lhs := 0

 rhs := 0

 **for all** OrbitRepresentative rep **in** lhsOrbits **do**

  lhs := lhs + rep * lhsFactor(rep)

 **end for**

 rhsSum := start

 rhs := rhs + neighbors

 rhs := rhs—minusTerm(start, neighbors)

 **return** lhs = rhs

**end function**

To determine the factor by which each term must be multiplied, the suborbits of that orbit representative are sought. Then the term is multiplied by the size of the suborbit in which the new vertex is located. After all, all vertices from that suborbit, except the new one, come from the same suborbit in the smaller graphlet. Then, it can be assumed that they have a fixed order when the orbit representative is found in the graph. The new vertex, however, is not required to be in any particular position in this order. Therefore, it may be in any of the *n* positions, and the term for each orbit representative must be multiplied by its size.

**function** lhsFactor(OrbitRepresentative rep)

 Calculate rep’s suborbits

 **return** size of the last vertex’s suborbit

**end function**

The right-hand side is constructed purely from the original orbit representative and the vertices that are connected to the new vertex. Evidently they are seen in the orbit representative over which the sum must be made and the term containing the common neighbors, respectively. Potential negative terms in the sum are equal to the number of vertices satisfying the conditions imposed by the common neighbor term. For instance, if an equation has a term specifying the common neighbors of *a* and *b*, which have 2 common neighbors in the original graphlet, the negative term should be -2.

**function** minusTerm(OrbitRepresentative start, Set 〈Vertex〉 neighbors)

 result := 0

 **for all** Vertex n1 **in** start.vertices **do**

  connected := true

  **for all** Vertex n2 **in** neighbors **do**

  **if** {*n*1, *n*2}∉ start.edges **then**

   connected := false

  **end if**

 **end for**

 **if** connected **then**

   result := result + 1

  **end if**

 **end for**

 **return** result

**end function**

This entire procedure is performed for all possible combinations of common neighbors of vertices in the original graphlet. When two equations’ orbit representatives are all equal, the situation of the second example applies and the two equations describe symmetrical ways the same orbit representatives can be created from a starting graphlet. These equations then need to be merged. This means a new equation is created containing the same orbit representatives, with the sum of both equations’ right-hand sides as the new equation’s right-hand side. Also, when a corresponding pair of orbit representatives in the left-hand sides of the two equations has identical lists of edges, their multiplication factors need to be added to avoid counting the same orbit representative twice.

**function** merge(Equation e1, Equation e2)

 lhs := 0

 **for** i = 0 **to** e1.lhs.size **do**

  **if** e1.lhs.getOrbit(i) == e2.lhs.getOrbit(i) **then**

   lhs := lhs + e1.lhs.getTerm(i)+e2.lhs.getTerm(i)

  **else**

  lhs := e1.lhs.getTerm(i)

  **end if**

 **end for**

  rhs := e1.rhs + e2.rhs

 **return** lhs = rhs

**end function**

### Selection of a linearly independent system of equations

As is mentioned in Hočevar’s paper, the method used here to generate the equations gives rise to a large amount of linearly dependent equations. For instance: 114 equations are generated to enable counting orbits of 5-graphlets. There are only 58 orbits in 5-graphlets, and one of them still needs to be counted [6], which means 57 equations are needed. As a result, the need arises to find a criterium to select a linearly independent set of equations.

To each orbit representative, except the sole orbit of the complete graphlet, an edge can be added, making another orbit representative. Graphlets, and by extension their orbits, are ordered by number of edges in Pržulj’s system; therefore, adding an edge increases the number of the orbit. Each equation relates orbits that are created by adding a new vertex and its edges to a smaller graphlet, of which some must be present, while the others may or may not be present. As a result, the orbit in which only the obligatory edges are present will have the lowest number in Pržulj’s identification.

This observation shows that each orbit will be the orbit with the smallest number in at least one equation, with exception of the complete graphlet. Therefore, creating a linearly independent system boils down to selecting such an equation for each graphlet. These equations will then be linearly independent and straightforward to solve. Indeed, to solve an equation, it suffices to solve the equations in descending order and fill in the solutions to the previous equations; no further reduction of the system is needed to solve it.

**function** generateEquations(int size)

 List ⟨Equation⟩ equations

 **for all** OrbitRepresentative rep **of** size—1 **do**

  **for all** Set ⟨Vertex⟩ s **in** g.vertices **do**

   e1 = generateEquation(rep,s)

   **if** equations[e1.lowestOrbit] == null **then**

    equations[e1.lowestOrbit] := e1

   **else if** equations[e1.lowestOrbit].graphlets == e1.graphlets **then**

    equations[e1.lowestOrbit] := merge(e1,equations[e1.lowestOrbit])

   **end if**

  **end for**

 **end for**

 **return** equations

**end function**

### Going to higher order graphlets

The presented method can generate equations for graphlets of any order. However, the method used to find a linearly independent set, as well as the actual interpretation of the equations, need an actual naming scheme for the generated orbits. Like done before, we will give each graphlet and orbit a number. Unlike the graphlet numbering used before, however, we will try to generate the ordering of the graphlets automatically. This way, the numbering can be extended to larger graphlets without the need to change the code.

The criteria for selection of an independent system of equations impose a partial ordering on the orbits’ numbers. If one orbit can be created by adding an edge to another, the first must have a higher number than the second. Orbits that cannot be created from each other by only adding or only removing edges do not need a particular ordering. To simplify matters, first an ordering for graphlets will be made, then the orbits within each graphlet will be ordered.

**Fig 11:**
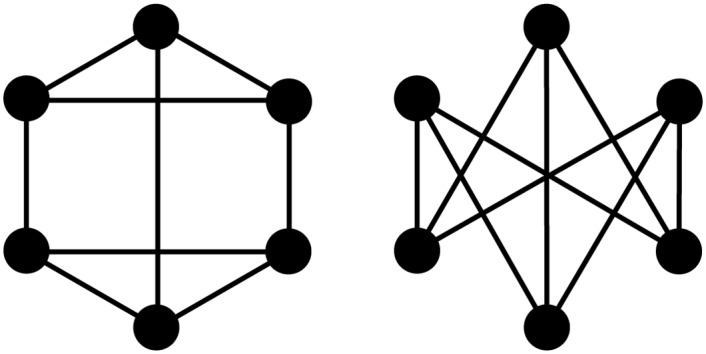
Two 6-graphlets. All of both graphlets’ vertices have degree 3, and each graphlet has one orbit containing all of its vertices. (A) This graphlet’s edges and vertices correspond to the edges and vertices in a triangular prism. (B) The complete bipartite graph *K*_3,3_.

Choosing to apply intuitive rules, that can be easily checked at first sight by an observer, translates poorly to larger graphlets. For example, both 6-graphlets in Fig 11 have six vertices of degree 3, which are in a single orbit, but the graphlets themselves are different. The difference can easily be spotted: the prism graphlet (panel A) contains two triangles, while *K*_3,3_ (panel B) does not. As such, they cannot be discriminated on basis of number of edges, degree of vertices, orbits or which type of vertices are connected. One can imagine this kind of problem will only get worse with increasing order.

**Fig 12:**
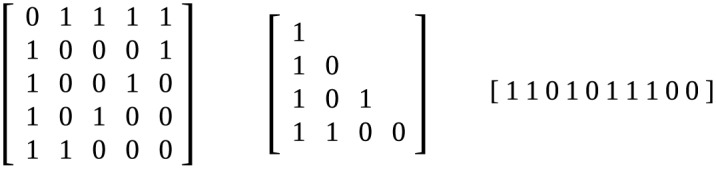
Matrix form, triangular matrix form and string form of graphlet 18. These forms correspond to graphlet 18 as it is shown in Fig 5. The string form is not the lexicographically smallest possible for this graphlet.

To avoid such problems, graphlets are represented in triangular matrix form. In matrix form, an n-graphlet is represented as an *n* ∗ *n* matrix. The value at row *r* and column *c* is 1 if there is an edge between vertex *r* and *c* and 0 if there is not. In Fig 12, the matrix corresponding to the graphlet in Fig 5 can be seen. As graphlets are undirected, the matrix is symmetrical. Additionally, all values on the primary diagonal are 0 because no self-loops are allowed. Therefore, we are able to discard the diagonal and every value above it, reducing the amount of values that have to be saved for each graphlet. The result of this operation can be seen in the second matrix in Fig 12. Then, all values in the triangular matrix are saved in a single string. This is done for all possible permutations of the vertices of a graphlet, and the string that is lexicographically smallest is kept. This string is a unique identifier for any graphlet.

If such a string is made for each graphlet of a certain order, and those strings are sorted lexicographically, a unique ordering for the graphlets is established. Any added edge will change a 0 into a 1, which will make the string lexicographically larger, and the new graphlet’s number will therefore be larger than the old one’s. This means that using this ordering to number the graphlets gives rise to a numbering following the partial ordering that was required.

In practice, the ordering was made by generating all possible string forms of graphlets of a certain size in order with a binary counter. These are then converted to actual graphlets and added to an ordered set so they keep their ordering but duplicates are not allowed.

The orbits are numbered in the order that their vertices first appear in the lexicographically smallest string representation. Vertex 0 in this representation will always be in the first orbit, vertex 1 can either be in the same orbit or start orbit 2, and so on.

## Results and Discussion

### Equations

The equations generated for 4-graphlets can be found in S1 Equations. They differ from the ones in [6], because here new vertices cannot be added in a path containing two already present vertices. Instead, any neighbor of any vertex already present can be added. However, both sets of equations are equivalent. For instance, the two equations:

$$\begin{array}{c} o_{4}+2o_{8}+2o_{9}+2o_{12}=\sum_{P_{1}}\left(c\left(b\right)-1\right) \end{array}$$ (19)

$$\begin{array}{c} 2o_{9}+2o_{12}=\sum_{P_{1}}c\left(a,b\right) \end{array}$$ (20)

can be subtracted from each other, resulting in

$$\begin{array}{c} o_{4}+2o_{8}=\sum_{P_{1}}\left(c\left(b\right)-c\left(a,b\right)-1\right)=\sum_{P_{1}}p\left(a,b\right) \end{array}$$ (21)

where *p*(*a*, *b*) is the number of vertices that form 3-vertex paths starting with *a* and *b*, i.e. all neighbors of *b* but not *a*, except *a* itself. This equation is the corresponding equation that appears in [6].

Similarly, the equations for 5-graphlets, which can be found in S2 Equations, are different from the ones in [6]. This is purely due to the process of selecting a linearly independent system. When no such selection is made, a large, linearly dependent system of equations is generated, which does contain all of the equations in [6].

The new equations for 6-graphlets are shown in S3 Equations. The algorithm to generate the equations is order-independent and can generate equations to facilitate counting of graphlets of any size. This is the first time that equations to count 6-graphlets were derived; this marks an important step for the use of larger graphlets.

### Graphlet naming

#### Graphlets up to order 5

**Fig 13:**
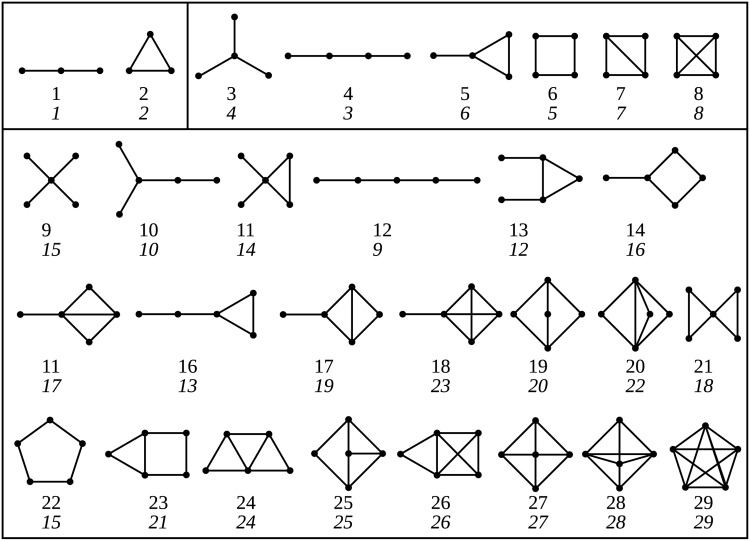
All graphlets up to order 5. The numbers in normal font are the graphlet ordering generated by the algorithm, the numbers in italic font are Pržulj’s ordering.

As is expected, most of the graphlets up to order 5 have a different naming here than in Pržulj’s scheme. This is shown in Fig 13.

For ease of comparison between the automatically generated equations and the equations in [6], the original naming scheme was used in the previous parts of this article, wherever possible. The generated numbering was only used when describing 6-graphlets, which have no place in the original numbering.

#### Graphlets of order 6

All graphlets and orbits of order 6 were identified. A total of 112 graphlets and 407 orbits were identified and can be found in S1 Fig. Like the algorithm to generate the equations, this algorithm is order-independent and can therefore be used to order graphlets of any size.

## Conclusion

A new algorithm has been developed to automatically generate equations that facilitate counting the number of times each node of an explored graph touches each orbit of graphlets of a certain order. This algorithm can create an independent set of equations to calculate graphlet degree distributions without restriction of the order of the graphlets. In addition, a new algorithm to automatically name graphlets and orbits has been created, which enables the use of higher-order graphlets. Both algorithms were programmed in Java, the source code is available at https://github.com/IneMelckenbeeck/equation-generator and https://github.com/IneMelckenbeeck/graphlet-naming.

All 6-graphlets were ordered and named by this algorithm, and the equations that enable efficient counting of their orbits were generated. This is a large step towards using graphlets with an order of 6 and larger.

### Future work

The algorithms presented in this paper enable the use of an ORCA-like counter for graphlets of any size. However, the ORCA code itself is highly optimized for counting 4- and 5-graphlets, making it impossible to use the equations for any other order with the existing code. The next step is thus to develop an efficient and order-independent algorithm that makes efficient use of these equations to actually count the times each vertex in an explored graph touches each orbit.

## Supporting Information

S1 Equations Generated equations for 4-graphlets. (PDF)

S2 Equations Generated equations for 5-graphlets. (PDF)

S3 Equations Generated equations for 6-graphlets. (PDF)

S1 Fig Graphlets of order 6. All graphlets of order 6 are shown in the newly introduced order. In the lower left corner of each page, the graphlet’s orbits are listed in their order.
(PDF)
