# Supplementary material for: An Algorithm to Automatically Generate the Combinatorial Orbit Counting Equations
Source: PLoS One. 2016 Jan 21;11(1):e0147078. doi: 10.1371/journal.pone.0147078 (PMC4721873; doi:10.1371/journal.pone.0147078)
Supplement: S1 Equations — (PDF) [file pone.0147078.s001.pdf]

Equations to count orbits in 4-graphlets

$$P_1(x,a,b) = \{\{x,a,b\} \subset V(G) | \{\{x,a\},\{a,b\}\} \subset E(G) \wedge \{\{x,b\}\} \cap E(G) = \emptyset\}$$

$$P_2(x,a,b) = \{\{x,a,b\} \subset V(G) | \{\{x,a\},\{x,b\}\} \subset E(G) \wedge \{\{a,b\}\} \cap E(G) = \emptyset\}$$

$$P_3(x,a,b) = \{\{x,a,b\} \subset V(G) | \{\{x,a\},\{x,b\},\{a,b\}\} \subset E(G)\}$$

$$o_4 + 2o_8 + 2o_9 + 2o_{12} = \sum_{P_1} (c(b) - 1)$$

$$o_5 + o_{10} + 2o_8 + 2o_{12} = \sum_{P_1} (c(x) - 1)$$

$$2o_6 + o_{10} + 2o_9 + 2o_{12} = \sum_{P_1} (c(a) - 2)$$

$$3o_7 + 2o_{11} + o_{13} = \sum_{P_2} (c(x) - 2)$$

$$2o_8 + 2o_{12} = \sum_{P_1} (c(x,b) - 1)$$

$$2o_9 + 2o_{12} = \sum_{P_1} c(a,b)$$

$$o_{10} + 2o_{12} = \sum_{P_1} c(x,a)$$

$$2o_{11} + 2o_{13} = \sum_{P_2} c(x,a) + c(x,b)$$

$$o_{12} + 3o_{14} = \sum_{P_3} (c(a,b) - 1)$$

$$2o_{13} + 6o_{14} = \sum_{P_3} (c(x,a) - 1) + (c(x,b) - 1)$$
