# Supplementary material for: An Algorithm to Automatically Generate the Combinatorial Orbit Counting Equations
Source: PLoS One. 2016 Jan 21;11(1):e0147078. doi: 10.1371/journal.pone.0147078 (PMC4721873; doi:10.1371/journal.pone.0147078)
Supplement: S2 Equations — (PDF) [file pone.0147078.s002.pdf]

Equations to count orbits in 5-graphlets

$$
\begin{aligned}
P_5(x,a,b,c) = \{\{x,a,b,c\} \subset V(G)| \\
\{\{x,a\},\{x,c\},\{a,b\}\} \subset E(G) \wedge \\
\{\{x,b\},\{a,c\},\{b,c\}\} \cap E(G) = \emptyset\}
\end{aligned}
$$

$$
\begin{aligned}
P_7(x,a,b,c) = \{\{x,a,b,c\} \subset V(G)| \\
\{\{x,a\},\{x,b\},\{x,c\}\} \subset E(G) \wedge \\
\{\{a,b\},\{a,c\},\{b,c\}\} \cap E(G) = \emptyset\}
\end{aligned}
$$

$$
\begin{aligned}
P_4(x,a,b,c) = \{\{x,a,b,c\} \subset V(G)| \\
\{\{x,a\},\{a,b\},\{b,c\}\} \subset E(G) \wedge \\
\{\{x,b\},\{x,c\},\{a,c\}\} \cap E(G) = \emptyset\}
\end{aligned}
$$

$$
\begin{aligned}
P_6(x,a,b,c) = \{\{x,a,b,c\} \subset V(G)| \\
\{\{x,a\},\{a,b\},\{a,c\}\} \subset E(G) \wedge \\
\{\{x,b\},\{x,c\},\{b,c\}\} \cap E(G) = \emptyset\}
\end{aligned}
$$

$$
\begin{aligned}
P_8(x,a,b,c) = \{\{x,a,b,c\} \subset V(G)| \\
\{\{x,a\},\{x,c\},\{a,b\},\{b,c\}\} \subset E(G) \wedge \\
\{\{x,b\},\{a,c\}\} \cap E(G) = \emptyset\}
\end{aligned}
$$

$$
\begin{aligned}
P_{11}(x,a,b,c) = \{\{x,a,b,c\} \subset V(G)| \\
\{\{x,a\},\{x,b\},\{x,c\},\{a,c\}\} \subset E(G) \wedge \\
\{\{a,b\},\{b,c\}\} \cap E(G) = \emptyset\}
\end{aligned}
$$

$$
\begin{aligned}
P_9(x,a,b,c) = \{\{x,a,b,c\} \subset V(G)| \\
\{\{x,a\},\{a,b\},\{a,c\},\{b,c\}\} \subset E(G) \wedge \\
\{\{x,b\},\{x,c\}\} \cap E(G) = \emptyset\}
\end{aligned}
$$

$$
\begin{aligned}
P_{10}(x,a,b,c) = \{\{x,a,b,c\} \subset V(G)| \\
\{\{x,a\},\{x,c\},\{a,b\},\{a,c\}\} \subset E(G) \wedge \\
\{\{x,b\},\{b,c\}\} \cap E(G) = \emptyset\}
\end{aligned}
$$

$$
\begin{aligned}
P_{13}(x,a,b,c) = \{\{x,a,b,c\} \subset V(G)| \\
\{\{x,a\},\{x,b\},\{x,c\},\{a,c\},\{b,c\}\} \subset E(G) \wedge \\
\{\{a,b\}\} \cap E(G) = \emptyset\}
\end{aligned}
$$

$$P_{12}(x,a,b,c) = \{\{x,a,b,c\} \subset V(G) | \\ \{\{x,a\},\{x,c\},\{a,b\},\{a,c\},\{b,c\}\} \subset E(G) \wedge \\ \{\{x,b\}\} \cap E(G) = \emptyset\}$$

$$P_{14}(x,a,b,c) = \{\{x,a,b,c\} \subset V(G) | \\ \{\{x,a\},\{x,b\},\{x,c\},\{a,b\},\{a,c\},\{b,c\}\} \subset E(G)\}$$

$$o_{15} + 2o_{27} + 2o_{34} + 2o_{35} + 2o_{45} + o_{51} + 2o_{52} + o_{59} = \sum_{P_4(x,a,b,c)} (c(c) - 1)$$

$$o_{16} + o_{29} + 2o_{34} + 2o_{36} + 2o_{46} + o_{51} + 2o_{52} + o_{59} = \sum_{P_4(x,a,b,c)} (c(x) - 1)$$

$$2o_{17} + 2o_{30} + 2o_{34} + o_{37} + o_{48} + o_{51} + o_{53} + o_{60} = \sum_{P_5(x,a,b,c)} (c(c) - 1)$$

$$2o_{18} + o_{24} + 2o_{27} + 2o_{36} + 2o_{45} + 2o_{46} + o_{51} + o_{59} = \sum_{P_4(x,a,b,c)} (c(b) - 2)$$

$$o_{19} + 2o_{31} + 2o_{35} + o_{37} + 2o_{39} + o_{40} + 4o_{49} + 4o_{54} = \sum_{P_6(x,a,b,c)} (c(b) - 1) + (c(c) - 1)$$

$$o_{20} + o_{32} + o_{37} + o_{40} + 2o_{49} + 2o_{54} = \sum_{P_6(x,a,b,c)} (c(x) - 1)$$

$$o_{21} + 2o_{33} + 2o_{38} + 2o_{42} + 3o_{50} + 3o_{55} = \sum_{P_7(x,a,b,c)} (c(a) - 1) + (c(b) - 1) + (c(c) - 1)$$

$$3o_{22} + 2o_{31} + o_{32} + o_{39} + o_{40} + 2o_{54} = \sum_{P_6(x,a,b,c)} (c(a) - 3)$$

$$4o_{23} + 2o_{33} + o_{42} + o_{55} = \sum_{P_7(x,a,b,c)} (c(x) - 3)$$

$$o_{24} + 2o_{45} + 2o_{46} + o_{59} = \sum_{P_4(x,a,b,c)} c(a,b)$$

$$2o_{25} + o_{40} + o_{48} + 2o_{52} + 2o_{57} + o_{59} + 2o_{64} + 2o_{66} = \sum_{P_{10}(x,a,b,c)} (c(c) - 2)$$

$$o_{26} + 2o_{47} + o_{48} + o_{60} = \sum_{P_5(x,a,b,c)} c(x,a)$$

$$2o_{27} + 2o_{45} + o_{51} + o_{59} = \sum_{P_4(x,a,b,c)} c(b,c)$$

$$2o_{28} + 2o_{47} + o_{51} + o_{60} = \sum_{P_5(x,a,b,c)} c(a,b)$$

$$o_{29} + 2o_{46} + 2o_{52} + o_{59} = \sum_{P_4(x,a,b,c)} c(x,a)$$

$$2o_{30} + o_{48} + o_{53} + o_{60} = \sum_{P_5(x,a,b,c)} c(x,c)$$

$$2o_{31} + 2o_{39} + o_{40} + 4o_{54} = \sum_{P_6(x,a,b,c)} c(a,b) + c(a,c)$$

$$o_{32} + o_{40} + 2o_{54} = \sum_{P_6(x,a,b,c)} c(x,a)$$

$$2o_{33} + 2o_{42} + 3o_{55} = \sum_{P_7(x,a,b,c)} c(x,a) + c(x,b) + c(x,c)$$

$$2o_{34} + o_{51} + 2o_{52} + o_{59} = \sum_{P_4(x,a,b,c)} c(x,c)$$

$$o_{35} + o_{39} + 2o_{49} + 2o_{54} = \sum_{P_6(x,a,b,c)} (c(b,c) - 1)$$

$$2o_{36} + 2o_{46} + o_{51} + o_{59} = \sum_{P_4(x,a,b,c)} (c(x,b) - 1)$$

$$o_{37} + o_{40} + 4o_{49} + 4o_{54} = \sum_{P_6(x,a,b,c)} (c(x,b) - 1) + (c(x,c) - 1)$$

$$o_{38} + o_{42} + 3o_{50} + 3o_{55} = \sum_{P_7(x,a,b,c)} (c(a,b) - 1) + (c(a,c) - 1) + (c(b,c) - 1)$$

$$o_{39} + 2o_{54} = \sum_{P_6(x,a,b,c)} c(a,b,c)$$

$$o_{40} + 4o_{54} = \sum_{P_6(x,a,b,c)} c(x,a,b) + c(x,a,c)$$

$$2o_{41} + 2o_{57} + o_{60} + 2o_{66} = \sum_{P_{10}(x,a,b,c)} (c(x,a) - 1)$$

$$o_{42} + 3o_{55} = \sum_{P_7(x,a,b,c)} c(x,a,b) + c(x,a,c) + c(x,b,c)$$

$$2o_{43} + o_{59} + o_{60} + 2o_{66} = \sum_{P_{10}(x,a,b,c)} c(a,b)$$

$$4o_{44} + 2o_{61} + o_{67} = \sum_{P_{11}(x,a,b,c)} c(x,b)$$

$$2o_{45} + o_{59} = \sum_{P_4(x,a,b,c)} c(a,b,c)$$

$$2o_{46} + o_{59} = \sum_{P_4(x,a,b,c)} c(x,a,b)$$

$$2o_{47} + o_{60} = \sum_{P_5(x,a,b,c)} c(x,a,b)$$

$$o_{48} + o_{60} = \sum_{P_5(x,a,b,c)} c(x,a,c)$$

$$2o_{49} + 2o_{54} = \sum_{P_6(x,a,b,c)} (c(x,b,c) - 1)$$

$$o_{50} + o_{55} = \sum_{P_7(x,a,b,c)} (c(a,b,c) - 1)$$

$$o_{51} + o_{59} = \sum_{P_4(x,a,b,c)} c(x,b,c)$$

$$2o_{52} + o_{59} = \sum_{P_4(x,a,b,c)} c(x,a,c)$$

$$o_{53} + o_{60} = \sum_{P_5(x,a,b,c)} c(x,b,c)$$

$$2o_{54} + 2o_{65} + o_{66} + 3o_{70} = \sum_{P_{12}(x,a,b,c)} (c(a,c) - 2)$$

$$3o_{55} + 2o_{67} + 2o_{71} = \sum_{P_{13}(x,a,b,c)} (c(x,c) - 2)$$

$$3o_{56} + 2o_{65} = \sum_{P_9(x,a,b,c)} c(a,b,c)$$

$$o_{57} + 2o_{66} + o_{67} + 3o_{70} + 4o_{71} + 12o_{72} = \sum_{P_{14}(x,a,b,c)} (c(a) - 3) + (c(b) - 3) + (c(c) - 3)$$

$$o_{58} + o_{67} + 2o_{71} + 4o_{72} = \sum_{P_{14}(x,a,b,c)} (c(x) - 3)$$

$$o_{59} + 2o_{66} = \sum_{P_{10}(x,a,b,c)} c(a,b,c)$$

$$o_{60} + 2o_{66} = \sum_{P_{10}(x,a,b,c)} c(x,a,b)$$

$$2o_{61} + 2o_{67} = \sum_{P_{11}(x,a,b,c)} c(x,a,b) + c(x,b,c)$$

$$2o_{62} + 2o_{65} = \sum_{P_{9}(x,a,b,c)} (c(x,b,c) - 1)$$

$$o_{63} + o_{67} = \sum_{P_{11}(x,a,b,c)} (c(a,b,c) - 1)$$

$$2o_{64} + 2o_{66} = \sum_{P_{10}(x,a,b,c)} (c(x,b,c) - 1)$$

$$2o_{65} + 3o_{70} = \sum_{P_{12}(x,a,b,c)} c(a,b,c)$$

$$o_{66} + 3o_{70} + 2o_{71} + 12o_{72} = \sum_{P_{14}(x,a,b,c)} (c(a,b) - 2) + (c(a,c) - 2) + (c(b,c) - 2)$$

$$o_{67} + 4o_{71} + 12o_{72} = \sum_{P_{14}(x,a,b,c)} (c(x,a) - 2) + (c(x,b) - 2) + (c(x,c) - 2)$$

$$2o_{68} + 6o_{70} = \sum_{P_{12}(x,a,b,c)} (c(x,a,b) - 1) + (c(x,b,c) - 1)$$

$$4o_{69} + 2o_{71} = \sum_{P_{13}(x,a,b,c)} (c(x,a,b) - 1)$$

$$o_{70} + 4o_{72} = \sum_{P_{14}(x,a,b,c)} (c(a,b,c) - 1)$$

$$2o_{71} + 12o_{72} = \sum_{P_{14}(x,a,b,c)} (c(x,a,b) - 1) + (c(x,a,c) - 1) + (c(x,b,c) - 1)$$
