# Supplementary material for: An Algorithm to Automatically Generate the Combinatorial Orbit Counting Equations
Source: PLoS One. 2016 Jan 21;11(1):e0147078. doi: 10.1371/journal.pone.0147078 (PMC4721873; doi:10.1371/journal.pone.0147078)
Supplement: S3 Equations — (PDF) [file pone.0147078.s003.pdf]

Equations to count orbits in 6-graphlets

$$P_{15}(x,a,b,c,d) = \{\{x,a,b,c,d\} \subset V(G) | \{\{x,a\},\{a,b\},\{b,c\},\{c,d\}\} \subset E(G) \wedge \{\{x,b\},\{x,c\},\{x,d\},\{a,c\},\{a,d\},\{b,d\}\} \cap E(G) = \emptyset\}$$

$$P_{16}(x,a,b,c,d) = \{\{x,a,b,c,d\} \subset V(G) | \{\{x,a\},\{x,d\},\{a,b\},\{b,c\}\} \subset E(G) \wedge \{\{x,b\},\{x,c\},\{a,c\},\{a,d\},\{b,d\},\{c,d\}\} \cap E(G) = \emptyset\}$$

$$P_{17}(x,a,b,c,d) = \{\{x,a,b,c,d\} \subset V(G) | \{\{x,a\},\{x,c\},\{a,b\},\{c,d\}\} \subset E(G) \wedge \{\{x,b\},\{x,d\},\{a,c\},\{a,d\},\{b,c\},\{b,d\}\} \cap E(G) = \emptyset\}$$

$$P_{18}(x,a,b,c,d) = \{\{x,a,b,c,d\} \subset V(G) | \{\{x,a\},\{a,b\},\{b,c\},\{b,d\}\} \subset E(G) \wedge \{\{x,b\},\{x,c\},\{x,d\},\{a,c\},\{a,d\},\{c,d\}\} \cap E(G) = \emptyset\}$$

$$P_{19}(x,a,b,c,d) = \{\{x,a,b,c,d\} \subset V(G) | \{\{x,a\},\{a,b\},\{a,d\},\{b,c\}\} \subset E(G) \wedge \{\{x,b\},\{x,c\},\{x,d\},\{a,c\},\{b,d\},\{c,d\}\} \cap E(G) = \emptyset\}$$

$$P_{20}(x,a,b,c,d) = \{\{x,a,b,c,d\} \subset V(G) | \{\{x,a\},\{x,d\},\{a,b\},\{a,c\}\} \subset E(G) \wedge \{\{x,b\},\{x,c\},\{a,d\},\{b,c\},\{b,d\},\{c,d\}\} \cap E(G) = \emptyset\}$$

$$P_{21}(x,a,b,c,d) = \{\{x,a,b,c,d\} \subset V(G) | \{\{x,a\},\{x,c\},\{x,d\},\{a,b\}\} \subset E(G) \wedge \{\{x,b\},\{a,c\},\{a,d\},\{b,c\},\{b,d\},\{c,d\}\} \cap E(G) = \emptyset\}$$

$$P_{22}(x,a,b,c,d) = \{\{x,a,b,c,d\} \subset V(G) | \{\{x,a\},\{a,b\},\{a,c\},\{a,d\}\} \subset E(G) \wedge \{\{x,b\},\{x,c\},\{x,d\},\{b,c\},\{b,d\},\{c,d\}\} \cap E(G) = \emptyset\}$$

$$P_{23}(x,a,b,c,d) = \{\{x,a,b,c,d\} \subset V(G) | \{\{x,a\},\{x,b\},\{x,c\},\{x,d\}\} \subset E(G) \wedge \{\{a,b\},\{a,c\},\{a,d\},\{b,c\},\{b,d\},\{c,d\}\} \cap E(G) = \emptyset\}$$

$$P_{24}(x,a,b,c,d) = \{\{x,a,b,c,d\} \subset V(G)| \\ \{\{x,a\},\{a,b\},\{a,d\},\{b,c\},\{b,d\}\} \subset E(G)\wedge \\ \{\{x,b\},\{x,c\},\{x,d\},\{a,c\},\{c,d\}\} \cap E(G) = \emptyset\}$$

$$P_{25}(x,a,b,c,d) = \{\{x,a,b,c,d\} \subset V(G)| \\ \{\{x,a\},\{x,c\},\{a,b\},\{a,c\},\{c,d\}\} \subset E(G)\wedge \\ \{\{x,b\},\{x,d\},\{a,d\},\{b,c\},\{b,d\}\} \cap E(G) = \emptyset\}$$

$$P_{26}(x,a,b,c,d) = \{\{x,a,b,c,d\} \subset V(G)| \\ \{\{x,a\},\{x,c\},\{x,d\},\{a,b\},\{a,d\}\} \subset E(G)\wedge \\ \{\{x,b\},\{a,c\},\{b,c\},\{b,d\},\{c,d\}\} \cap E(G) = \emptyset\}$$

$$P_{27}(x,a,b,c,d) = \{\{x,a,b,c,d\} \subset V(G)| \\ \{\{x,a\},\{a,b\},\{b,c\},\{b,d\},\{c,d\}\} \subset E(G)\wedge \\ \{\{x,b\},\{x,c\},\{x,d\},\{a,c\},\{a,d\}\} \cap E(G) = \emptyset\}$$

$$P_{28}(x,a,b,c,d) = \{\{x,a,b,c,d\} \subset V(G)| \\ \{\{x,a\},\{x,c\},\{a,b\},\{a,d\},\{b,d\}\} \subset E(G)\wedge \\ \{\{x,b\},\{x,d\},\{a,c\},\{b,c\},\{c,d\}\} \cap E(G) = \emptyset\}$$

$$P_{29}(x,a,b,c,d) = \{\{x,a,b,c,d\} \subset V(G)| \\ \{\{x,a\},\{x,d\},\{a,b\},\{a,d\},\{b,c\}\} \subset E(G)\wedge \\ \{\{x,b\},\{x,c\},\{a,c\},\{b,d\},\{c,d\}\} \cap E(G) = \emptyset\}$$

$$P_{30}(x,a,b,c,d) = \{\{x,a,b,c,d\} \subset V(G)| \\ \{\{x,a\},\{x,c\},\{x,d\},\{a,b\},\{c,d\}\} \subset E(G)\wedge \\ \{\{x,b\},\{a,c\},\{a,d\},\{b,c\},\{b,d\}\} \cap E(G) = \emptyset\}$$

$$P_{31}(x,a,b,c,d) = \{\{x,a,b,c,d\} \subset V(G)| \\ \{\{x,a\},\{a,b\},\{a,c\},\{a,d\},\{b,d\}\} \subset E(G)\wedge \\ \{\{x,b\},\{x,c\},\{x,d\},\{b,c\},\{c,d\}\} \cap E(G) = \emptyset\}$$

$$P_{32}(x,a,b,c,d) = \{\{x,a,b,c,d\} \subset V(G)| \\ \{\{x,a\},\{x,d\},\{a,b\},\{a,c\},\{a,d\}\} \subset E(G)\wedge \\ \{\{x,b\},\{x,c\},\{b,c\},\{b,d\},\{c,d\}\} \cap E(G) = \emptyset\}$$

$$P_{33}(x,a,b,c,d) = \{\{x,a,b,c,d\} \subset V(G)| \\ \{\{x,a\},\{x,b\},\{x,c\},\{x,d\},\{a,d\}\} \subset E(G)\wedge \\ \{\{a,b\},\{a,c\},\{b,c\},\{b,d\},\{c,d\}\} \cap E(G) = \emptyset\}$$

$$P_{34}(x,a,b,c,d) = \{\{x,a,b,c,d\} \subset V(G)| \\ \{\{x,a\},\{x,d\},\{a,b\},\{b,c\},\{c,d\}\} \subset E(G)\wedge \\ \{\{x,b\},\{x,c\},\{a,c\},\{a,d\},\{b,d\}\} \cap E(G) = \emptyset\}$$

$$P_{35}(x,a,b,c,d) = \{\{x,a,b,c,d\} \subset V(G) | \\ \{\{x,a\},\{a,b\},\{a,d\},\{b,c\},\{c,d\}\} \subset E(G) \wedge \\ \{\{x,b\},\{x,c\},\{x,d\},\{a,c\},\{b,d\}\} \cap E(G) = \emptyset\}$$

$$P_{36}(x,a,b,c,d) = \{\{x,a,b,c,d\} \subset V(G) | \\ \{\{x,a\},\{x,d\},\{a,b\},\{b,c\},\{b,d\}\} \subset E(G) \wedge \\ \{\{x,b\},\{x,c\},\{a,c\},\{a,d\},\{c,d\}\} \cap E(G) = \emptyset\}$$

$$P_{37}(x,a,b,c,d) = \{\{x,a,b,c,d\} \subset V(G) | \\ \{\{x,a\},\{x,d\},\{a,b\},\{a,c\},\{b,d\}\} \subset E(G) \wedge \\ \{\{x,b\},\{x,c\},\{a,d\},\{b,c\},\{c,d\}\} \cap E(G) = \emptyset\}$$

$$P_{38}(x,a,b,c,d) = \{\{x,a,b,c,d\} \subset V(G) | \\ \{\{x,a\},\{x,c\},\{x,d\},\{a,b\},\{b,d\}\} \subset E(G) \wedge \\ \{\{x,b\},\{a,c\},\{a,d\},\{b,c\},\{c,d\}\} \cap E(G) = \emptyset\}$$

$$P_{39}(x,a,b,c,d) = \{\{x,a,b,c,d\} \subset V(G) | \\ \{\{x,a\},\{a,b\},\{a,c\},\{a,d\},\{b,d\},\{c,d\}\} \subset E(G) \wedge \\ \{\{x,b\},\{x,c\},\{x,d\},\{b,c\}\} \cap E(G) = \emptyset\}$$

$$P_{40}(x,a,b,c,d) = \{\{x,a,b,c,d\} \subset V(G) | \\ \{\{x,a\},\{x,d\},\{a,b\},\{a,c\},\{a,d\},\{b,d\}\} \subset E(G) \wedge \\ \{\{x,b\},\{x,c\},\{b,c\},\{c,d\}\} \cap E(G) = \emptyset\}$$

$$P_{41}(x,a,b,c,d) = \{\{x,a,b,c,d\} \subset V(G) | \\ \{\{x,a\},\{x,c\},\{x,d\},\{a,b\},\{a,c\},\{a,d\}\} \subset E(G) \wedge \\ \{\{x,b\},\{b,c\},\{b,d\},\{c,d\}\} \cap E(G) = \emptyset\}$$

$$P_{42}(x,a,b,c,d) = \{\{x,a,b,c,d\} \subset V(G) | \\ \{\{x,a\},\{x,b\},\{x,c\},\{x,d\},\{a,d\},\{b,d\}\} \subset E(G) \wedge \\ \{\{a,b\},\{a,c\},\{b,c\},\{c,d\}\} \cap E(G) = \emptyset\}$$

$$P_{43}(x,a,b,c,d) = \{\{x,a,b,c,d\} \subset V(G) | \\ \{\{x,a\},\{x,d\},\{a,b\},\{a,c\},\{a,d\},\{b,c\}\} \subset E(G) \wedge \\ \{\{x,b\},\{x,c\},\{b,d\},\{c,d\}\} \cap E(G) = \emptyset\}$$

$$P_{44}(x,a,b,c,d) = \{\{x,a,b,c,d\} \subset V(G) | \\ \{\{x,a\},\{x,b\},\{x,c\},\{x,d\},\{a,c\},\{b,d\}\} \subset E(G) \wedge \\ \{\{a,b\},\{a,d\},\{b,c\},\{c,d\}\} \cap E(G) = \emptyset\}$$

$$P_{45}(x,a,b,c,d) = \{\{x,a,b,c,d\} \subset V(G) | \\ \{\{x,a\},\{a,b\},\{a,d\},\{b,c\},\{b,d\},\{c,d\}\} \subset E(G) \wedge \\ \{\{x,b\},\{x,c\},\{x,d\},\{a,c\}\} \cap E(G) = \emptyset\}$$

$$P_{46}(x,a,b,c,d) = \{\{x,a,b,c,d\} \subset V(G)| \\ \{\{x,a\},\{x,d\},\{a,b\},\{a,d\},\{b,c\},\{b,d\}\} \subset E(G) \wedge \\ \{\{x,b\},\{x,c\},\{a,c\},\{c,d\}\} \cap E(G) = \emptyset\}$$

$$P_{47}(x,a,b,c,d) = \{\{x,a,b,c,d\} \subset V(G)| \\ \{\{x,a\},\{x,c\},\{x,d\},\{a,b\},\{a,d\},\{b,d\}\} \subset E(G) \wedge \\ \{\{x,b\},\{a,c\},\{b,c\},\{c,d\}\} \cap E(G) = \emptyset\}$$

$$P_{48}(x,a,b,c,d) = \{\{x,a,b,c,d\} \subset V(G)| \\ \{\{x,a\},\{x,c\},\{x,d\},\{a,b\},\{a,d\},\{c,d\}\} \subset E(G) \wedge \\ \{\{x,b\},\{a,c\},\{b,c\},\{b,d\}\} \cap E(G) = \emptyset\}$$

$$P_{49}(x,a,b,c,d) = \{\{x,a,b,c,d\} \subset V(G)| \\ \{\{x,a\},\{x,d\},\{a,b\},\{a,c\},\{b,d\},\{c,d\}\} \subset E(G) \wedge \\ \{\{x,b\},\{x,c\},\{a,d\},\{b,c\}\} \cap E(G) = \emptyset\}$$

$$P_{50}(x,a,b,c,d) = \{\{x,a,b,c,d\} \subset V(G)| \\ \{\{x,a\},\{x,c\},\{x,d\},\{a,b\},\{b,c\},\{b,d\}\} \subset E(G) \wedge \\ \{\{x,b\},\{a,c\},\{a,d\},\{c,d\}\} \cap E(G) = \emptyset\}$$

$$P_{51}(x,a,b,c,d) = \{\{x,a,b,c,d\} \subset V(G)| \\ \{\{x,a\},\{x,d\},\{a,b\},\{b,c\},\{b,d\},\{c,d\}\} \subset E(G) \wedge \\ \{\{x,b\},\{x,c\},\{a,c\},\{a,d\}\} \cap E(G) = \emptyset\}$$

$$P_{52}(x,a,b,c,d) = \{\{x,a,b,c,d\} \subset V(G)| \\ \{\{x,a\},\{x,d\},\{a,b\},\{a,d\},\{b,c\},\{c,d\}\} \subset E(G) \wedge \\ \{\{x,b\},\{x,c\},\{a,c\},\{b,d\}\} \cap E(G) = \emptyset\}$$

$$P_{53}(x,a,b,c,d) = \{\{x,a,b,c,d\} \subset V(G)| \\ \{\{x,a\},\{x,c\},\{x,d\},\{a,b\},\{b,d\},\{c,d\}\} \subset E(G) \wedge \\ \{\{x,b\},\{a,c\},\{a,d\},\{b,c\}\} \cap E(G) = \emptyset\}$$

$$P_{54}(x,a,b,c,d) = \{\{x,a,b,c,d\} \subset V(G)| \\ \{\{x,a\},\{x,d\},\{a,b\},\{a,c\},\{a,d\},\{b,d\},\{c,d\}\} \subset E(G) \wedge \\ \{\{x,b\},\{x,c\},\{b,c\}\} \cap E(G) = \emptyset\}$$

$$P_{55}(x,a,b,c,d) = \{\{x,a,b,c,d\} \subset V(G)| \\ \{\{x,a\},\{x,b\},\{x,c\},\{x,d\},\{a,d\},\{b,d\},\{c,d\}\} \subset E(G) \wedge \\ \{\{a,b\},\{a,c\},\{b,c\}\} \cap E(G) = \emptyset\}$$

$$P_{56}(x,a,b,c,d) = \{\{x,a,b,c,d\} \subset V(G)| \\ \{\{x,a\},\{a,b\},\{a,c\},\{a,d\},\{b,c\},\{b,d\},\{c,d\}\} \subset E(G) \wedge \\ \{\{x,b\},\{x,c\},\{x,d\}\} \cap E(G) = \emptyset\}$$

$$P_{57}(x,a,b,c,d) = \{\{x,a,b,c,d\} \subset V(G)| \\ \{\{x,a\},\{x,c\},\{x,d\},\{a,b\},\{a,c\},\{a,d\},\{c,d\}\} \subset E(G) \wedge \\ \{\{x,b\},\{b,c\},\{b,d\}\} \cap E(G) = \emptyset\}$$

$$P_{58}(x,a,b,c,d) = \{\{x,a,b,c,d\} \subset V(G)| \\ \{\{x,a\},\{x,b\},\{x,c\},\{x,d\},\{a,c\},\{a,d\},\{c,d\}\} \subset E(G) \wedge \\ \{\{a,b\},\{b,c\},\{b,d\}\} \cap E(G) = \emptyset\}$$

$$P_{59}(x,a,b,c,d) = \{\{x,a,b,c,d\} \subset V(G)| \\ \{\{x,a\},\{x,d\},\{a,b\},\{a,d\},\{b,c\},\{b,d\},\{c,d\}\} \subset E(G) \wedge \\ \{\{x,b\},\{x,c\},\{a,c\}\} \cap E(G) = \emptyset\}$$

$$P_{60}(x,a,b,c,d) = \{\{x,a,b,c,d\} \subset V(G)| \\ \{\{x,a\},\{x,c\},\{x,d\},\{a,b\},\{a,d\},\{b,d\},\{c,d\}\} \subset E(G) \wedge \\ \{\{x,b\},\{a,c\},\{b,c\}\} \cap E(G) = \emptyset\}$$

$$P_{61}(x,a,b,c,d) = \{\{x,a,b,c,d\} \subset V(G)| \\ \{\{x,a\},\{x,b\},\{x,c\},\{x,d\},\{a,c\},\{a,d\},\{b,d\}\} \subset E(G) \wedge \\ \{\{a,b\},\{b,c\},\{c,d\}\} \cap E(G) = \emptyset\}$$

$$P_{62}(x,a,b,c,d) = \{\{x,a,b,c,d\} \subset V(G)| \\ \{\{x,a\},\{x,d\},\{a,b\},\{a,c\},\{b,c\},\{b,d\},\{c,d\}\} \subset E(G) \wedge \\ \{\{x,b\},\{x,c\},\{a,d\}\} \cap E(G) = \emptyset\}$$

$$P_{63}(x,a,b,c,d) = \{\{x,a,b,c,d\} \subset V(G)| \\ \{\{x,a\},\{x,c\},\{x,d\},\{a,b\},\{a,d\},\{b,c\},\{b,d\}\} \subset E(G) \wedge \\ \{\{x,b\},\{a,c\},\{c,d\}\} \cap E(G) = \emptyset\}$$

$$P_{64}(x,a,b,c,d) = \{\{x,a,b,c,d\} \subset V(G)| \\ \{\{x,a\},\{x,c\},\{x,d\},\{a,b\},\{a,d\},\{b,c\},\{c,d\}\} \subset E(G) \wedge \\ \{\{x,b\},\{a,c\},\{b,d\}\} \cap E(G) = \emptyset\}$$

$$P_{65}(x,a,b,c,d) = \{\{x,a,b,c,d\} \subset V(G)|\{\{x,a\},\{x,d\},\{a,b\},\{a,c\},\{a,d\}, \\ \{b,c\},\{b,d\},\{c,d\}\} \subset E(G) \wedge \\ \{\{x,b\},\{x,c\}\} \cap E(G) = \emptyset\}$$

$$P_{66}(x,a,b,c,d) = \{\{x,a,b,c,d\} \subset V(G)|\{\{x,a\},\{x,c\},\{x,d\},\{a,b\},\{a,c\}, \\ \{a,d\},\{b,d\},\{c,d\}\} \subset E(G) \wedge \\ \{\{x,b\},\{b,c\}\} \cap E(G) = \emptyset\}$$

$$P_{67}(x,a,b,c,d) = \{\{x,a,b,c,d\} \subset V(G)|\{\{x,a\},\{x,b\},\{x,c\},\{x,d\},\{a,c\}, \\ \{a,d\},\{b,d\},\{c,d\}\} \subset E(G) \wedge \\ \{\{a,b\},\{b,c\}\} \cap E(G) = \emptyset\}$$

$$\begin{aligned}P_{68}(x,a,b,c,d) = \{\{x,a,b,c,d\} \subset V(G) | \{\{x,a\},\{x,c\},\{x,d\},\{a,b\},\{a,d\},\\ \{b,c\},\{b,d\},\{c,d\}\} \subset E(G) \wedge \\ \{\{x,b\},\{a,c\}\} \cap E(G) = \emptyset\}\end{aligned}$$

$$\begin{aligned}P_{69}(x,a,b,c,d) = \{\{x,a,b,c,d\} \subset V(G) | \{\{x,a\},\{x,b\},\{x,c\},\{x,d\},\{a,c\},\\ \{a,d\},\{b,c\},\{b,d\}\} \subset E(G) \wedge \\ \{\{a,b\},\{c,d\}\} \cap E(G) = \emptyset\}\end{aligned}$$

$$\begin{aligned}P_{70}(x,a,b,c,d) = \{\{x,a,b,c,d\} \subset V(G) | \{\{x,a\},\{x,c\},\{x,d\},\{a,b\},\{a,c\},\\ \{a,d\},\{b,c\},\{b,d\},\{c,d\}\} \subset E(G) \wedge \\ \{\{x,b\}\} \cap E(G) = \emptyset\}\end{aligned}$$

$$\begin{aligned}P_{71}(x,a,b,c,d) = \{\{x,a,b,c,d\} \subset V(G) | \{\{x,a\},\{x,b\},\{x,c\},\{x,d\},\{a,c\},\\ \{a,d\},\{b,c\},\{b,d\},\{c,d\}\} \subset E(G) \wedge \\ \{\{a,b\}\} \cap E(G) = \emptyset\}\end{aligned}$$

$$\begin{aligned}P_{72}(x,a,b,c,d) = \{\{x,a,b,c,d\} \subset V(G) | \{\{x,a\},\{x,b\},\{x,c\},\{x,d\},\{a,b\},\{a,c\},\\ \{a,d\},\{b,c\},\{b,d\},\{c,d\}\} \subset E(G)\}\end{aligned}$$

$$4o_{73} + 2o_{79} + o_{80} + o_{98} + o_{99} + o_{158} + o_{159} + 2o_{287} = \sum_{P_{22}(x,a,b,c,d)} (c(a) - 4)$$

$$5o_{74} + 2o_{81} + o_{101} + o_{161} + o_{288} = \sum_{P_{23}(x,a,b,c,d)} (c(x) - 4)$$

$$\begin{aligned}2o_{75} + o_{89} + 2o_{94} + 2o_{106} + 2o_{125} + o_{127}\\ + o_{142} + o_{144} + o_{169} + o_{170} + o_{195} + \\ o_{196} + o_{221} + 2o_{226} + 2o_{300} + o_{308} = \sum_{P_{19}(x,a,b,c,d)} (c(a) - 3)\end{aligned}$$

$$\begin{aligned}3o_{76} + o_{90} + 2o_{96} + 2o_{107} + 2o_{126} + o_{143} + o_{172}\\ + o_{197} + o_{220} + 2o_{225} + o_{301} + o_{309} = \sum_{P_{18}(x,a,b,c,d)} (c(b) - 3)\end{aligned}$$

$$o_{77} + o_{80} + o_{95} + o_{99} + o_{155} + o_{159} + 2o_{285} + 2o_{287} = \sum_{P_{22}(x,a,b,c,d)} (c(x) - 1)$$

$$\begin{aligned}o_{78} + 2o_{81} + 2o_{97} + 2o_{101} + 3o_{157} + 3o_{161} + 4o_{286} + 4o_{288} = \\ \sum_{P_{23}(x,a,b,c,d)} (c(a) - 1) + (c(b) - 1) + (c(c) - 1) + (c(d) - 1)\end{aligned}$$

$$2o_{79} + 2o_{98} + o_{99} + 3o_{158} + 2o_{159} + 6o_{287} = \sum_{P_{22}(x,a,b,c,d)} c(a,b) + c(a,c) + c(a,d)$$

$$o_{80} + o_{99} + o_{159} + 2o_{287} = \sum_{P_{22}(x,a,b,c,d)} c(x,a)$$

$$2o_{81} + 2o_{101} + 3o_{161} + 4o_{288} = \sum_{P_{23}(x,a,b,c,d)} c(x,a) + c(x,b) + c(x,c) + c(x,d)$$

$$\begin{aligned} 2o_{82} + o_{89} + 2o_{102} + 2o_{106} + o_{133} + o_{134} \\ +o_{142} + o_{144} + o_{189} + o_{190} + o_{195} \\ +o_{196} + 2o_{217} + 2o_{226} + o_{303} + o_{308} = \sum_{P_{19}(x,a,b,c,d)} (c(b) - 2) \end{aligned}$$

$$\begin{aligned} 2o_{83} + o_{93} + 2o_{105} + 2o_{109} + o_{135} + o_{147} + o_{194} \\ +o_{200} + o_{218} + o_{227} + o_{306} + o_{311} = \sum_{P_{21}(x,a,b,c,d)} (c(a) - 2) \end{aligned}$$

$$\begin{aligned} o_{84} + 2o_{94} + 2o_{102} + 2o_{106} + 2o_{165} + o_{166} \\ +o_{169} + o_{170} + o_{189} + o_{190} + o_{195} \\ +o_{196} + 2o_{297} + 2o_{300} + o_{303} + o_{308} = \sum_{P_{19}(x,a,b,c,d)} (c(c) - 1) \end{aligned}$$

$$\begin{aligned} 2o_{85} + 2o_{113} + o_{137} + 2o_{148} + o_{167} + 2o_{179} \\ +o_{189} + o_{191} + o_{230} + 2o_{238} + o_{243} \\ +o_{244} + o_{321} + o_{324} + o_{333} + o_{338} = \sum_{P_{15}(x,a,b,c,d)} (c(c) - 2) \end{aligned}$$

$$\begin{aligned} o_{86} + o_{95} + 2o_{104} + o_{108} + o_{166} + o_{171} + o_{192} \\ +o_{198} + o_{298} + o_{301} + o_{305} + o_{310} = \sum_{P_{20}(x,a,b,c,d)} (c(c) - 1) \end{aligned}$$

$$\begin{aligned} o_{87} + 2o_{96} + o_{103} + 2o_{107} + o_{167} + o_{172} + o_{191} \\ +o_{197} + o_{298} + o_{301} + o_{304} + o_{309} = \sum_{P_{18}(x,a,b,c,d)} (c(x) - 1) \end{aligned}$$

$$\begin{aligned} o_{88} + 2o_{97} + 2o_{105} + 2o_{109} + 2o_{168} + o_{174} + o_{194} + \\ o_{200} + o_{299} + o_{302} + o_{306} + o_{311} = \sum_{P_{21}(x,a,b,c,d)} (c(b) - 1) \end{aligned}$$

$$o_{89} + 2o_{106} + o_{142} + o_{144} + o_{195} + o_{196} + 2o_{226} + o_{308} = \sum_{P_{19}(x,a,b,c,d)} c(a,b)$$

$$o_{90} + 2o_{107} + o_{143} + o_{197} + 2o_{225} + o_{309} = \sum_{P_{18}(x,a,b,c,d)} c(a,b)$$

$$\begin{aligned} 2o_{91} + 2o_{117} + o_{145} + 2o_{152} + o_{170} + 2o_{185} \\ +2o_{196} + 2o_{235} + o_{245} + 4o_{251} \\ +o_{324} + 2o_{328} + o_{348} + 2o_{353} = \sum_{P_{25}(x,a,b,c,d)} (c(a) - 3) + (c(c) - 3) \end{aligned}$$

$$o_{92} + o_{108} + o_{146} + o_{198} + 2o_{228} + o_{310} = \sum_{P_{20}(x,a,b,c,d)} c(x,a)$$

$$o_{93} + 2o_{109} + o_{147} + o_{200} + o_{227} + o_{311} = \sum_{P_{21}(x,a,b,c,d)} c(x,a)$$

$$2o_{94} + 2o_{106} + o_{169} + o_{170} + o_{195} + o_{196} + 2o_{300} + o_{308} = \sum_{P_{19}(x,a,b,c,d)} (c(a,c) - 1)$$

$$\begin{aligned} o_{95} + o_{99} + 2o_{155} + 2o_{159} \\ +6o_{285} + 6o_{287} = \sum_{P_{22}(x,a,b,c,d)} (c(x,b) - 1) + (c(x,c) - 1) + (c(x,d) - 1) \end{aligned}$$

$$2o_{96} + 2o_{107} + o_{172} + o_{197} + o_{301} + o_{309} = \sum_{P_{18}(x,a,b,c,d)} (c(x,b) - 1)$$

$$\begin{aligned} o_{97} + o_{101} + 3o_{157} + 3o_{161} + 6o_{286} + 6o_{288} = \\ \sum_{P_{23}(x,a,b,c,d)} (c(a,b) - 1) + (c(a,c) - 1) \\ + (c(b,c) - 1) + (c(a,d) - 1) \\ + (c(b,d) - 1) + (c(c,d) - 1) \end{aligned}$$

$$o_{98} + 3o_{158} + o_{159} + 6o_{287} = \sum_{P_{22}(x,a,b,c,d)} c(a,b,c) + c(a,b,d) + c(a,c,d)$$

$$o_{99} + 2o_{159} + 6o_{287} = \sum_{P_{22}(x,a,b,c,d)} c(x,a,b) + c(x,a,c) + c(x,a,d)$$

$$\begin{aligned} 2o_{100} + 3o_{160} + o_{177} + 2o_{209} + o_{255} + o_{268} + 2o_{295} \\ +o_{311} + o_{340} + o_{354} + o_{369} + 2o_{378} = \sum_{P_{41}(x,a,b,c,d)} (c(a) - 4) \end{aligned}$$

$$\begin{aligned} o_{101} + 3o_{161} + 6o_{288} = \sum_{P_{23}(x,a,b,c,d)} c(x,a,b) + c(x,a,c) + c(x,b,c) \\ + c(x,a,d) + c(x,b,d) + c(x,c,d) \end{aligned}$$

$$2o_{102} + 2o_{106} + o_{189} + o_{190} + o_{195} + o_{196} + o_{303} + o_{308} = \sum_{P_{19}(x,a,b,c,d)} c(b,c)$$

$$o_{103} + 2o_{107} + o_{191} + o_{197} + o_{304} + o_{309} = \sum_{P_{18}(x,a,b,c,d)} c(x,a)$$

$$2o_{104} + o_{108} + o_{192} + o_{198} + o_{305} + o_{310} = \sum_{P_{20}(x,a,b,c,d)} c(x,c)$$

$$2o_{105} + 2o_{109} + o_{194} + o_{200} + o_{306} + o_{311} = \sum_{P_{21}(x,a,b,c,d)} c(a,b)$$

$$2o_{106} + o_{195} + o_{196} + o_{308} = \sum_{P_{19}(x,a,b,c,d)} c(a,b,c)$$

$$2o_{107} + o_{197} + o_{309} = \sum_{P_{18}(x,a,b,c,d)} c(x,a,b)$$

$$o_{108} + o_{198} + o_{310} = \sum_{P_{20}(x,a,b,c,d)} c(x,a,c)$$

$$2o_{109} + o_{200} + o_{311} = \sum_{P_{21}(x,a,b,c,d)} c(x,a,b)$$

$$3o_{110} + o_{206} + o_{207} + 2o_{316} = \sum_{P_{31}(x,a,b,c,d)} c(a,b,d)$$

$$2o_{111} + o_{208} + 2o_{317} = \sum_{P_{32}(x,a,b,c,d)} c(x,a,d)$$

$$3o_{112} + o_{210} + o_{318} = \sum_{P_{33}(x,a,b,c,d)} c(x,a,d)$$

$$2o_{113} + 2o_{148} + o_{189} + o_{191} + o_{243} + o_{244} + o_{324} + o_{338} = \sum_{P_{15}(x,a,b,c,d)} (c(a,c) - 1)$$

$$o_{114} + o_{149} + o_{190} + o_{245} + o_{325} + o_{339} = \sum_{P_{17}(x,a,b,c,d)} (c(a,c) - 1)$$

$$2o_{115} + 2o_{150} + o_{193} + o_{194} + o_{246} + o_{248} + o_{326} + o_{340} = \sum_{P_{16}(x,a,b,c,d)} (c(x,b) - 1)$$

$$\begin{aligned} &o_{116} + 3o_{158} + o_{195} + 2o_{206} + o_{261} + 2o_{266} + o_{301} \\ &\quad + o_{309} + o_{347} + o_{352} + 2o_{372} + 2o_{376} = \sum_{P_{39}(x,a,b,c,d)} (c(d) - 3) \end{aligned}$$

$$2o_{117} + 2o_{159} + o_{196} + o_{198} + 2o_{207} + o_{208} + o_{262} + 2o_{267} + 2o_{300} + o_{308} + o_{346} + o_{348} + o_{352} + 2o_{353} + 2o_{373} + 2o_{377} = \sum_{P_{40}(x,a,b,c,d)} (c(d) - 3)$$

$$o_{118} + 3o_{160} + o_{199} + 2o_{209} + o_{264} + o_{268} + o_{302} + o_{311} + o_{350} + o_{354} + 2o_{374} + 2o_{378} = \sum_{P_{41}(x,a,b,c,d)} (c(x) - 3)$$

$$2o_{119} + 2o_{125} + o_{133} + o_{142} + 2o_{165} + o_{169} + o_{189} + o_{195} + o_{212} + 2o_{217} + o_{221} + 2o_{226} + 2o_{297} + 2o_{300} + o_{303} + o_{308} = \sum_{P_{19}(x,a,b,c,d)} (c(d) - 1)$$

$$o_{120} + 2o_{126} + o_{133} + o_{143} + o_{167} + o_{172} + o_{191} + o_{197} + 2o_{211} + 4o_{216} + 2o_{220} + 4o_{225} + 2o_{298} + 2o_{301} + 2o_{304} + 2o_{309} = \sum_{P_{18}(x,a,b,c,d)} (c(c) - 1) + (c(d) - 1)$$

$$o_{121} + o_{127} + o_{134} + o_{144} + o_{166} + o_{170} + o_{190} + o_{196} + o_{212} + 2o_{217} + o_{221} + 2o_{226} + 2o_{297} + 2o_{300} + o_{303} + o_{308} = \sum_{P_{19}(x,a,b,c,d)} (c(x) - 1)$$

$$2o_{122} + 2o_{129} + o_{135} + o_{147} + 2o_{168} + o_{174} + o_{194} + o_{200} + 2o_{214} + 2o_{218} + 2o_{223} + 2o_{227} + 2o_{299} + 2o_{302} + 2o_{306} + 2o_{311} = \sum_{P_{21}(x,a,b,c,d)} (c(c) - 1) + (c(d) - 1)$$

$$2o_{123} + o_{142} + o_{143} + 2o_{151} + 2o_{229} + o_{230} + 2o_{233} + 2o_{234} + o_{243} + o_{244} + 2o_{249} + 2o_{250} + o_{342} + o_{346} + o_{347} + o_{352} = \sum_{P_{24}(x,a,b,c,d)} (c(d) - 2)$$

$$2o_{124} + o_{146} + o_{147} + 2o_{153} + o_{231} + 2o_{232} + o_{236} + 2o_{237} + o_{247} + o_{248} + o_{252} + 2o_{253} + o_{344} + o_{349} + o_{350} + o_{354} = \sum_{P_{26}(x,a,b,c,d)} (c(d) - 2)$$

$$2o_{125} + o_{142} + o_{169} + o_{195} + o_{221} + 2o_{226} + 2o_{300} + o_{308} = \sum_{P_{19}(x,a,b,c,d)} c(a, d)$$

$$2o_{126}+o_{143}+o_{172}+o_{197}+2o_{220}+4o_{225}+2o_{301}+2o_{309}=\sum_{P_{18}(x,a,b,c,d)} c(b,c)+c(b,d)$$

$$o_{127}+o_{144}+o_{170}+o_{196}+o_{221}+2o_{226}+2o_{300}+o_{308}=\sum_{P_{19}(x,a,b,c,d)} c(x,a)$$

$$2o_{128}+o_{146}+o_{171}+o_{198}+2o_{224}+4o_{228}+2o_{301}+2o_{310}=\sum_{P_{20}(x,a,b,c,d)} c(a,b)+c(a,d)$$

$$2o_{129}+o_{147}+o_{174}+o_{200}+2o_{223}+2o_{227}+2o_{302}+2o_{311}=\sum_{P_{21}(x,a,b,c,d)} c(x,c)+c(x,d)$$

$$\begin{aligned} &o_{130}+2o_{165}+2o_{179}+o_{189}+2o_{211}+2o_{229}\\ &+2o_{238}+o_{243}+2o_{319}+2o_{320}+o_{321}\\ &+o_{324}+o_{330}+o_{332}+o_{333}+o_{338}=\sum_{P_{15}(x,a,b,c,d)}(c(d)-1)\end{aligned}$$

$$\begin{aligned} &o_{131}+o_{166}+2o_{180}+o_{190}+2o_{214}+o_{231}\\ &+2o_{240}+o_{245}+2o_{319}+2o_{321}+2o_{325}\\ &+2o_{331}+2o_{334}+2o_{339}=\sum_{P_{17}(x,a,b,c,d)}(c(b)-1)+(c(d)-1)\end{aligned}$$

$$\begin{aligned} &o_{132}+o_{167}+o_{181}+o_{191}+2o_{213}+o_{230}\\ &+2o_{239}+o_{244}+2o_{319}+2o_{320}+o_{321}\\ &+o_{324}+o_{330}+o_{332}+o_{333}+o_{338}=\sum_{P_{15}(x,a,b,c,d)}(c(x)-1)\end{aligned}$$

$$o_{133}+o_{142}+o_{189}+o_{195}+2o_{217}+2o_{226}+o_{303}+o_{308}=\sum_{P_{19}(x,a,b,c,d)}(c(b,d)-1)$$

$$o_{134}+o_{144}+o_{190}+o_{196}+2o_{217}+2o_{226}+o_{303}+o_{308}=\sum_{P_{19}(x,a,b,c,d)}(c(x,b)-1)$$

$$\begin{aligned} &o_{135}+o_{147}+o_{194}+o_{200}+2o_{218}\\ &+2o_{227}+2o_{306}+2o_{311}=\sum_{P_{21}(x,a,b,c,d)}(c(a,c)-1)+(c(a,d)-1)\end{aligned}$$

$$o_{136}+2o_{148}+2o_{229}+2o_{239}+o_{243}+o_{244}+o_{332}+o_{338}=\sum_{P_{15}(x,a,b,c,d)} c(a,b)$$

$$o_{137}+2o_{148}+o_{230}+2o_{238}+o_{243}+o_{244}+o_{333}+o_{338}=\sum_{P_{15}(x,a,b,c,d)} c(b,c)$$

$$\begin{aligned}o_{138} + o_{170} + 2o_{185} + o_{196} + o_{231} + 2o_{235} \\ +o_{245} + 2o_{251} + 2o_{320} + 2o_{324} \\ +2o_{328} + 2o_{343} + 2o_{348} + 2o_{353} &= \sum_{P_{25}(x,a,b,c,d)} (c(b)-1) + (c(d)-1)\end{aligned}$$

$$o_{139} + 2o_{149} + o_{231} + 2o_{240} + 2o_{245} + o_{334} + 2o_{339} = \sum_{P_{17}(x,a,b,c,d)} c(x,a) + c(x,c)$$

$$o_{140} + 2o_{150} + o_{230} + 2o_{242} + o_{246} + o_{248} + o_{335} + o_{340} = \sum_{P_{16}(x,a,b,c,d)} c(a,b)$$

$$o_{141} + 2o_{150} + 2o_{232} + o_{241} + o_{246} + o_{248} + o_{336} + o_{340} = \sum_{P_{16}(x,a,b,c,d)} c(x,a)$$

$$o_{142} + o_{195} + 2o_{226} + o_{308} = \sum_{P_{19}(x,a,b,c,d)} c(a,b,d)$$

$$o_{143} + o_{197} + 4o_{225} + 2o_{309} = \sum_{P_{18}(x,a,b,c,d)} c(a,b,c) + c(a,b,d)$$

$$o_{144} + o_{196} + 2o_{226} + o_{308} = \sum_{P_{19}(x,a,b,c,d)} c(x,a,b)$$

$$\begin{aligned}o_{145} + o_{177} + o_{199} + 2o_{209} + 2o_{235} + 2o_{255} \\ +2o_{264} + 2o_{268} + o_{336} + o_{340} + o_{350} \\ +o_{354} + 2o_{364} + 2o_{369} + 4o_{374} + 4o_{378} &= \sum_{P_{41}(x,a,b,c,d)} (c(c)-2) + (c(d)-2)\end{aligned}$$

$$o_{146} + o_{198} + 4o_{228} + 2o_{310} = \sum_{P_{20}(x,a,b,c,d)} c(x,a,b) + c(x,a,d)$$

$$o_{147} + o_{200} + 2o_{227} + 2o_{311} = \sum_{P_{21}(x,a,b,c,d)} c(x,a,c) + c(x,a,d)$$

$$2o_{148} + o_{243} + o_{244} + o_{338} = \sum_{P_{15}(x,a,b,c,d)} c(a,b,c)$$

$$o_{149} + o_{245} + o_{339} = \sum_{P_{17}(x,a,b,c,d)} c(x,a,c)$$

$$2o_{150} + o_{246} + o_{248} + o_{340} = \sum_{P_{16}(x,a,b,c,d)} c(x,a,b)$$

$$2o_{151} + 2o_{249} + 2o_{250} + o_{352} = \sum_{P_{24}(x,a,b,c,d)} c(a,b,d)$$

$$o_{152} + 2o_{251} + o_{353} = \sum_{P_{25}(x,a,b,c,d)} c(x,a,c)$$

$$2o_{153} + o_{252} + 2o_{253} + o_{354} = \sum_{P_{26}(x,a,b,c,d)} c(x,a,d)$$

$$o_{154} + o_{158} + 2o_{285} + 2o_{287} = \sum_{P_{22}(x,a,b,c,d)} (c(b,c,d) - 1)$$

$$o_{155} + o_{159} + 6o_{285} + 6o_{287} = \sum_{P_{22}(x,a,b,c,d)} (c(x,b,c) - 1) + (c(x,b,d) - 1) + (c(x,c,d) - 1)$$

$$o_{156} + 4o_{286} + o_{306} + 2o_{315} + o_{365} + o_{375} + 2o_{438} + o_{441} = \sum_{P_{50}(x,a,b,c,d)} (c(b) - 3)$$

$$o_{157} + o_{161} + 4o_{286} + 4o_{288} = \sum_{P_{23}(x,a,b,c,d)} (c(a,b,c) - 1) + (c(a,b,d) - 1) + (c(a,c,d) - 1) + (c(b,c,d) - 1)$$

$$o_{158} + 2o_{287} = \sum_{P_{22}(x,a,b,c,d)} c(a,b,c,d)$$

$$o_{159} + 6o_{287} = \sum_{P_{22}(x,a,b,c,d)} c(x,a,b,c) + c(x,a,b,d) + c(x,a,c,d)$$

$$3o_{160} + 2o_{209} + o_{268} + o_{311} + o_{354} + 2o_{378} = \sum_{P_{41}(x,a,b,c,d)} (c(x,a) - 2)$$

$$o_{161} + 4o_{288} = \sum_{P_{23}(x,a,b,c,d)} c(x,a,b,c) + c(x,a,b,d) + c(x,a,c,d) + c(x,b,c,d)$$

$$4o_{162} + 2o_{175} + o_{206} + o_{293} + o_{308} + 2o_{316} = \sum_{P_{31}(x,a,b,c,d)} c(a,c)$$

$$o_{163} + o_{293} + 2o_{294} + 2o_{295} + o_{338} + o_{340} + 3o_{393} + 2o_{394} + o_{407} + o_{408} + o_{410} + 2o_{447} = \sum_{P_{43}(x,a,b,c,d)} (c(a) - 4)$$

$$o_{164} + 2o_{296} + o_{341} + 3o_{395} + o_{411} + o_{448} = \sum_{P_{44}(x,a,b,c,d)} (c(x) - 4)$$

$$2o_{165} + o_{169} + o_{189} + o_{195} + 2o_{297} + 2o_{300} + o_{303} + o_{308} = \sum_{P_{19}(x,a,b,c,d)} c(c,d)$$

$$o_{166} + o_{170} + o_{190} + o_{196} + 2o_{297} + 2o_{300} + o_{303} + o_{308} = \sum_{P_{19}(x,a,b,c,d)} c(x,c)$$

$$o_{167} + o_{172} + o_{191} + o_{197} + 2o_{298} + 2o_{301} + 2o_{304} + 2o_{309} = \sum_{P_{18}(x,a,b,c,d)} c(x,c) + c(x,d)$$

$$2o_{168} + o_{174} + o_{194} + o_{200} + 2o_{299} + 2o_{302} + 2o_{306} + 2o_{311} = \sum_{P_{21}(x,a,b,c,d)} c(b,c) + c(b,d)$$

$$o_{169} + o_{195} + 2o_{300} + o_{308} = \sum_{P_{19}(x,a,b,c,d)} c(a,c,d)$$

$$o_{170} + o_{196} + 2o_{300} + o_{308} = \sum_{P_{19}(x,a,b,c,d)} c(x,a,c)$$

$$o_{171} + o_{198} + 2o_{301} + 2o_{310} = \sum_{P_{20}(x,a,b,c,d)} c(a,b,c) + c(a,c,d)$$

$$o_{172} + o_{197} + 2o_{301} + 2o_{309} = \sum_{P_{18}(x,a,b,c,d)} c(x,b,c) + c(x,b,d)$$

$$\begin{aligned} &o_{173} + 2o_{302} + 2o_{305} + o_{326} + o_{334} + o_{350} \\ &\quad + o_{351} + o_{363} + 2o_{402} + 2o_{406} + o_{415} \\ &\quad + o_{419} + o_{422} + 2o_{424} + o_{453} + o_{457} = \sum_{P_{53}(x,a,b,c,d)} (c(d) - 3) \end{aligned}$$

$$o_{174} + o_{200} + 2o_{302} + 2o_{311} = \sum_{P_{21}(x,a,b,c,d)} c(x,b,c) + c(x,b,d)$$

$$\begin{aligned} &2o_{175} + 2o_{206} + 8o_{254} + 4o_{266} + o_{338} \\ &\quad + o_{352} + 2o_{367} + 4o_{376} = \sum_{P_{39}(x,a,b,c,d)} (c(a,b) - 1) + (c(a,c) - 1) \end{aligned}$$

$$o_{176} + 2o_{207} + 2o_{255} + 2o_{267} + o_{338} + o_{352} + o_{368} + 2o_{377} = \sum_{P_{40}(x,a,b,c,d)} (c(a,b) - 1)$$

$$\begin{aligned} &o_{177} + 2o_{209} + 2o_{255} + 2o_{268} + o_{340} \\ &\quad + o_{354} + 2o_{369} + 4o_{378} = \sum_{P_{41}(x,a,b,c,d)} (c(a,c) - 1) + (c(a,d) - 1) \end{aligned}$$

$$\begin{aligned} &2o_{178} + 2o_{210} + 8o_{256} + 4o_{269} + 2o_{341} \\ &\quad + 2o_{355} + 2o_{371} + 2o_{379} = \sum_{P_{42}(x,a,b,c,d)} (c(x,a) - 1) + (c(x,b) - 1) \end{aligned}$$

$$2o_{179} + o_{189} + 2o_{238} + o_{243} + o_{321} + o_{324} + o_{333} + o_{338} = \sum_{P_{15}(x,a,b,c,d)} c(c,d)$$

$$2o_{180} + o_{190} + 2o_{240} + o_{245} + o_{321} + 2o_{325} + o_{334} + 2o_{339} = \sum_{P_{17}(x,a,b,c,d)} c(a,b) + c(c,d)$$

$$o_{181} + o_{191} + 2o_{239} + o_{244} + 2o_{320} + o_{324} + o_{332} + o_{338} = \sum_{P_{15}(x,a,b,c,d)} c(x,a)$$

$$2o_{182} + o_{193} + o_{241} + o_{246} + o_{323} + o_{326} + o_{336} + o_{340} = \sum_{P_{16}(x,a,b,c,d)} c(x,d)$$

$$2o_{183} + o_{194} + 2o_{242} + o_{248} + 2o_{322} + o_{326} + o_{335} + o_{340} = \sum_{P_{16}(x,a,b,c,d)} c(b,c)$$

$$o_{184} + o_{243} + 2o_{274} + 2o_{382} + o_{388} + o_{407} = \sum_{P_{27}(x,a,b,c,d)} c(a,b)$$

$$\begin{aligned} &o_{185} + o_{293} + o_{324} + o_{338} + o_{385} + 2o_{394} + o_{399} \\ &\quad + o_{400} + o_{407} + o_{408} + 2o_{445} + 2o_{447} = \sum_{P_{43}(x,a,b,c,d)} (c(d) - 2) \end{aligned}$$

$$\begin{aligned} &o_{186} + 2o_{294} + o_{324} + o_{326} + o_{338} + o_{340} + 2o_{383} \\ &\quad + 6o_{393} + 2o_{399} + o_{400} + 2o_{402} + 2o_{407} \\ &\quad\quad + o_{408} + 2o_{410} + 4o_{445} + 4o_{447} = \sum_{P_{43}(x,a,b,c,d)} (c(b) - 2) + (c(c) - 2) \end{aligned}$$

$$\begin{aligned} &o_{187} + 2o_{296} + 2o_{327} + 2o_{341} + 2o_{384} + 6o_{395} + 3o_{403} + 3o_{411} + 4o_{446} + 4o_{448} \\ &\quad = \sum_{P_{44}(x,a,b,c,d)} (c(a) - 2) + (c(b) - 2) + (c(c) - 2) + (c(d) - 2) \end{aligned}$$

$$\begin{aligned} &o_{188} + 2o_{295} + o_{326} + o_{340} + o_{385} + 2o_{394} + o_{400} \\ &\quad + o_{402} + o_{408} + o_{410} + 2o_{445} + 2o_{447} = \sum_{P_{43}(x,a,b,c,d)} (c(x) - 2) \end{aligned}$$

$$o_{189} + o_{195} + o_{303} + o_{308} = \sum_{P_{19}(x,a,b,c,d)} c(b,c,d)$$

$$o_{190} + o_{196} + o_{303} + o_{308} = \sum_{P_{19}(x,a,b,c,d)} c(x,b,c)$$

$$o_{191} + o_{197} + 2o_{304} + 2o_{309} = \sum_{P_{18}(x,a,b,c,d)} c(x,a,c) + c(x,a,d)$$

$$o_{192} + o_{198} + 2o_{305} + 2o_{310} = \sum_{P_{20}(x,a,b,c,d)} c(x,b,c) + c(x,c,d)$$

$$o_{193} + o_{246} + o_{326} + o_{340} = \sum_{P_{16}(x,a,b,c,d)} c(x,b,d)$$

$$o_{194} + o_{200} + 2o_{306} + 2o_{311} = \sum_{P_{21}(x,a,b,c,d)} c(a,b,c) + c(a,b,d)$$

$$o_{195} + o_{308} = \sum_{P_{19}(x,a,b,c,d)} c(a,b,c,d)$$

$$o_{196} + o_{308} = \sum_{P_{19}(x,a,b,c,d)} c(x,a,b,c)$$

$$o_{197} + 2o_{309} = \sum_{P_{18}(x,a,b,c,d)} c(x,a,b,c) + c(x,a,b,d)$$

$$o_{198} + 2o_{310} = \sum_{P_{20}(x,a,b,c,d)} c(x,a,b,c) + c(x,a,c,d)$$

$$\begin{aligned} o_{199} + 2o_{209} + 2o_{264} + 2o_{268} + o_{350} & \\ +o_{354} + 4o_{374} + 4o_{378} &= \sum_{P_{41}(x,a,b,c,d)} (c(x,c)-1) + (c(x,d)-1) \end{aligned}$$

$$o_{200} + 2o_{311} = \sum_{P_{21}(x,a,b,c,d)} c(x,a,b,c) + c(x,a,b,d)$$

$$o_{201} + 2o_{261} + 3o_{278} + o_{399} + 2o_{416} + o_{425} = \sum_{P_{45}(x,a,b,c,d)} (c(a,c)-2)$$

$$2o_{202} + o_{262} + o_{347} + o_{368} = \sum_{P_{37}(x,a,b,c,d)} c(a,c,d)$$

$$o_{203} + o_{262} + 2o_{264} + 2o_{280} + o_{400} + o_{417} + 2o_{418} + o_{427} = \sum_{P_{48}(x,a,b,c,d)} (c(a,c)-2)$$

$$o_{204} + 2o_{263} + 3o_{279} + o_{401} + o_{417} + o_{426} = \sum_{P_{46}(x,a,b,c,d)} (c(x,b)-2)$$

$$o_{205} + 2o_{265} + 3o_{281} + o_{403} + o_{419} + o_{428} = \sum_{P_{47}(x,a,b,c,d)} (c(x,b)-2)$$

$$2o_{206} + 4o_{266} + o_{352} + 4o_{376} = \sum_{P_{39}(x,a,b,c,d)} c(a,b,d) + c(a,c,d)$$

$$2o_{207} + 2o_{267} + o_{352} + 2o_{377} = \sum_{P_{40}(x,a,b,c,d)} c(a,b,d)$$

$$o_{208} + 2o_{267} + 2o_{353} + 2o_{377} = \sum_{P_{40}(x,a,b,c,d)} c(x,a,d)$$

$$2o_{209} + 2o_{268} + o_{354} + 4o_{378} = \sum_{P_{41}(x,a,b,c,d)} c(x,a,c) + c(x,a,d)$$

$$2o_{210} + 4o_{269} + 2o_{355} + 2o_{379} = \sum_{P_{42}(x,a,b,c,d)} c(x,a,d) + c(x,b,d)$$

$$o_{211} + 2o_{216} + o_{220} + 2o_{225} + o_{298} + o_{301} + o_{304} + o_{309} = \sum_{P_{18}(x,a,b,c,d)} (c(c,d) - 1)$$

$$o_{212} + 2o_{217} + o_{221} + 2o_{226} + 2o_{297} + 2o_{300} + o_{303} + o_{308} = \sum_{P_{19}(x,a,b,c,d)} (c(x,d) - 1)$$

$$2o_{213} + o_{230} + 2o_{239} + o_{244} + o_{330} + o_{332} + o_{333} + o_{338} = \sum_{P_{15}(x,a,b,c,d)} (c(x,b) - 1)$$

$$o_{214} + o_{218} + o_{223} + o_{227} + o_{299} + o_{302} + o_{306} + o_{311} = \sum_{P_{21}(x,a,b,c,d)} (c(c,d) - 1)$$

$$o_{215} + 2o_{219} + o_{224} + 2o_{228} + o_{298} + o_{301} + o_{305} + o_{310} = \sum_{P_{20}(x,a,b,c,d)} (c(b,d) - 1)$$

$$2o_{216} + 2o_{225} + o_{304} + o_{309} = \sum_{P_{18}(x,a,b,c,d)} (c(a,c,d) - 1)$$

$$2o_{217} + 2o_{226} + o_{303} + o_{308} = \sum_{P_{19}(x,a,b,c,d)} (c(x,b,d) - 1)$$

$$o_{218} + o_{227} + o_{306} + o_{311} = \sum_{P_{21}(x,a,b,c,d)} (c(a,c,d) - 1)$$

$$2o_{219} + 2o_{228} + o_{305} + o_{310} = \sum_{P_{20}(x,a,b,c,d)} (c(x,b,d) - 1)$$

$$o_{220} + 2o_{225} + o_{301} + o_{309} = \sum_{P_{18}(x,a,b,c,d)} c(b,c,d)$$

$$o_{221} + 2o_{226} + 2o_{300} + o_{308} = \sum_{P_{19}(x,a,b,c,d)} c(x,a,d)$$

$$o_{222} + 2o_{295} + o_{302} + o_{311} + o_{336} + o_{340} + o_{350}$$
$$+ o_{354} + o_{364} + o_{369} + 2o_{374} + 2o_{378} = \sum_{P_{41}(x,a,b,c,d)} (c(b) - 1)$$

$$o_{223} + o_{227} + o_{302} + o_{311} = \sum_{P_{21}(x,a,b,c,d)} c(x,c,d)$$

$$o_{224} + 2o_{228} + o_{301} + o_{310} = \sum_{P_{20}(x,a,b,c,d)} c(a,b,d)$$

$$2o_{225} + o_{309} = \sum_{P_{18}(x,a,b,c,d)} c(a,b,c,d)$$

$$2o_{226} + o_{308} = \sum_{P_{19}(x,a,b,c,d)} c(x,a,b,d)$$

$$o_{227} + o_{311} = \sum_{P_{21}(x,a,b,c,d)} c(x,a,c,d)$$

$$2o_{228} + o_{310} = \sum_{P_{20}(x,a,b,c,d)} c(x,a,b,d)$$

$$2o_{229} + o_{243} + o_{332} + o_{338} = \sum_{P_{15}(x,a,b,c,d)} c(a,b,d)$$

$$o_{230} + o_{244} + o_{333} + o_{338} = \sum_{P_{15}(x,a,b,c,d)} c(x,b,c)$$

$$o_{231} + o_{245} + o_{334} + 2o_{339} = \sum_{P_{17}(x,a,b,c,d)} c(x,b,c) + c(x,a,d)$$

$$2o_{232} + o_{248} + o_{336} + o_{340} = \sum_{P_{16}(x,a,b,c,d)} c(x,a,c)$$

$$o_{233} + 4o_{254} + o_{261} + 2o_{266} + o_{362} + o_{367} + 2o_{372} + 2o_{376} = \sum_{P_{39}(x,a,b,c,d)} (c(b,c) - 2)$$

$$o_{234} + o_{263} + 2o_{360} + o_{369} = \sum_{P_{36}(x,a,b,c,d)} c(a,b,d)$$

$$o_{235} + o_{255} + o_{264} + o_{268} + o_{364} + o_{369} + 2o_{374} + 2o_{378} = \sum_{P_{41}(x,a,b,c,d)} (c(c,d) - 2)$$

$$o_{236} + 2o_{255} + o_{262} + 2o_{267} + o_{363} + o_{368} + 2o_{373} + 2o_{377} = \sum_{P_{40}(x,a,b,c,d)} (c(x,b) - 2)$$

$$o_{237} + 4o_{256} + o_{265} + 2o_{269} + o_{366} + o_{371} + o_{375} + o_{379} = \sum_{P_{42}(x,a,b,c,d)} (c(a,b) - 2)$$

$$2o_{238} + o_{243} + o_{333} + o_{338} = \sum_{P_{15}(x,a,b,c,d)} c(b,c,d)$$

$$2o_{239} + o_{244} + o_{332} + o_{338} = \sum_{P_{15}(x,a,b,c,d)} c(x,a,b)$$

$$2o_{240} + o_{245} + o_{334} + 2o_{339} = \sum_{P_{17}(x,a,b,c,d)} c(x,a,b) + c(x,c,d)$$

$$o_{241} + o_{246} + o_{336} + o_{340} = \sum_{P_{16}(x,a,b,c,d)} c(x,a,d)$$

$$2o_{242} + o_{248} + o_{335} + o_{340} = \sum_{P_{16}(x,a,b,c,d)} c(a,b,c)$$

$$o_{243} + o_{338} = \sum_{P_{15}(x,a,b,c,d)} c(a,b,c,d)$$

$$o_{244} + o_{338} = \sum_{P_{15}(x,a,b,c,d)} c(x,a,b,c)$$

$$o_{245} + 2o_{339} = \sum_{P_{17}(x,a,b,c,d)} c(x,a,b,c) + c(x,a,c,d)$$

$$o_{246} + o_{340} = \sum_{P_{16}(x,a,b,c,d)} c(x,a,b,d)$$

$$o_{247} + 2o_{264} + 2o_{276} + 2o_{280} + 2o_{414} + 2o_{418} + o_{422} + o_{427} = \sum_{P_{48}(x,a,b,c,d)} (c(x,c) - 1)$$

$$o_{248} + o_{340} = \sum_{P_{16}(x,a,b,c,d)} c(x,a,b,c)$$

$$o_{249} + 3o_{278} + 2o_{391} + o_{425} = \sum_{P_{45}(x,a,b,c,d)} c(a,b,d)$$

$$o_{250} + 3o_{279} + 2o_{391} + o_{426} = \sum_{P_{46}(x,a,b,c,d)} c(a,b,d)$$

$$2o_{251} + 2o_{353} = \sum_{P_{25}(x,a,b,c,d)} c(x,a,b,c) + c(x,a,c,d)$$

$$o_{252} + 2o_{280} + 2o_{392} + o_{427} = \sum_{P_{48}(x,a,b,c,d)} c(x,a,d)$$

$$o_{253} + 3o_{281} + o_{392} + o_{428} = \sum_{P_{47}(x,a,b,c,d)} c(x,a,d)$$

$$4o_{254} + 2o_{266} + o_{367} + 2o_{376} = \sum_{P_{39}(x,a,b,c,d)} (c(a,b,c) - 1)$$

$$o_{255} + o_{268} + o_{369} + 2o_{378} = \sum_{P_{41}(x,a,b,c,d)} (c(a,c,d) - 1)$$

$$4o_{256} + 2o_{269} + o_{371} + o_{379} = \sum_{P_{42}(x,a,b,c,d)} (c(x,a,b) - 1)$$

$$2o_{257} + 2o_{274} + o_{399} + o_{407} = \sum_{P_{27}(x,a,b,c,d)} (c(a,c,d) - 1)$$

$$2o_{258} + o_{262} + o_{363} + o_{368} = \sum_{P_{37}(x,a,b,c,d)} c(x,c,d)$$

$$o_{259} + o_{263} + o_{364} + o_{369} = \sum_{P_{36}(x,a,b,c,d)} c(x,a,d)$$

$$2o_{260} + 2o_{277} + o_{402} + o_{410} = \sum_{P_{28}(x,a,b,c,d)} (c(x,b,d) - 1)$$

$$2o_{261} + 6o_{278} + 2o_{416} + 2o_{425} = \sum_{P_{45}(x,a,b,c,d)} (c(a,b,c) - 1) + (c(a,c,d) - 1)$$

$$o_{262} + 2o_{280} + o_{417} + o_{427} = \sum_{P_{48}(x,a,b,c,d)} (c(a,c,d) - 1)$$

$$2o_{263} + 6o_{279} + o_{417} + 2o_{426} = \sum_{P_{46}(x,a,b,c,d)} (c(x,a,b) - 1) + (c(x,b,d) - 1)$$

$$o_{264} + o_{268} + 2o_{374} + 2o_{378} = \sum_{P_{41}(x,a,b,c,d)} (c(x,c,d) - 1)$$

$$2o_{265} + 6o_{281} + o_{419} + 2o_{428} = \sum_{P_{47}(x,a,b,c,d)} (c(x,a,b) - 1) + (c(x,b,d) - 1)$$

$$2o_{266} + 2o_{376} = \sum_{P_{39}(x,a,b,c,d)} c(a,b,c,d)$$

$$2o_{267} + 2o_{377} = \sum_{P_{40}(x,a,b,c,d)} c(x,a,b,d)$$

$$o_{268} + 2o_{378} = \sum_{P_{41}(x,a,b,c,d)} c(x,a,c,d)$$

$$2o_{269} + o_{379} = \sum_{P_{42}(x,a,b,c,d)} c(x,a,b,d)$$

$$3o_{270} + 2o_{274} + o_{404} + o_{407} = \sum_{P_{27}(x,a,b,c,d)} c(b,c,d)$$

$$2o_{271} + 2o_{275} + 2o_{405} + o_{408} = \sum_{P_{29}(x,a,b,c,d)} c(x,a,d)$$

$$3o_{272} + o_{276} + o_{406} + o_{409} = \sum_{P_{30}(x,a,b,c,d)} c(x,c,d)$$

$$3o_{273} + 2o_{277} + o_{404} + o_{410} = \sum_{P_{28}(x,a,b,c,d)} c(a,b,d)$$

$$2o_{274} + 3o_{278} + o_{420} + o_{425} = \sum_{P_{45}(x,a,b,c,d)} c(b,c,d)$$

$$o_{275} + 3o_{279} + o_{421} + o_{426} = \sum_{P_{46}(x,a,b,c,d)} c(x,a,d)$$

$$2o_{276} + 2o_{280} + o_{422} + o_{427} = \sum_{P_{48}(x,a,b,c,d)} c(x,c,d)$$

$$2o_{277} + 3o_{281} + o_{423} + o_{428} = \sum_{P_{47}(x,a,b,c,d)} c(a,b,d)$$

$$3o_{278} + o_{425} = \sum_{P_{45}(x,a,b,c,d)} c(a,b,c,d)$$

$$3o_{279} + o_{426} = \sum_{P_{46}(x,a,b,c,d)} c(x,a,b,d)$$

$$2o_{280} + o_{427} = \sum_{P_{48}(x,a,b,c,d)} c(x,a,c,d)$$

$$3o_{281} + o_{428} = \sum_{P_{47}(x,a,b,c,d)} c(x,a,b,d)$$

$$4o_{282} + 2o_{433} = \sum_{P_{56}(x,a,b,c,d)} c(a,b,c,d)$$

$$3o_{283} + 2o_{434} = \sum_{P_{57}(x,a,b,c,d)} c(x,a,c,d)$$

$$4o_{284} + o_{435} = \sum_{P_{58}(x,a,b,c,d)} c(x,a,c,d)$$

$$2o_{285} + 2o_{287} = \sum_{P_{22}(x,a,b,c,d)} (c(x,b,c,d) - 1)$$

$$o_{286} + o_{288} = \sum_{P_{23}(x,a,b,c,d)} (c(a,b,c,d) - 1)$$

$$3o_{287}+2o_{316}+o_{317}+o_{376}+o_{377}+3o_{443}=\sum_{P_{54}(x,a,b,c,d)}(c(a,d)-3)$$

$$4o_{288}+2o_{318}+o_{379}+2o_{444}=\sum_{P_{55}(x,a,b,c,d)}(c(x,d)-3)$$

$$2o_{289}+o_{303}+o_{333}+o_{334}+o_{347}+o_{348}+2o_{359}+o_{368}=\sum_{P_{37}(x,a,b,c,d)}c(a,b)$$

$$2o_{290}+o_{332}+o_{336}+2o_{383}+o_{388}+o_{389}+2o_{405}+o_{408}=\sum_{P_{29}(x,a,b,c,d)}(c(a,c)-1)$$

$$2o_{291}+o_{306}+o_{335}+o_{349}+2o_{360}+o_{369}=\sum_{P_{36}(x,a,b,c,d)}c(b,c)$$

$$2o_{292}+o_{337}+2o_{384}+o_{390}+o_{406}+o_{409}=\sum_{P_{30}(x,a,b,c,d)}(c(x,b)-1)$$

$$o_{293}+o_{338}+2o_{394}+o_{407}+o_{408}+2o_{447}=\sum_{P_{43}(x,a,b,c,d)}(c(a,d)-1)$$

$$\begin{aligned}2o_{294}+o_{338}+o_{340}+6o_{393}+2o_{407}&\\+o_{408}+2o_{410}+4o_{447}&=\sum_{P_{43}(x,a,b,c,d)}(c(a,b)-1)+(c(a,c)-1)\end{aligned}$$

$$2o_{295}+o_{311}+o_{340}+o_{354}+o_{369}+2o_{378}=\sum_{P_{41}(x,a,b,c,d)}c(a,b)$$

$$2o_{296}+o_{312}+2o_{341}+2o_{355}+o_{371}+o_{379}=\sum_{P_{42}(x,a,b,c,d)}c(x,c)$$

$$2o_{297}+2o_{300}+o_{303}+o_{308}=\sum_{P_{19}(x,a,b,c,d)}c(x,c,d)$$

$$o_{298}+o_{301}+o_{304}+o_{309}=\sum_{P_{18}(x,a,b,c,d)}c(x,c,d)$$

$$o_{299}+o_{302}+o_{306}+o_{311}=\sum_{P_{21}(x,a,b,c,d)}c(b,c,d)$$

$$2o_{300}+o_{308}=\sum_{P_{19}(x,a,b,c,d)}c(x,a,c,d)$$

$$o_{301}+o_{309}=\sum_{P_{18}(x,a,b,c,d)}c(x,b,c,d)$$

$$o_{302} + o_{311} = \sum_{P_{21}(x,a,b,c,d)} c(x,b,c,d)$$

$$o_{303} + o_{308} = \sum_{P_{19}(x,a,b,c,d)} c(x,b,c,d)$$

$$o_{304} + o_{309} = \sum_{P_{18}(x,a,b,c,d)} c(x,a,c,d)$$

$$o_{305} + o_{310} = \sum_{P_{20}(x,a,b,c,d)} c(x,b,c,d)$$

$$o_{306} + o_{311} = \sum_{P_{21}(x,a,b,c,d)} c(a,b,c,d)$$

$$2o_{307} + o_{351} + 2o_{366} + 2o_{403} + o_{419} + 2o_{424} + o_{454} + o_{457} = \sum_{P_{53}(x,a,b,c,d)} (c(x,b) - 2)$$

$$o_{308} + o_{352} + 2o_{353} + 2o_{377} = \sum_{P_{40}(x,a,b,c,d)} c(a,c,d)$$

$$o_{309} + o_{352} + 2o_{376} = \sum_{P_{39}(x,a,b,c,d)} c(x,a,d)$$

$$o_{310} + 2o_{317} = \sum_{P_{32}(x,a,b,c,d)} c(x,a,b,c)$$

$$o_{311} + o_{354} + 2o_{378} = \sum_{P_{41}(x,a,b,c,d)} c(x,a,b)$$

$$o_{312} + 2o_{355} + o_{379} = \sum_{P_{42}(x,a,b,c,d)} c(x,c,d)$$

$$2o_{313} + 2o_{372} + o_{373} + 2o_{440} = \sum_{P_{49}(x,a,b,c,d)} c(a,b,d) + c(a,c,d)$$

$$o_{314} + o_{373} + o_{440} = \sum_{P_{49}(x,a,b,c,d)} c(x,a,d)$$

$$2o_{315} + 2o_{375} + 3o_{441} = \sum_{P_{50}(x,a,b,c,d)} c(x,a,b) + c(x,b,c) + c(x,b,d)$$

$$o_{316} + 2o_{376} + 3o_{433} + o_{447} + o_{462} + 3o_{471} = \sum_{P_{65}(x,a,b,c,d)} (c(a,d) - 3)$$

$$2o_{317} + o_{377} + 2o_{378} + 2o_{434} + 2o_{447} + o_{462} + o_{463} + 3o_{472} = \sum_{P_{66}(x,a,b,c,d)} (c(a,d) - 3)$$

$$2o_{318} + 2o_{379} + 3o_{435} + 4o_{448} + 2o_{464} + 2o_{473} = \sum_{P_{67}(x,a,b,c,d)} (c(x,d) - 3)$$

$$2o_{319} + 2o_{320} + o_{321} + o_{324} + o_{330} + o_{332} + o_{333} + o_{338} = \sum_{P_{15}(x,a,b,c,d)} c(x,d)$$

$$2o_{320} + o_{324} + o_{332} + o_{338} = \sum_{P_{15}(x,a,b,c,d)} c(x,a,d)$$

$$o_{321} + o_{324} + o_{333} + o_{338} = \sum_{P_{15}(x,a,b,c,d)} c(x,c,d)$$

$$2o_{322} + o_{326} + o_{335} + o_{340} = \sum_{P_{16}(x,a,b,c,d)} c(b,c,d)$$

$$o_{323} + o_{326} + o_{336} + o_{340} = \sum_{P_{16}(x,a,b,c,d)} c(x,c,d)$$

$$o_{324} + o_{338} = \sum_{P_{15}(x,a,b,c,d)} c(x,a,c,d)$$

$$o_{325} + o_{339} = \sum_{P_{17}(x,a,b,c,d)} c(a,b,c,d)$$

$$o_{326} + o_{340} = \sum_{P_{16}(x,a,b,c,d)} c(x,b,c,d)$$

$$2o_{327} + o_{350} + 2o_{403} + 2o_{414} + o_{419} + o_{422} + o_{454} + o_{457} = \sum_{P_{53}(x,a,b,c,d)} c(x,a)$$

$$o_{328} + o_{353} = \sum_{P_{25}(x,a,b,c,d)} c(a,b,c,d)$$

$$2o_{329} + o_{354} = \sum_{P_{26}(x,a,b,c,d)} c(x,a,b,c)$$

$$o_{330} + o_{332} + o_{333} + o_{338} = \sum_{P_{15}(x,a,b,c,d)} c(x,b,d)$$

$$o_{331} + o_{334} + o_{339} = \sum_{P_{17}(x,a,b,c,d)} c(x,b,d)$$

$$o_{332} + o_{338} = \sum_{P_{15}(x,a,b,c,d)} c(x,a,b,d)$$

$$o_{333} + o_{338} = \sum_{P_{15}(x,a,b,c,d)} c(x,b,c,d)$$

$$o_{334} + 2o_{339} = \sum_{P_{17}(x,a,b,c,d)} c(x,a,b,d) + c(x,b,c,d)$$

$$o_{335} + o_{340} = \sum_{P_{16}(x,a,b,c,d)} c(a,b,c,d)$$

$$o_{336} + o_{340} = \sum_{P_{16}(x,a,b,c,d)} c(x,a,c,d)$$

$$o_{337} + 2o_{361} + o_{390} + 2o_{392} + 2o_{414} + 2o_{418} + o_{422} + o_{427} = \sum_{P_{48}(x,a,b,c,d)} (c(x,b) - 1)$$

$$o_{338} + 2o_{407} + o_{408} + 4o_{447} = \sum_{P_{43}(x,a,b,c,d)} c(a,b,d) + c(a,c,d)$$

$$2o_{339} + 2o_{353} + o_{368} + 2o_{377} = \sum_{P_{40}(x,a,b,c,d)} c(x,a,c)$$

$$o_{340} + o_{354} + 2o_{369} + 4o_{378} = \sum_{P_{41}(x,a,b,c,d)} c(a,b,c) + c(a,b,d)$$

$$2o_{341} + 2o_{355} + 2o_{371} + 2o_{379} = \sum_{P_{42}(x,a,b,c,d)} c(x,a,c) + c(x,b,c)$$

$$o_{342} + 2o_{362} + 2o_{412} + 2o_{420} + 2o_{451} + o_{452} + o_{453} + o_{456} = \sum_{P_{51}(x,a,b,c,d)} (c(a,c) - 1)$$

$$2o_{343} + o_{363} + 2o_{414} + o_{422} + 2o_{451} + o_{453} + o_{454} + o_{457} = \sum_{P_{53}(x,a,b,c,d)} (c(a,c) - 1)$$

$$o_{344} + 2o_{366} + o_{415} + 2o_{424} + 2o_{451} + o_{453} + o_{454} + o_{457} = \sum_{P_{53}(x,a,b,c,d)} (c(b,c) - 1)$$

$$2o_{345} + 2o_{365} + o_{415} + 2o_{423} + 2o_{451} + o_{452} + o_{453} + o_{456} = \sum_{P_{51}(x,a,b,c,d)} (c(x,c) - 1)$$

$$o_{346} + 2o_{416} + 2o_{421} + 2o_{455} = \sum_{P_{52}(x,a,b,c,d)} c(a,b,d) + c(a,c,d)$$

$$o_{347} + o_{417} + 2o_{420} + o_{456} = \sum_{P_{51}(x,a,b,c,d)} c(a,b,d)$$

$$o_{348} + 2o_{353} + 2o_{373} + 2o_{377} = \sum_{P_{40}(x,a,b,c,d)} (c(x,c,d) - 1)$$

$$o_{349} + o_{417} + 2o_{423} + o_{456} = \sum_{P_{51}(x,a,b,c,d)} c(x,b,d)$$

$$o_{350} + o_{354} + 4o_{374} + 4o_{378} = \sum_{P_{41}(x,a,b,c,d)} (c(x,b,c) - 1) + (c(x,b,d) - 1)$$

$$o_{351} + o_{419} + 2o_{424} + o_{457} = \sum_{P_{53}(x,a,b,c,d)} c(x,b,d)$$

$$o_{352} + 2o_{377} = \sum_{P_{40}(x,a,b,c,d)} c(a,b,c,d)$$

$$2o_{353} + 2o_{377} = \sum_{P_{40}(x,a,b,c,d)} c(x,a,c,d)$$

$$o_{354} + 4o_{378} = \sum_{P_{41}(x,a,b,c,d)} c(x,a,b,c) + c(x,a,b,d)$$

$$2o_{355} + 2o_{379} = \sum_{P_{42}(x,a,b,c,d)} c(x,a,c,d) + c(x,b,c,d)$$

$$o_{356} + o_{362} + o_{372} + 3o_{436} + 2o_{437} + o_{440} = \sum_{P_{49}(x,a,b,c,d)} (c(b,c) - 2)$$

$$o_{357} + o_{363} + o_{373} + 6o_{436} + 4o_{437} + 2o_{440} = \sum_{P_{49}(x,a,b,c,d)} (c(x,b) - 2) + (c(x,c) - 2)$$

$$2o_{358} + 2o_{391} + o_{421} + o_{426} = \sum_{P_{46}(x,a,b,c,d)} (c(a,c,d) - 1)$$

$$o_{359} + o_{392} + o_{423} + o_{428} = \sum_{P_{47}(x,a,b,c,d)} (c(a,c,d) - 1)$$

$$2o_{360} + 2o_{391} + o_{420} + o_{425} = \sum_{P_{45}(x,a,b,c,d)} (c(x,b,d) - 1)$$

$$2o_{361} + 2o_{392} + o_{422} + o_{427} = \sum_{P_{48}(x,a,b,c,d)} (c(x,b,d) - 1)$$

$$o_{362} + 2o_{372} + 2o_{437} + 2o_{440} = \sum_{P_{49}(x,a,b,c,d)} c(a,b,c) + c(b,c,d)$$

$$o_{363} + 2o_{373} + 4o_{437} + 4o_{440} = \sum_{P_{49}(x,a,b,c,d)} c(x,a,b) + c(x,a,c) + c(x,b,d) + c(x,c,d)$$

$$o_{364} + o_{369} + 2o_{374} + 2o_{378} = \sum_{P_{41}(x,a,b,c,d)} (c(b,c,d) - 1)$$

$$2o_{365} + 2o_{423} + o_{453} + o_{456} = \sum_{P_{51}(x,a,b,c,d)} (c(x,b,c) - 1)$$

$$2o_{366} + 2o_{424} + o_{454} + o_{457} = \sum_{P_{53}(x,a,b,c,d)} (c(x,b,c) - 1)$$

$$o_{367} + 2o_{376} = \sum_{P_{39}(x,a,b,c,d)} c(x,a,b,c)$$

$$o_{368} + 2o_{377} = \sum_{P_{40}(x,a,b,c,d)} c(x,a,b,c)$$

$$o_{369} + 2o_{378} = \sum_{P_{41}(x,a,b,c,d)} c(a,b,c,d)$$

$$o_{370} + 3o_{441} + o_{457} + o_{468} + 2o_{469} + 2o_{475} = \sum_{P_{68}(x,a,b,c,d)} (c(x,d) - 2)$$

$$o_{371} + o_{379} = \sum_{P_{42}(x,a,b,c,d)} c(x,a,b,c)$$

$$o_{372} + o_{440} = \sum_{P_{49}(x,a,b,c,d)} c(a,b,c,d)$$

$$o_{373} + 2o_{440} = \sum_{P_{49}(x,a,b,c,d)} c(x,a,b,d) + c(x,a,c,d)$$

$$2o_{374} + 2o_{378} = \sum_{P_{41}(x,a,b,c,d)} (c(x,b,c,d) - 1)$$

$$o_{375} + 3o_{441} = \sum_{P_{50}(x,a,b,c,d)} c(x,a,b,c) + c(x,a,b,d) + c(x,b,c,d)$$

$$2o_{376} + 6o_{433} + o_{462} + 6o_{471} = \sum_{P_{65}(x,a,b,c,d)} (c(a,b,d) - 1) + (c(a,c,d) - 1)$$

$$o_{377} + 2o_{434} + o_{462} + 3o_{472} = \sum_{P_{66}(x,a,b,c,d)} (c(a,c,d) - 1)$$

$$2o_{378} + 2o_{434} + o_{463} + 3o_{472} = \sum_{P_{66}(x,a,b,c,d)} (c(x,a,d) - 1)$$

$$2o_{379} + 6o_{435} + 2o_{464} + 4o_{473} = \sum_{P_{67}(x,a,b,c,d)} (c(x,a,d) - 1) + (c(x,c,d) - 1)$$

$$2o_{380} + 2o_{383} + 2o_{386} + o_{387} + o_{388} + o_{389} + o_{400} + o_{408} = \sum_{P_{29}(x,a,b,c,d)} c(b,c)$$

$$2o_{381} + 2o_{384} + o_{387} + o_{390} + o_{401} + o_{409} = \sum_{P_{30}(x,a,b,c,d)} c(a,b)$$

$$2o_{382} + o_{388} + 2o_{391} + o_{401} + o_{417} + o_{426} = \sum_{P_{46}(x,a,b,c,d)} c(b,c)$$

$$o_{383} + 3o_{393} + o_{399} + o_{402} + o_{407} + o_{410} + 2o_{445} + 2o_{447} = \sum_{P_{43}(x,a,b,c,d)} (c(b,c) - 1)$$

$$2o_{384} + o_{390} + o_{392} + o_{403} + o_{419} + o_{428} = \sum_{P_{47}(x,a,b,c,d)} c(x,c)$$

$$2o_{385} + o_{389} + o_{390} + 2o_{392} + o_{400} + o_{417} + 2o_{418} + o_{427} = \sum_{P_{48}(x,a,b,c,d)} c(a,b)$$

$$o_{386} + 2o_{399} + o_{416} + o_{451} + o_{452} + o_{455} = \sum_{P_{52}(x,a,b,c,d)} c(b,c)$$

$$o_{387} + o_{400} + 2o_{401} + o_{417} + 2o_{451} + o_{452} + o_{453} + o_{456} = \sum_{P_{51}(x,a,b,c,d)} c(x,a)$$

$$o_{388} + 4o_{391} + o_{417} + 2o_{426} = \sum_{P_{46}(x,a,b,c,d)} c(a,b,c) + c(b,c,d)$$

$$o_{389} + 2o_{392} + o_{417} + o_{427} = \sum_{P_{48}(x,a,b,c,d)} c(a,b,d)$$

$$o_{390} + 2o_{392} + 2o_{418} + o_{427} = \sum_{P_{48}(x,a,b,c,d)} c(x,a,b)$$

$$2o_{391} + o_{426} = \sum_{P_{46}(x,a,b,c,d)} c(a,b,c,d)$$

$$2o_{392} + o_{427} = \sum_{P_{48}(x,a,b,c,d)} c(x,a,b,d)$$

$$3o_{393} + o_{407} + o_{410} + 2o_{447} = \sum_{P_{43}(x,a,b,c,d)} c(a,b,c)$$

$$2o_{394} + o_{408} + 2o_{447} = \sum_{P_{43}(x,a,b,c,d)} c(x,a,d)$$

$$3o_{395} + o_{411} + 2o_{448} = \sum_{P_{44}(x,a,b,c,d)} c(x,a,c) + c(x,b,d)$$

$$o_{396} + o_{399} + 2o_{412} + 2o_{416} + o_{420} + o_{425} = \sum_{P_{45}(x,a,b,c,d)} c(x,c)$$

$$2o_{397} + o_{400} + o_{413} + 2o_{414} + o_{417} + 2o_{418} + o_{422} + o_{427} = \sum_{P_{48}(x,a,b,c,d)} c(b,c)$$

$$o_{398} + o_{403} + o_{415} + o_{419} + o_{423} + o_{428} = \sum_{P_{47}(x,a,b,c,d)} c(b,c)$$

$$o_{399} + o_{407} + 2o_{445} + 2o_{447} = \sum_{P_{43}(x,a,b,c,d)} (c(b,c,d) - 1)$$

$$o_{400} + o_{417} + 2o_{418} + o_{427} = \sum_{P_{48}(x,a,b,c,d)} c(a,b,c)$$

$$2o_{401} + o_{417} + o_{453} + o_{456} = \sum_{P_{51}(x,a,b,c,d)} c(x,a,b)$$

$$2o_{402} + o_{419} + o_{453} + o_{457} = \sum_{P_{53}(x,a,b,c,d)} c(a,b,d)$$

$$o_{403} + o_{419} + o_{428} = \sum_{P_{47}(x,a,b,c,d)} c(x,b,c)$$

$$2o_{404} + 2o_{420} + 2o_{423} + o_{456} = \sum_{P_{51}(x,a,b,c,d)} c(b,c,d)$$

$$o_{405} + 2o_{421} + o_{455} = \sum_{P_{52}(x,a,b,c,d)} c(x,a,d)$$

$$2o_{406} + o_{422} + 2o_{424} + o_{457} = \sum_{P_{53}(x,a,b,c,d)} c(x,c,d)$$

$$o_{407} + 2o_{447} = \sum_{P_{43}(x,a,b,c,d)} c(a,b,c,d)$$

$$o_{408} + 4o_{447} = \sum_{P_{43}(x,a,b,c,d)} c(x,a,b,d) + c(x,a,c,d)$$

$$o_{409} + 4o_{448} + o_{457} + 2o_{464} + o_{469} + 2o_{473} = \sum_{P_{67}(x,a,b,c,d)} (c(b,d) - 1)$$

$$o_{410} + 2o_{447} = \sum_{P_{43}(x,a,b,c,d)} c(x,a,b,c)$$

$$o_{411} + 4o_{448} = \sum_{P_{44}(x,a,b,c,d)} c(x,a,b,c) + c(x,a,b,d) + c(x,a,c,d) + c(x,b,c,d)$$

$$2o_{412} + 2o_{420} + o_{453} + o_{456} = \sum_{P_{51}(x,a,b,c,d)} c(a,b,c)$$

$$o_{413} + o_{417} + o_{422} + o_{427} = \sum_{P_{48}(x,a,b,c,d)} c(b,c,d)$$

$$2o_{414} + 2o_{418} + o_{422} + o_{427} = \sum_{P_{48}(x,a,b,c,d)} c(x, b, c)$$

$$o_{415} + o_{419} + 2o_{423} + 2o_{428} = \sum_{P_{47}(x,a,b,c,d)} c(a, b, c) + c(b, c, d)$$

$$2o_{416} + 2o_{425} = \sum_{P_{45}(x,a,b,c,d)} c(x, a, b, c) + c(x, a, c, d)$$

$$o_{417} + o_{427} = \sum_{P_{48}(x,a,b,c,d)} c(a, b, c, d)$$

$$2o_{418} + o_{427} = \sum_{P_{48}(x,a,b,c,d)} c(x, a, b, c)$$

$$o_{419} + 2o_{428} = \sum_{P_{47}(x,a,b,c,d)} c(x, a, b, c) + c(x, b, c, d)$$

$$2o_{420} + o_{456} = \sum_{P_{51}(x,a,b,c,d)} c(a, b, c, d)$$

$$o_{421} + o_{426} = \sum_{P_{46}(x,a,b,c,d)} c(x, a, c, d)$$

$$o_{422} + o_{427} = \sum_{P_{48}(x,a,b,c,d)} c(x, b, c, d)$$

$$o_{423} + o_{428} = \sum_{P_{47}(x,a,b,c,d)} c(a, b, c, d)$$

$$2o_{424} + o_{457} = \sum_{P_{53}(x,a,b,c,d)} c(x, b, c, d)$$

$$o_{425} + 6o_{433} + 2o_{467} + 6o_{471} = \sum_{P_{65}(x,a,b,c,d)} (c(a, b, c) - 1) + (c(b, c, d) - 1)$$

$$2o_{426} + 2o_{462} = \sum_{P_{59}(x,a,b,c,d)} c(x, a, b, d)$$

$$o_{427} + 4o_{434} + 2o_{468} + 6o_{472} = \sum_{P_{66}(x,a,b,c,d)} (c(x, a, c) - 1) + (c(x, c, d) - 1)$$

$$o_{428} + 3o_{435} + o_{469} + 2o_{473} = \sum_{P_{67}(x,a,b,c,d)} (c(a, c, d) - 1)$$

$$o_{429} + 3o_{435} + 2o_{470} + 2o_{473} = \sum_{P_{67}(x,a,b,c,d)} (c(x, a, c) - 1)$$

$$2o_{430} + 2o_{433} = \sum_{P_{56}(x,a,b,c,d)} (c(x,b,c,d) - 1)$$

$$2o_{431} + 2o_{434} = \sum_{P_{57}(x,a,b,c,d)} (c(x,b,c,d) - 1)$$

$$3o_{432} + o_{469} = \sum_{P_{63}(x,a,b,c,d)} c(x,a,b,d)$$

$$3o_{433} + 3o_{471} = \sum_{P_{65}(x,a,b,c,d)} c(a,b,c,d)$$

$$2o_{434} + 3o_{472} = \sum_{P_{66}(x,a,b,c,d)} c(x,a,c,d)$$

$$3o_{435} + 2o_{473} = \sum_{P_{67}(x,a,b,c,d)} c(x,a,c,d)$$

$$3o_{436} + 2o_{437} + o_{440} = \sum_{P_{49}(x,a,b,c,d)} (c(x,b,c) - 2)$$

$$2o_{437} + 2o_{440} = \sum_{P_{49}(x,a,b,c,d)} c(x,a,b,c) + c(x,b,c,d)$$

$$2o_{438} + o_{441} = \sum_{P_{50}(x,a,b,c,d)} c(a,b,c,d)$$

$$o_{439} + o_{441} = \sum_{P_{50}(x,a,b,c,d)} c(x,a,c,d)$$

$$2o_{440} + 2o_{467} + o_{468} + 2o_{475} = \sum_{P_{68}(x,a,b,c,d)} (c(a,c,d) - 2)$$

$$3o_{441} + 2o_{469} + 2o_{475} = \sum_{P_{68}(x,a,b,c,d)} (c(x,b,d) - 2)$$

$$3o_{442} + 2o_{470} + 2o_{476} = \sum_{P_{69}(x,a,b,c,d)} (c(x,a,b) - 2) + (c(x,c,d) - 2)$$

$$2o_{443} + 2o_{471} + o_{472} + 4o_{477} = \sum_{P_{70}(x,a,b,c,d)} (c(a,c,d) - 2)$$

$$3o_{444} + 2o_{473} + 3o_{478} = \sum_{P_{71}(x,a,b,c,d)} (c(x,c,d) - 2)$$

$$2o_{445} + 2o_{447} = \sum_{P_{43}(x,a,b,c,d)} (c(x,b,c,d) - 1)$$

$$o_{446} + o_{448} = \sum_{P_{44}(x,a,b,c,d)} (c(a,b,c,d) - 1)$$

$$o_{447} + o_{462} + 3o_{471} = \sum_{P_{65}(x,a,b,c,d)} c(x,a,d)$$

$$4o_{448} + 2o_{464} + 2o_{473} = \sum_{P_{67}(x,a,b,c,d)} c(x,b,d)$$

$$2o_{449} + 2o_{455} + o_{456} + 2o_{462} = \sum_{P_{59}(x,a,b,c,d)} c(x,c,d)$$

$$5o_{450} + 2o_{458} + o_{464} = \sum_{P_{61}(x,a,b,c,d)} c(x,b,c)$$

$$2o_{451} + o_{452} + o_{453} + o_{456} = \sum_{P_{51}(x,a,b,c,d)} c(x,a,c)$$

$$o_{452} + o_{456} = \sum_{P_{51}(x,a,b,c,d)} c(x,a,c,d)$$

$$o_{453} + o_{456} = \sum_{P_{51}(x,a,b,c,d)} c(x,a,b,c)$$

$$o_{454} + o_{457} = \sum_{P_{53}(x,a,b,c,d)} c(x,a,b,c)$$

$$2o_{455} + 2o_{462} + 2o_{468} + 6o_{472} = \sum_{P_{66}(x,a,b,c,d)} (c(a,b,c) - 1) + (c(b,c,d) - 1)$$

$$o_{456} + 4o_{467} + 2o_{469} + 4o_{475} = \sum_{P_{68}(x,a,b,c,d)} c(a,b,d) + c(b,c,d)$$

$$o_{457} + 2o_{464} + 2o_{469} + 4o_{473} = \sum_{P_{67}(x,a,b,c,d)} (c(a,b,d) - 1) + (c(b,c,d) - 1)$$

$$2o_{458} + 2o_{464} + 4o_{470} + 4o_{473} = \sum_{P_{67}(x,a,b,c,d)} (c(x,a,b) - 1) + (c(x,b,c) - 1)$$

$$o_{459} + 2o_{467} + 4o_{474} + 2o_{475} = \sum_{P_{68}(x,a,b,c,d)} (c(a,b,c) - 1)$$

$$o_{460} + o_{468} + 4o_{474} + 2o_{475} = \sum_{P_{68}(x,a,b,c,d)} (c(x,a,c) - 1)$$

$$2o_{461} + 2o_{469} + 8o_{474} + 4o_{475} = \sum_{P_{68}(x,a,b,c,d)} (c(x,a,b) - 1) + (c(x,b,c) - 1)$$

$$o_{462} + 6o_{471} = \sum_{P_{65}(x,a,b,c,d)} c(x,a,b,d) + c(x,a,c,d)$$

$$o_{463} + 3o_{472} = \sum_{P_{66}(x,a,b,c,d)} c(x,a,b,d)$$

$$2o_{464} + 4o_{473} = \sum_{P_{67}(x,a,b,c,d)} c(x,a,b,d) + c(x,b,c,d)$$

$$o_{465} + 2o_{467} + 3o_{471} = \sum_{P_{65}(x,a,b,c,d)} (c(x,b,c) - 2)$$

$$o_{466} + o_{469} + 2o_{470} + 2o_{473} = \sum_{P_{67}(x,a,b,c,d)} (c(a,b,c) - 2)$$

$$2o_{467} + 2o_{475} = \sum_{P_{68}(x,a,b,c,d)} c(a,b,c,d)$$

$$o_{468} + 2o_{475} = \sum_{P_{68}(x,a,b,c,d)} c(x,a,c,d)$$

$$o_{469} + 2o_{473} = \sum_{P_{67}(x,a,b,c,d)} (c(a,b,c,d) - 1)$$

$$2o_{470} + 2o_{473} = \sum_{P_{67}(x,a,b,c,d)} (c(x,a,b,c) - 1)$$

$$2o_{471} + 4o_{477} = \sum_{P_{70}(x,a,b,c,d)} c(a,b,c,d)$$

$$o_{472} + 4o_{477} + 2o_{478} + 20o_{479} = \sum_{P_{72}(x,a,b,c,d)} (c(a,b,c) - 2) + (c(a,b,d) - 2) + (c(a,c,d) - 2) + (c(b,c,d) - 2)$$

$$o_{473} + 6o_{478} + 30o_{479} = \sum_{P_{72}(x,a,b,c,d)} (c(x,a,b) - 2) + (c(x,a,c) - 2) + (c(x,b,c) - 2) + (c(x,a,d) - 2) + (c(x,b,d) - 2) + (c(x,c,d) - 2)$$

$$4o_{474} + 2o_{475} = \sum_{P_{68}(x,a,b,c,d)} (c(x,a,b,c) - 1)$$

$$2o_{475} + 12o_{477} = \sum_{P_{70}(x,a,b,c,d)} (c(x,a,b,c) - 1) + (c(x,a,b,d) - 1) + (c(x,b,c,d) - 1)$$

$$4o_{476} + 6o_{478} = \sum_{P_{71}(x,a,b,c,d)} (c(x,a,b,c) - 1) + (c(x,a,b,d) - 1)$$

$$o_{477} + 5o_{479} = \sum_{P_{72}(x,a,b,c,d)} (c(a,b,c,d) - 1)$$

$$\begin{aligned} 2o_{478} + 20o_{479} = \sum_{P_{72}(x,a,b,c,d)} & (c(x,a,b,c) - 1) + (c(x,a,b,d) - 1) \\ & + (c(x,a,c,d) - 1) + (c(x,b,c,d) - 1) \end{aligned}$$
