# Supplementary figures and images for: An Algorithm to Automatically Generate the Combinatorial Orbit Counting Equations
Source: PLoS One. 2016 Jan 21;11(1):e0147078. doi: 10.1371/journal.pone.0147078 (PMC4721873; doi:10.1371/journal.pone.0147078)

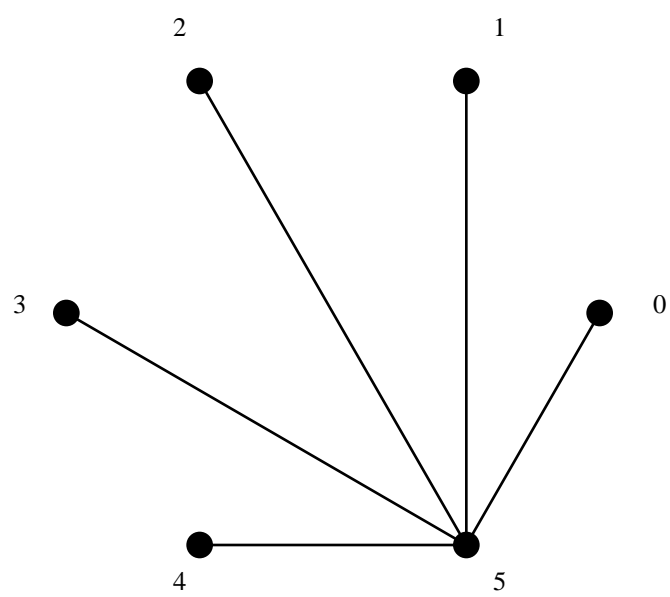

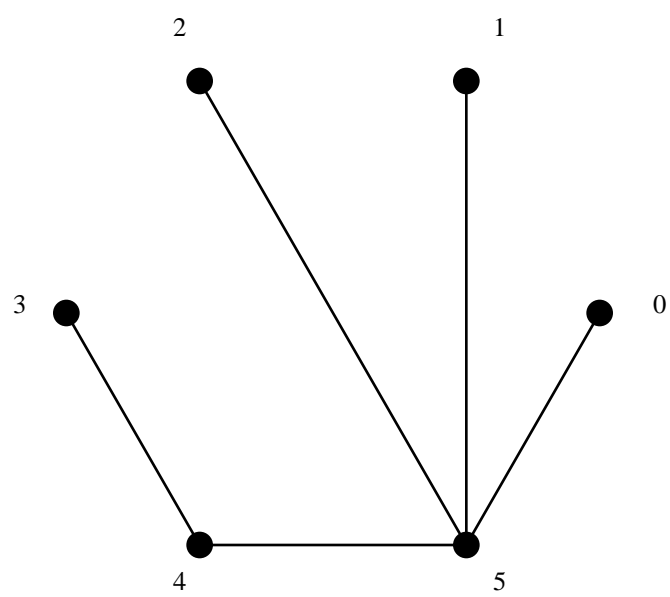

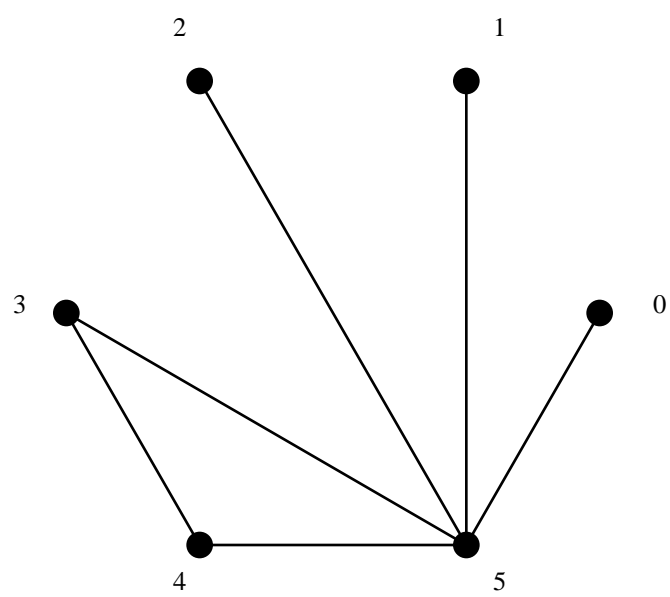

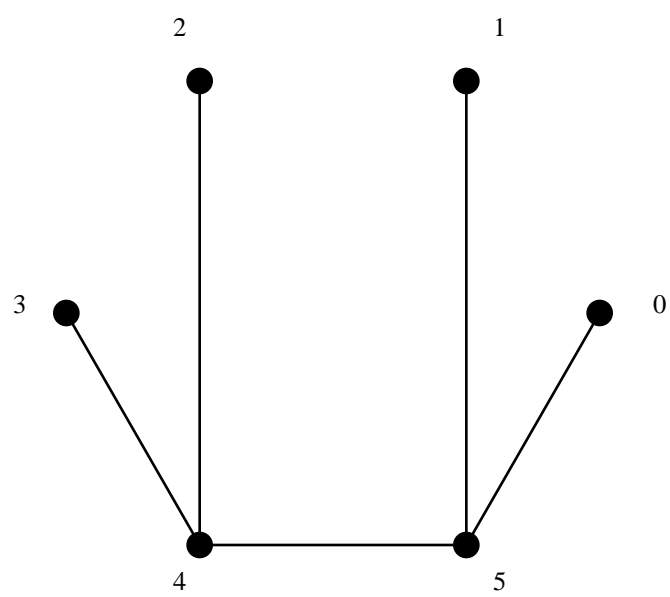

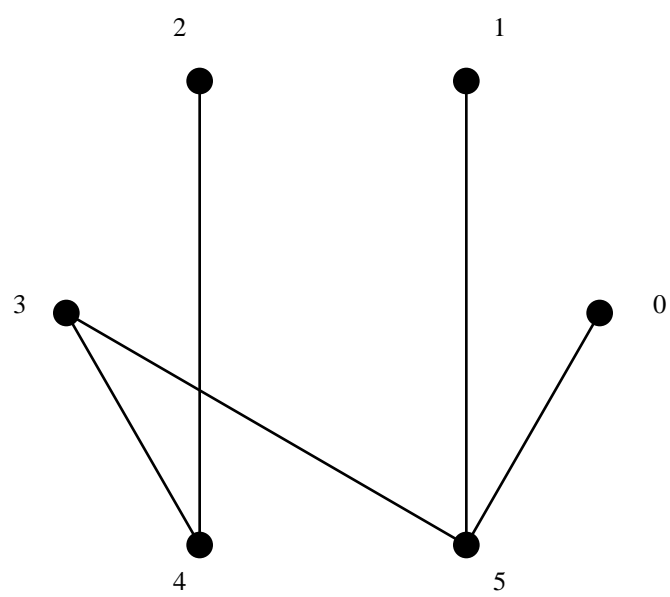

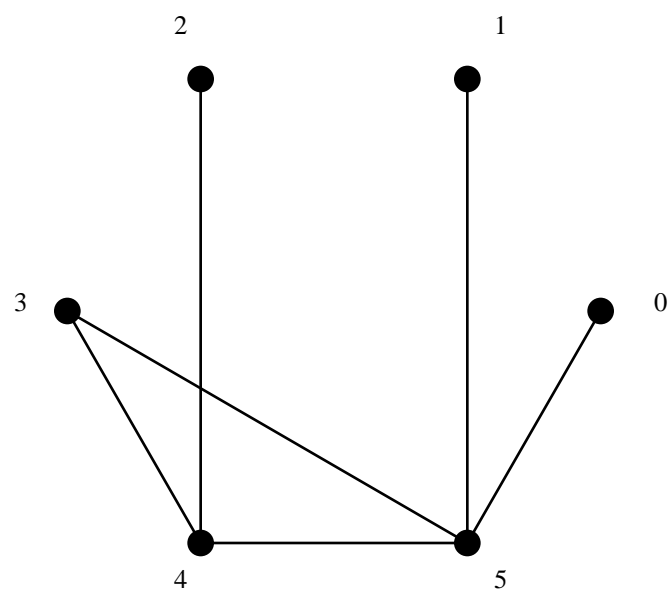

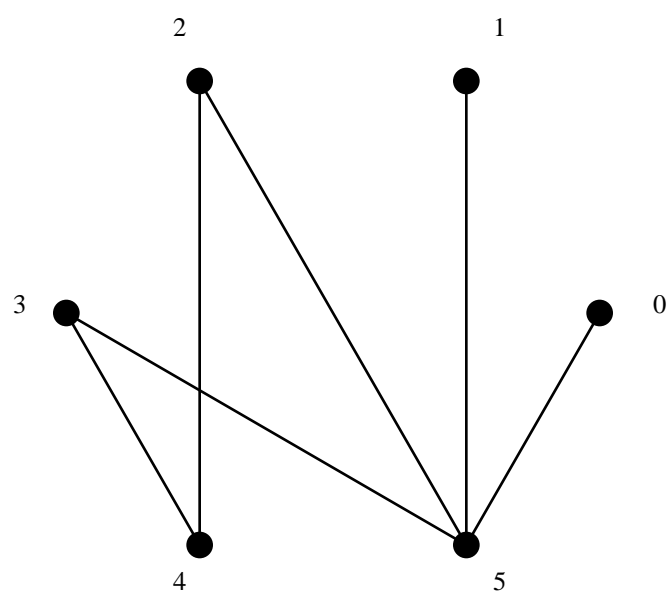

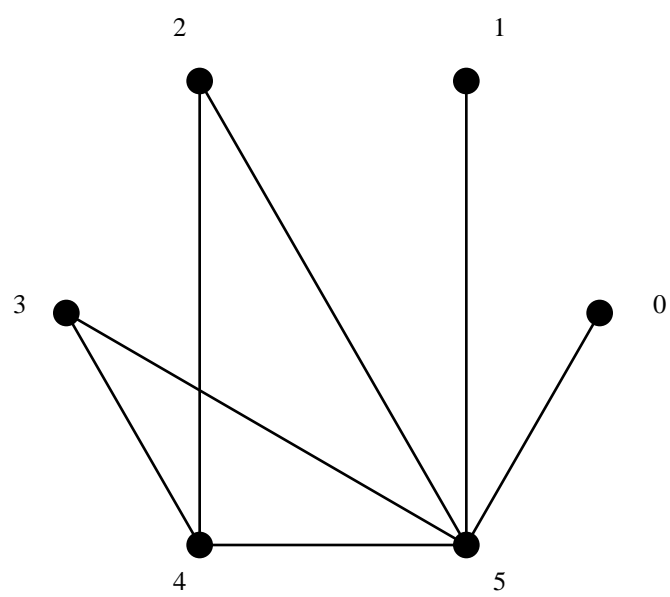

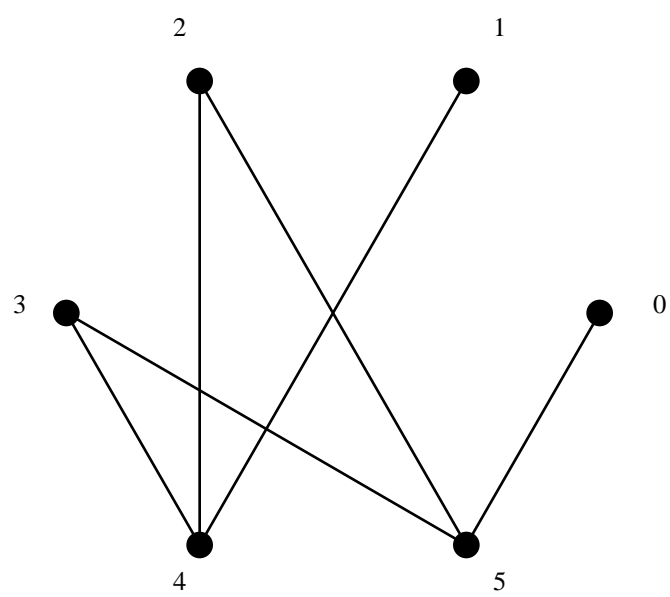

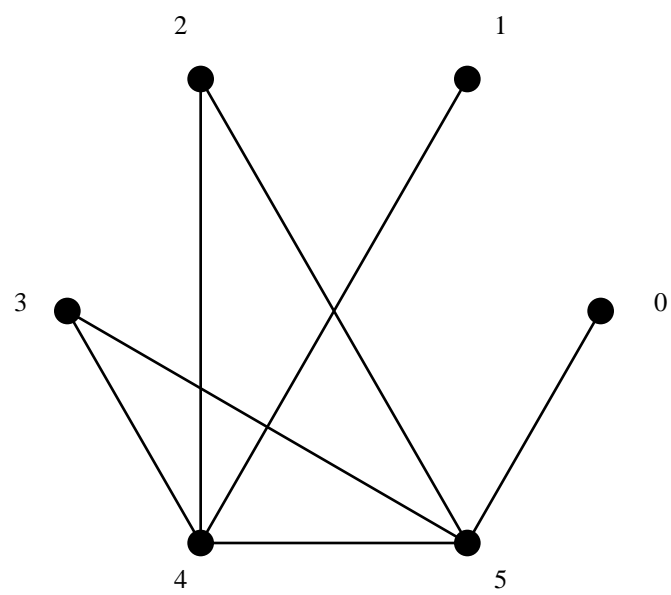

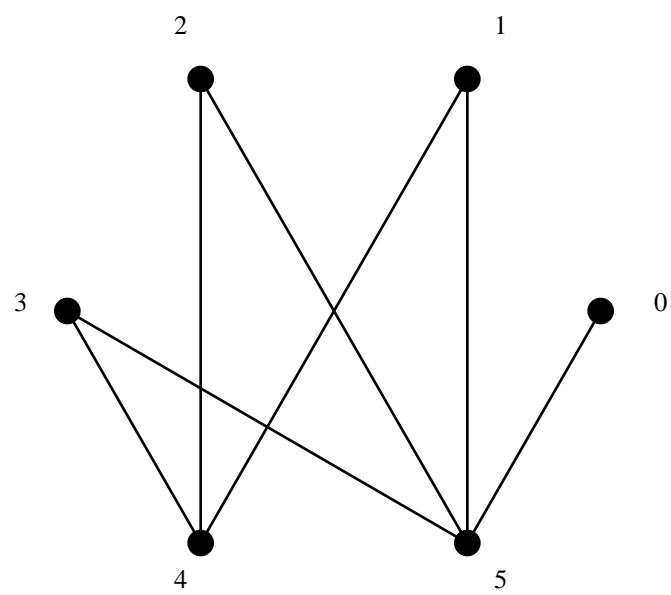

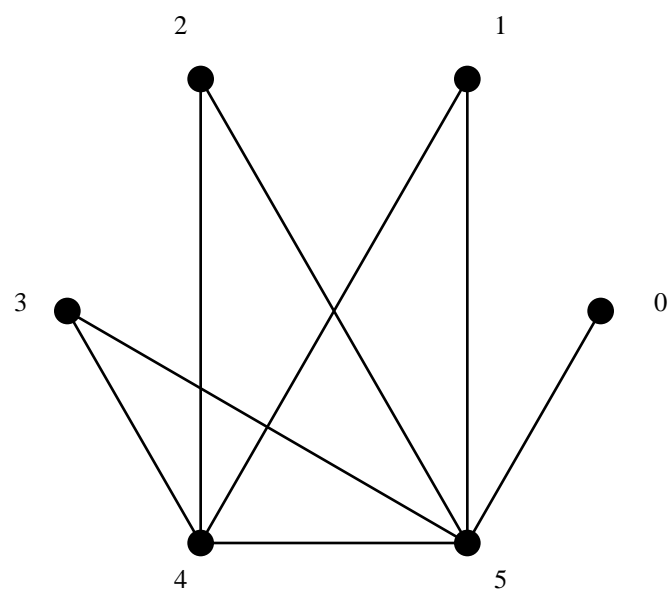

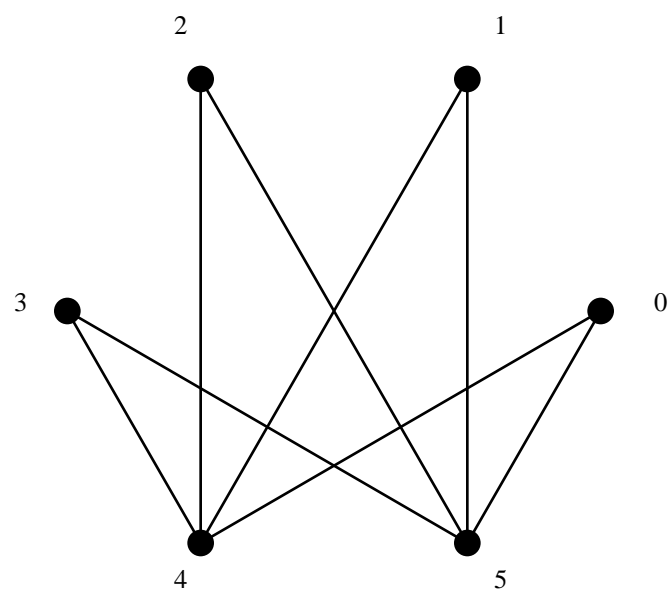

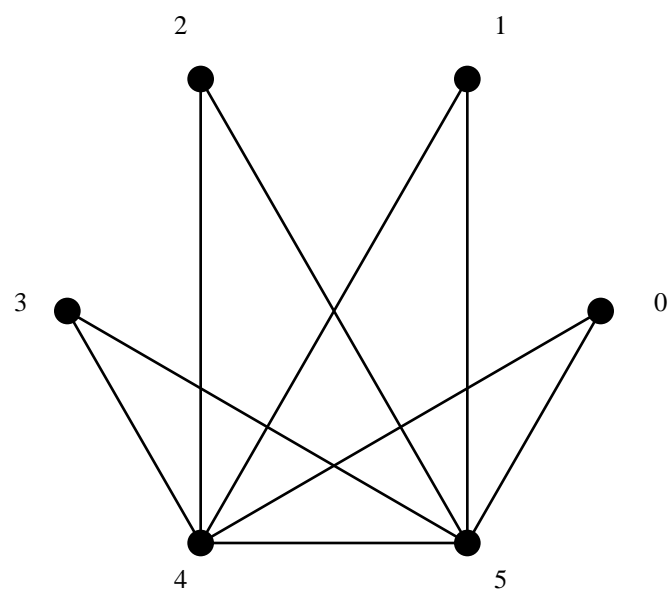

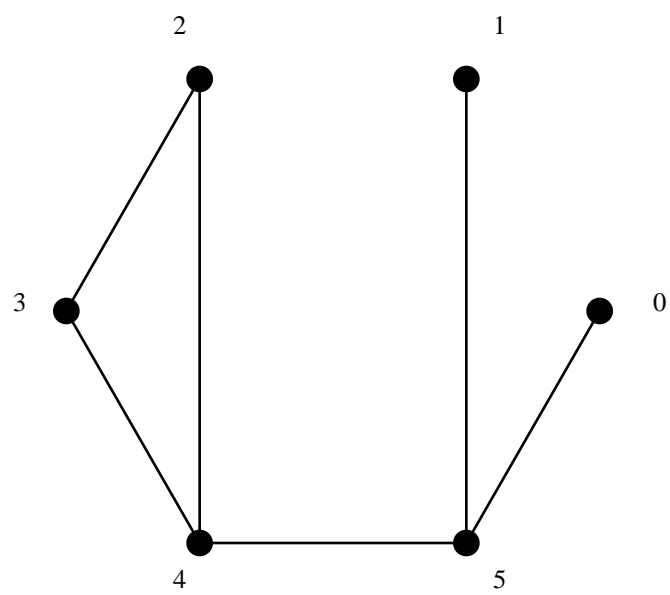

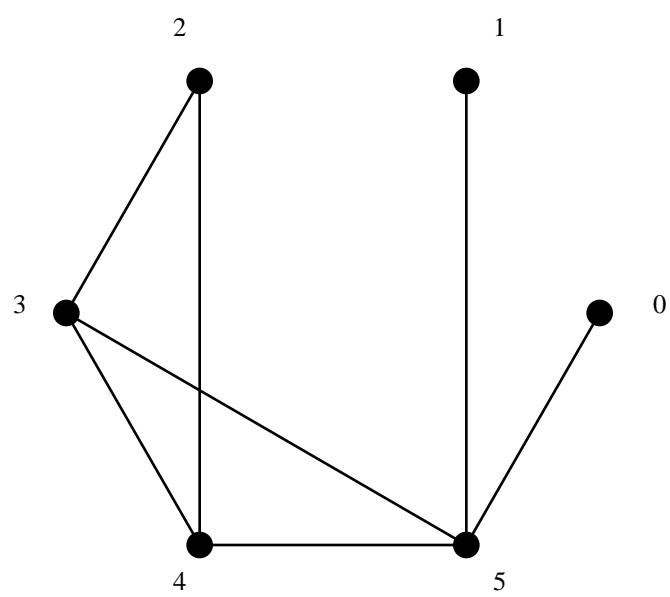

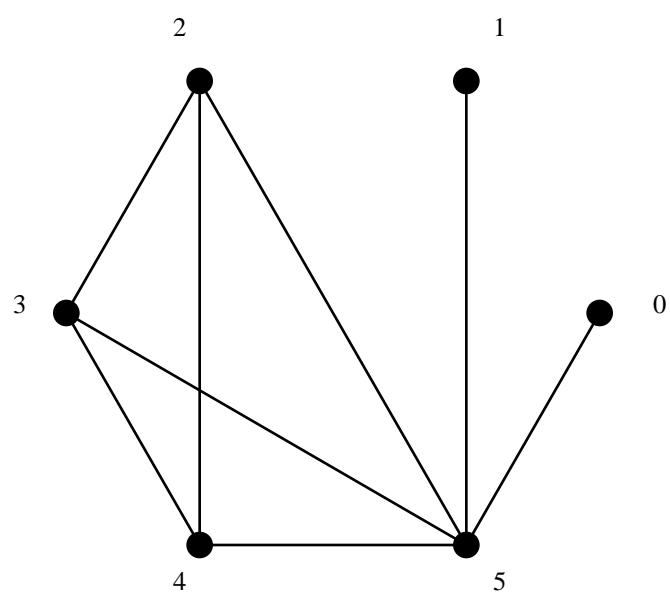

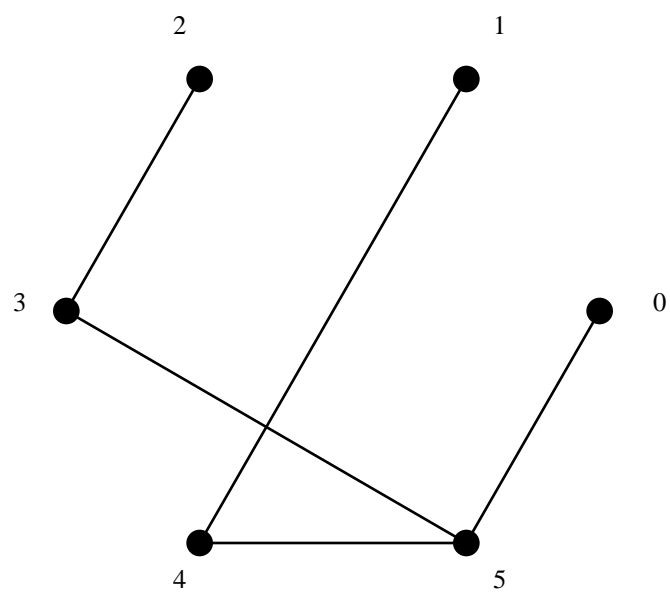

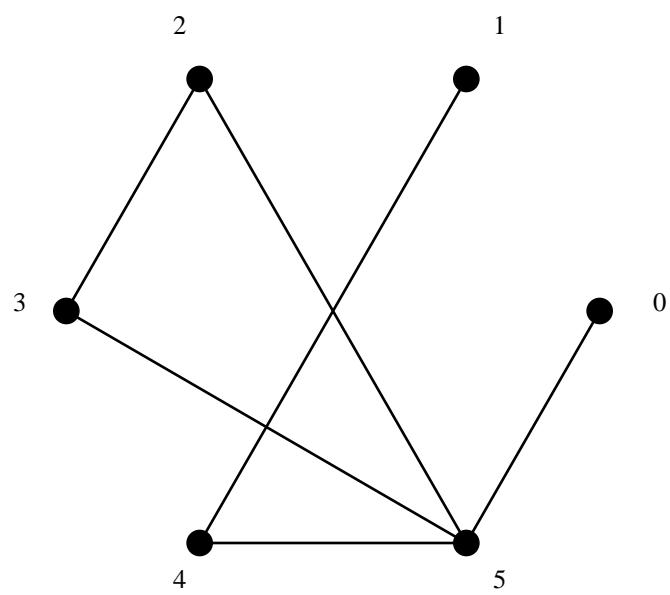

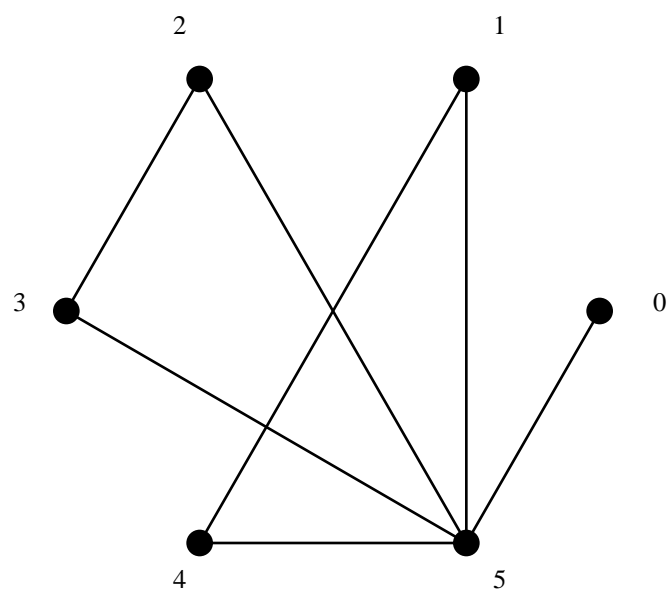

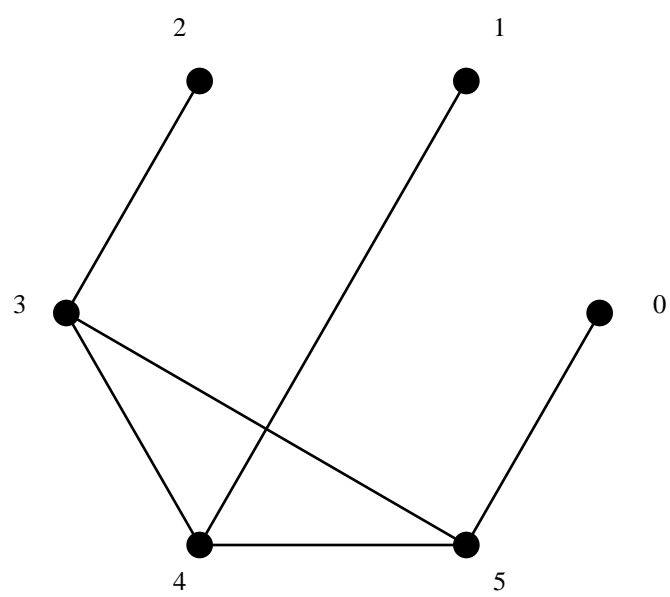

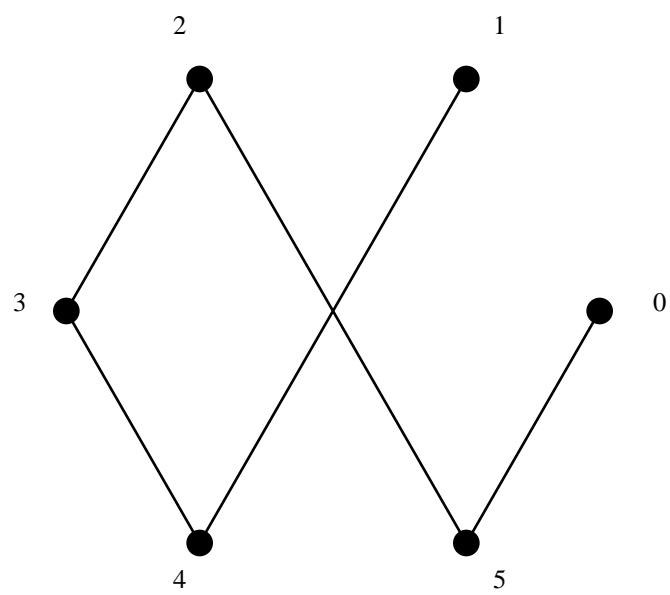

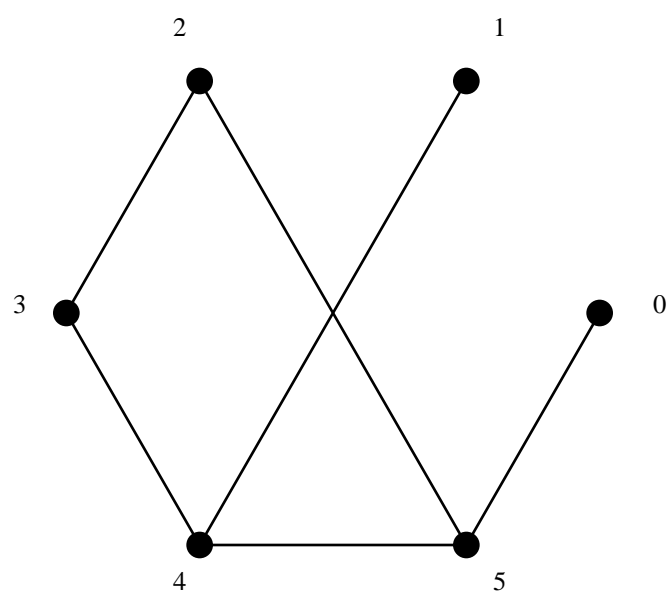

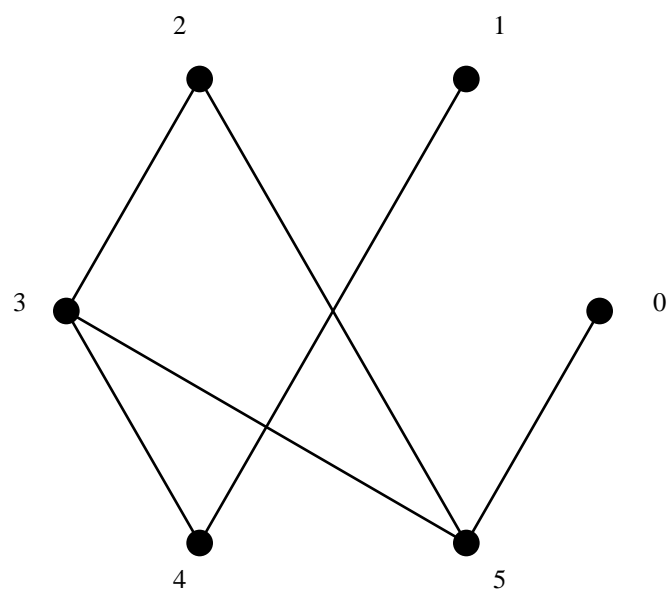

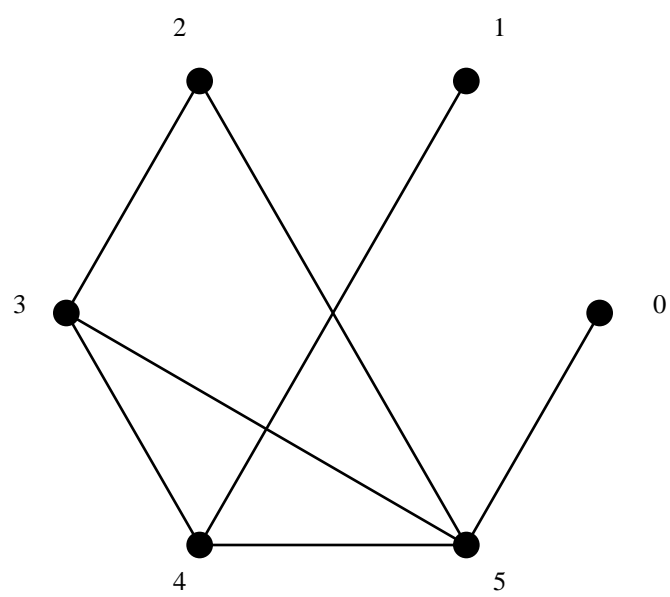

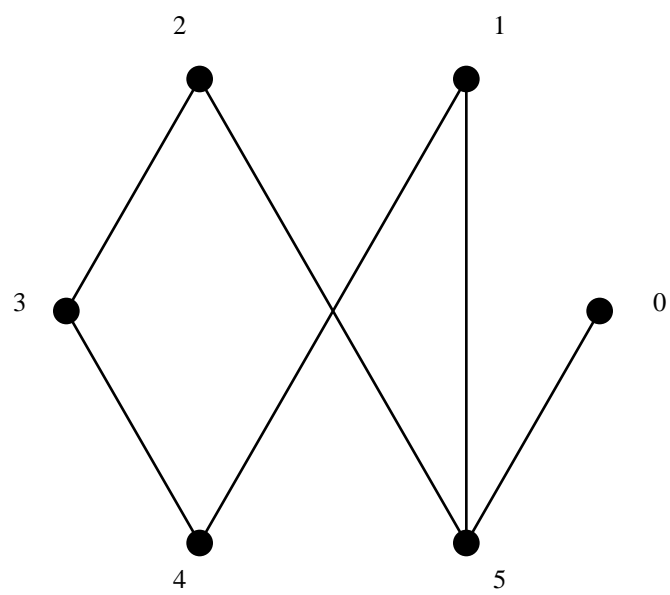

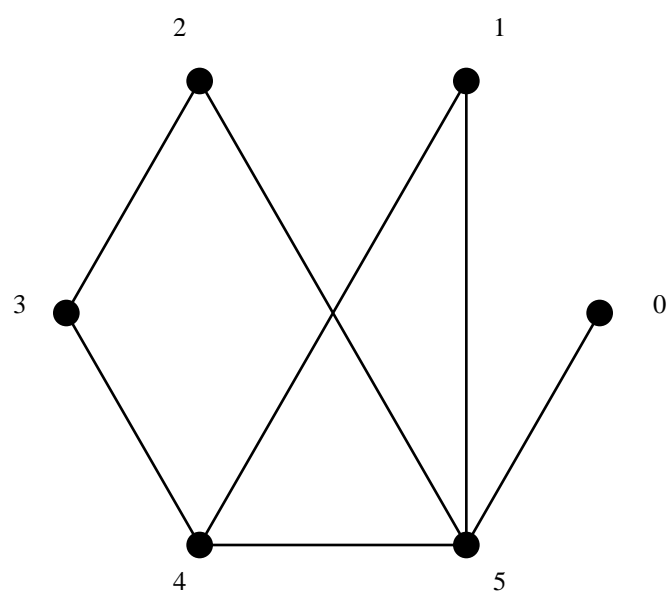

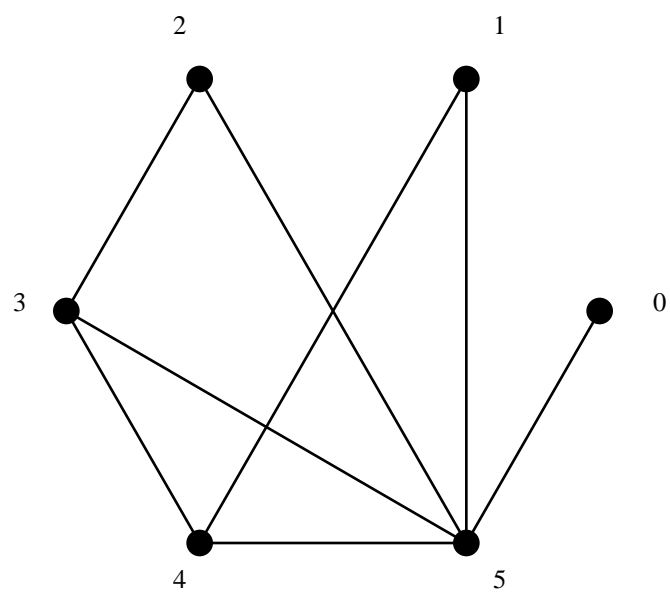

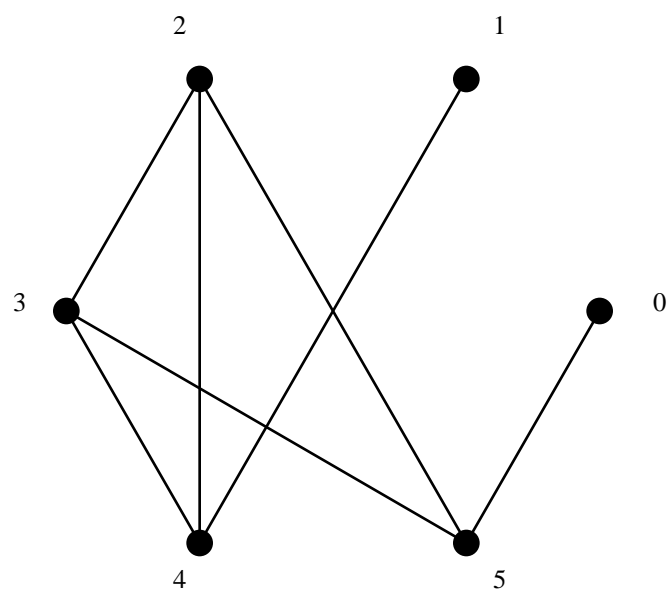

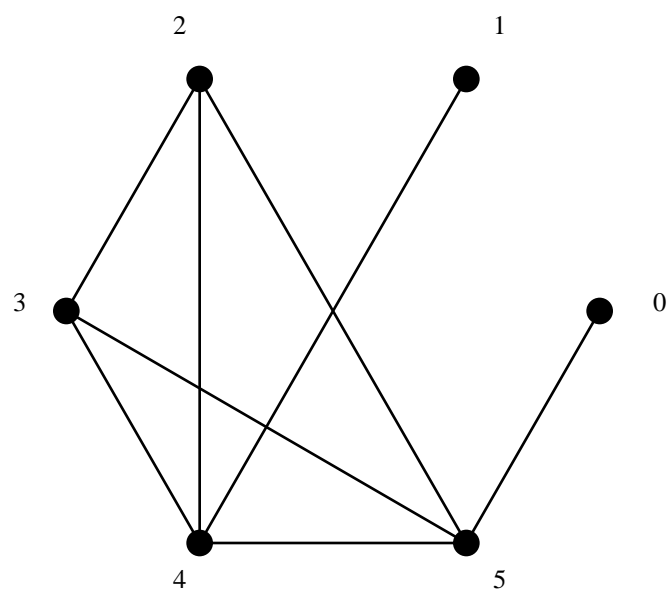

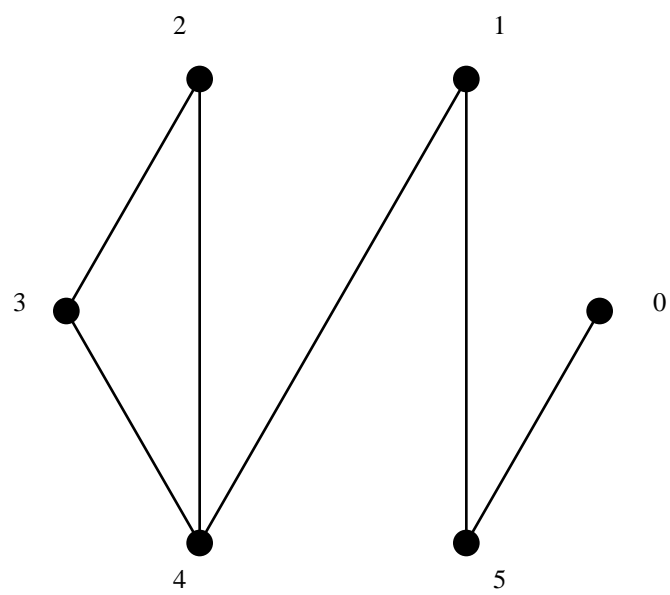

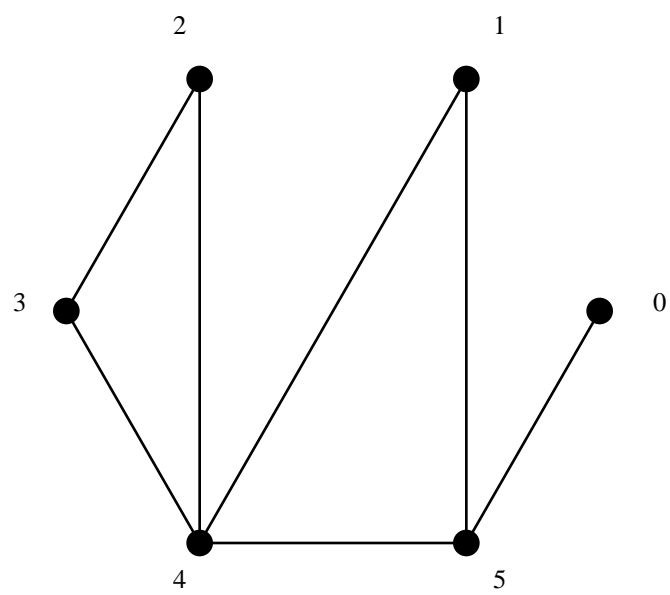

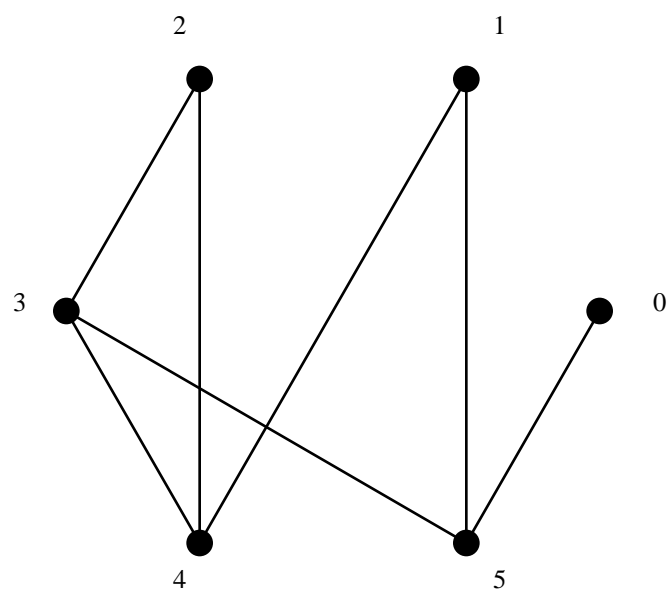

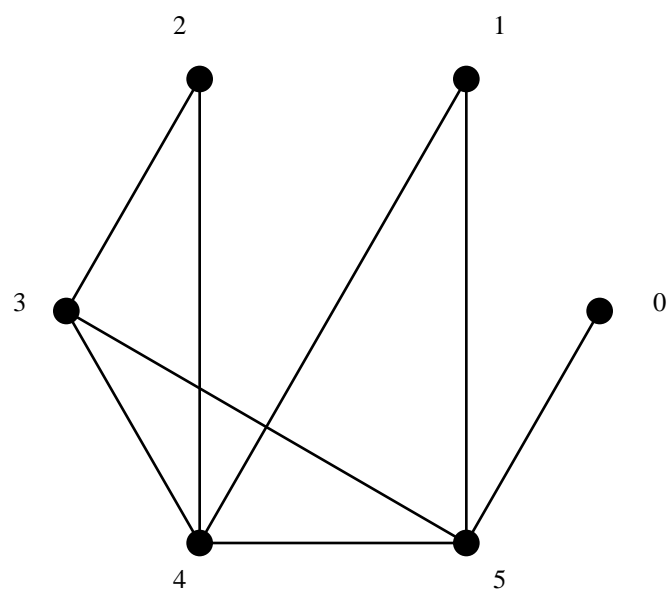

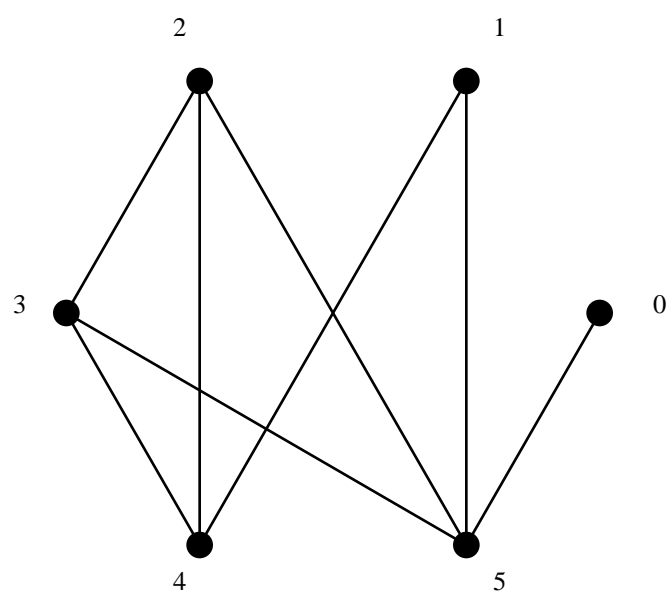

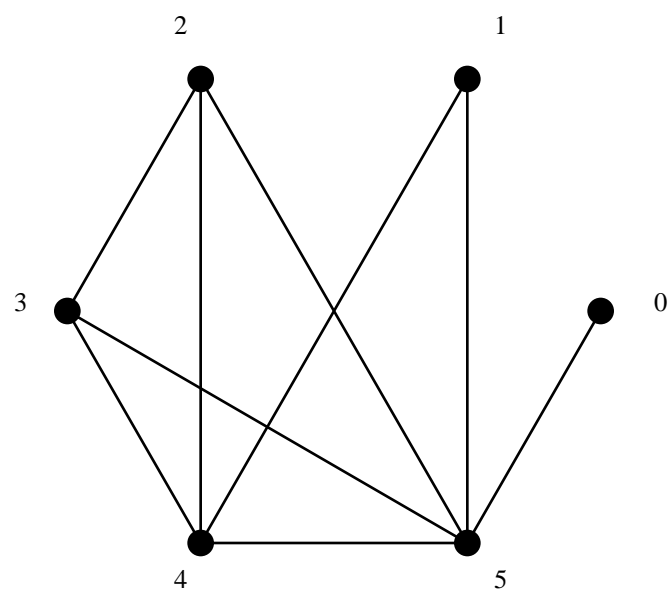

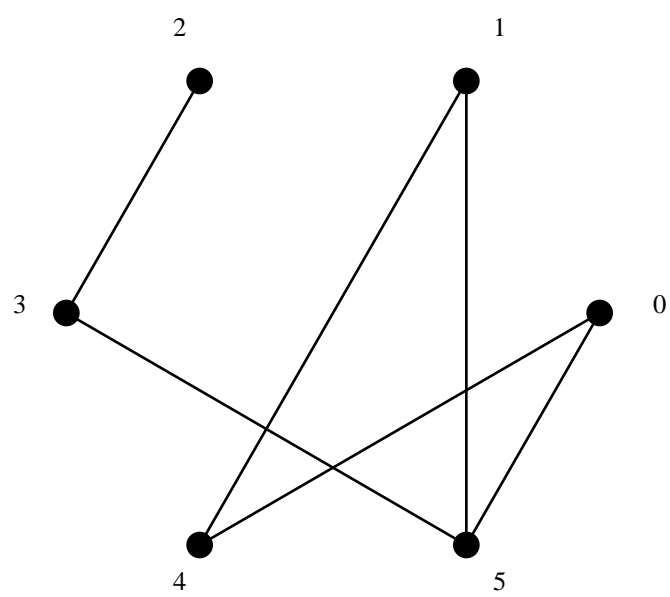

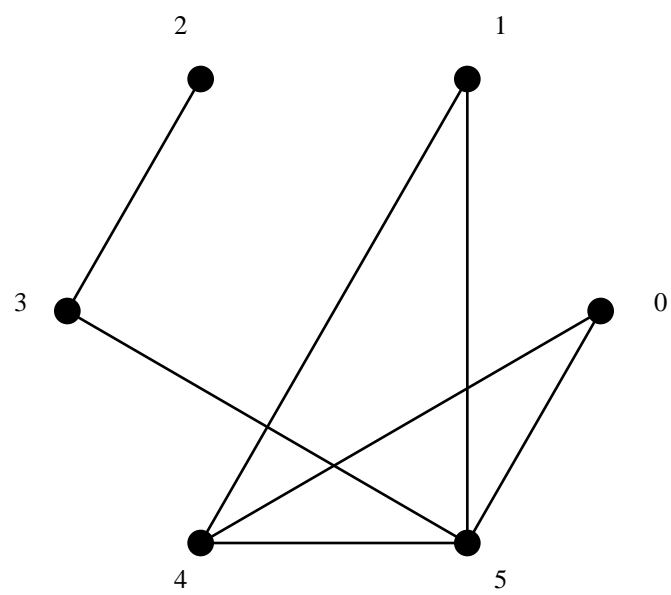

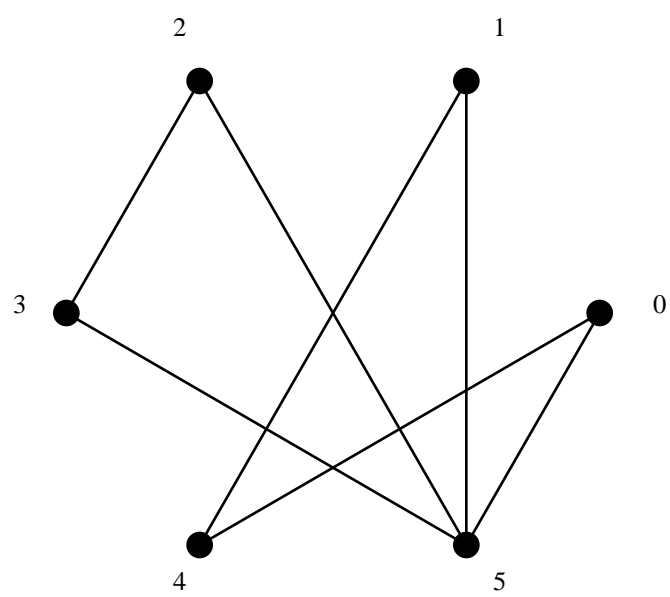

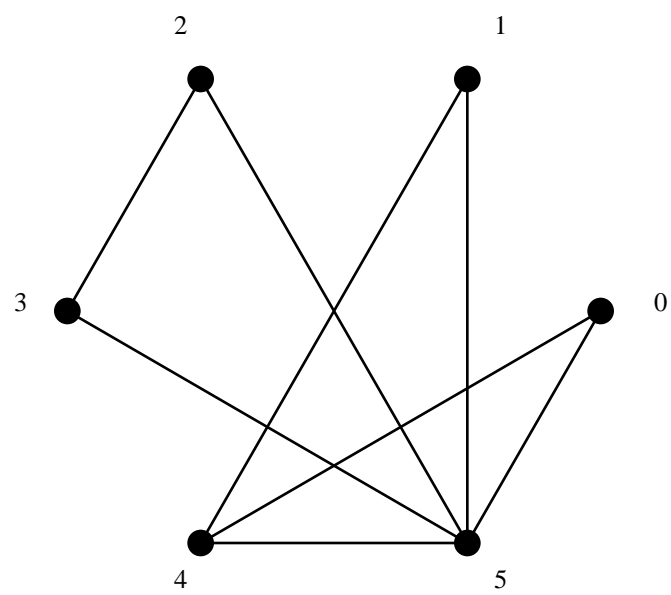

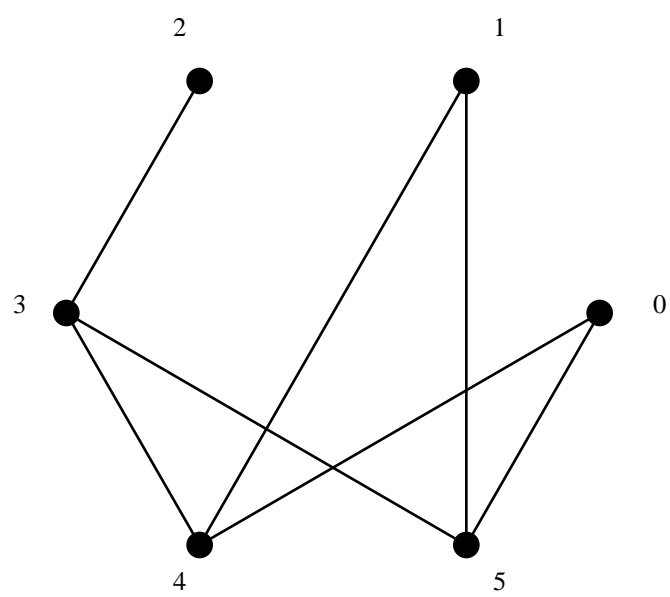

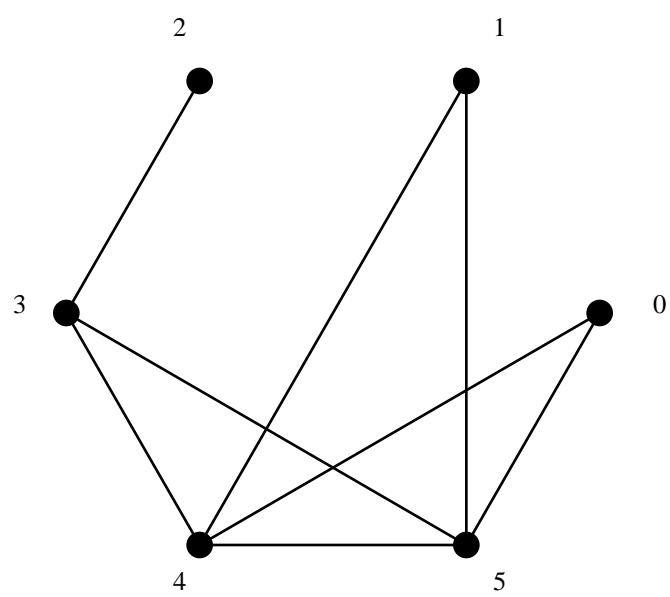

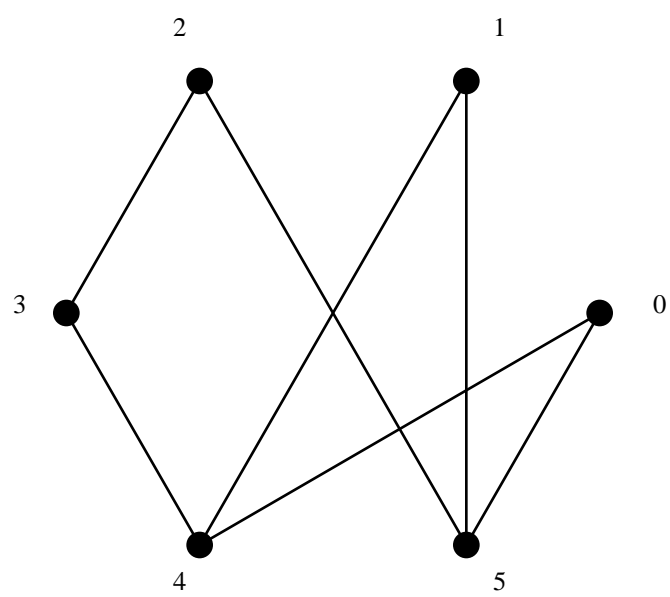

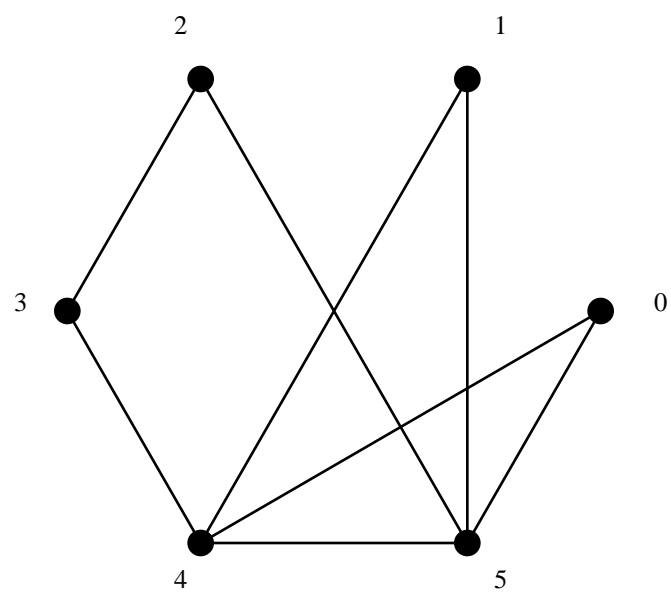

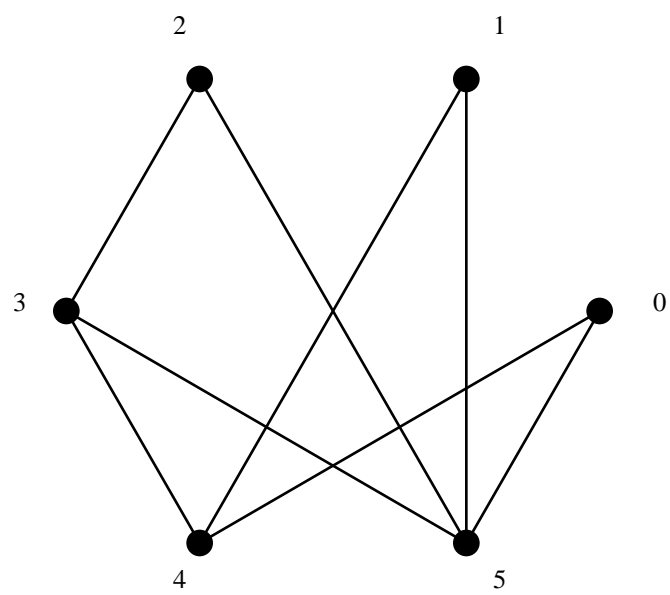

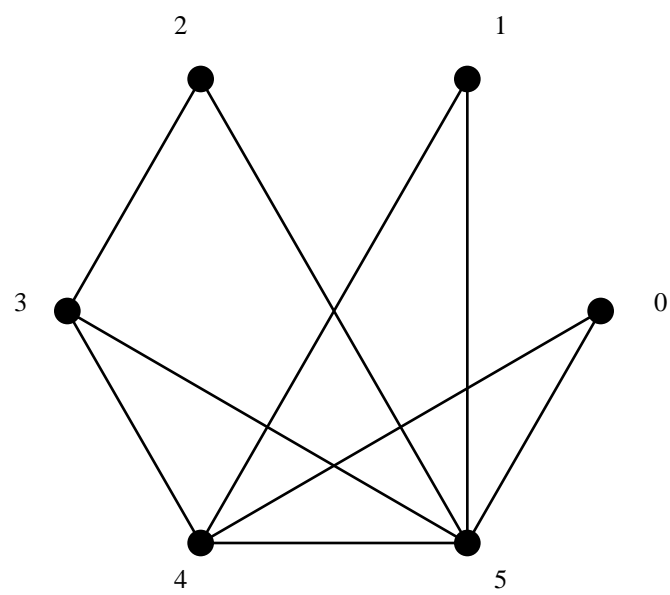

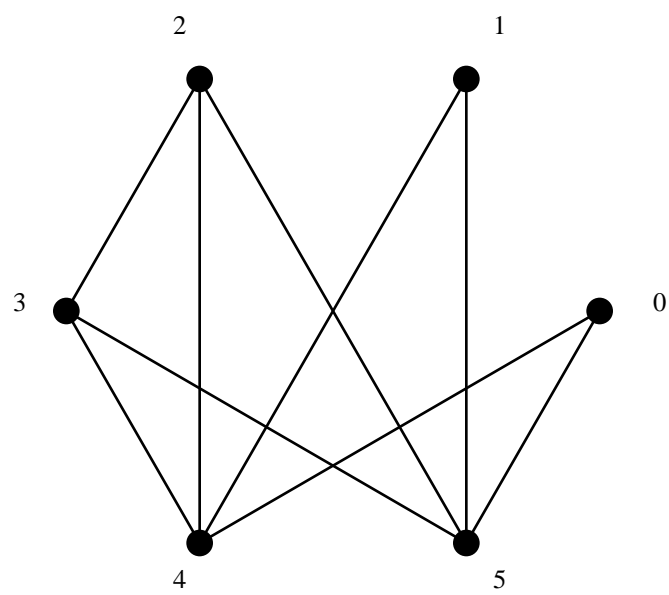

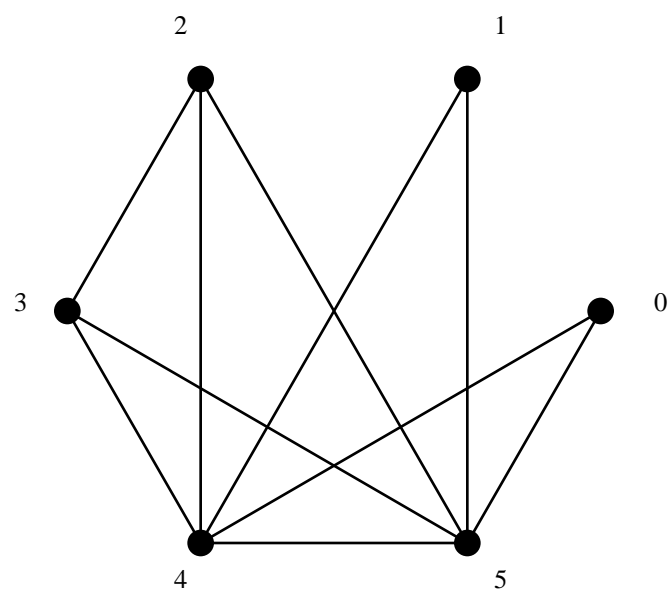

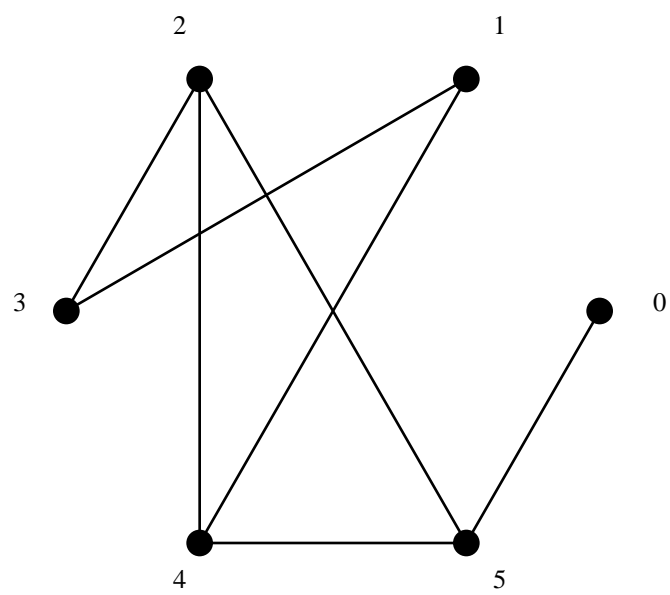

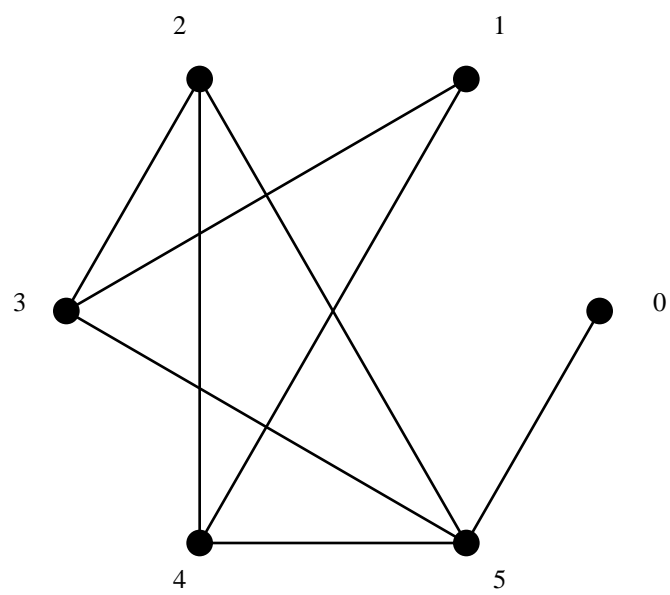

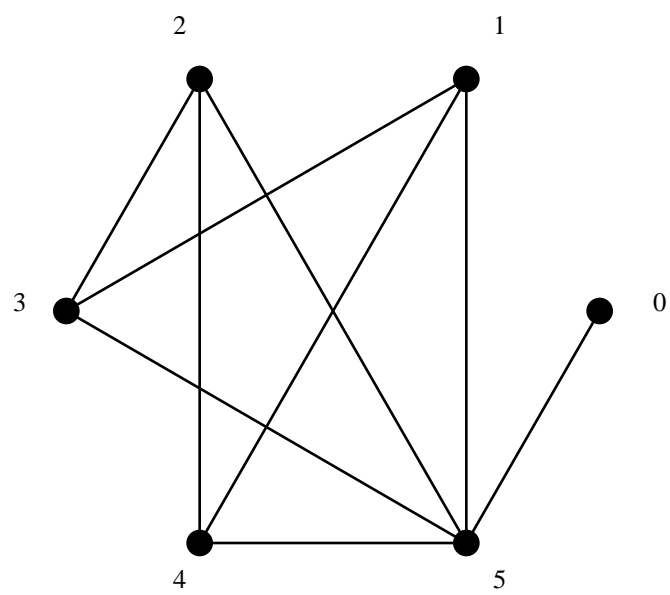

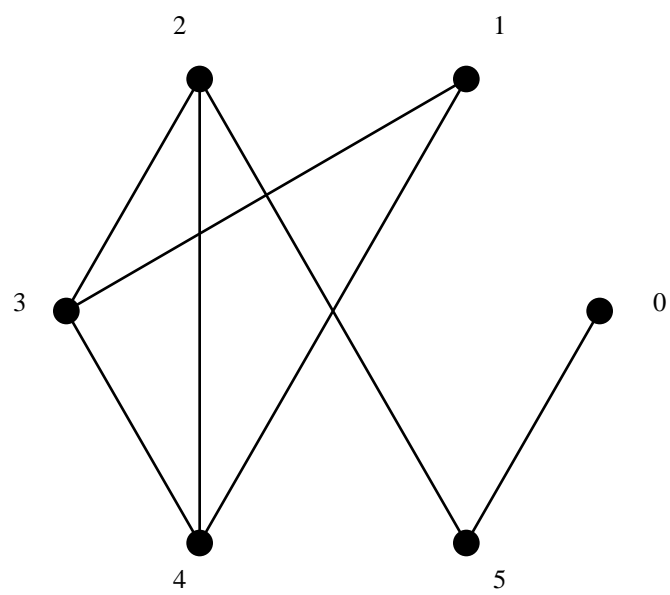

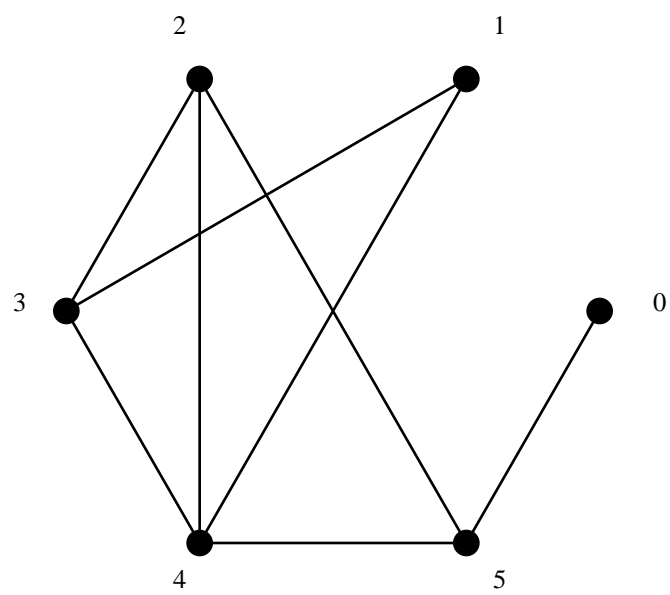

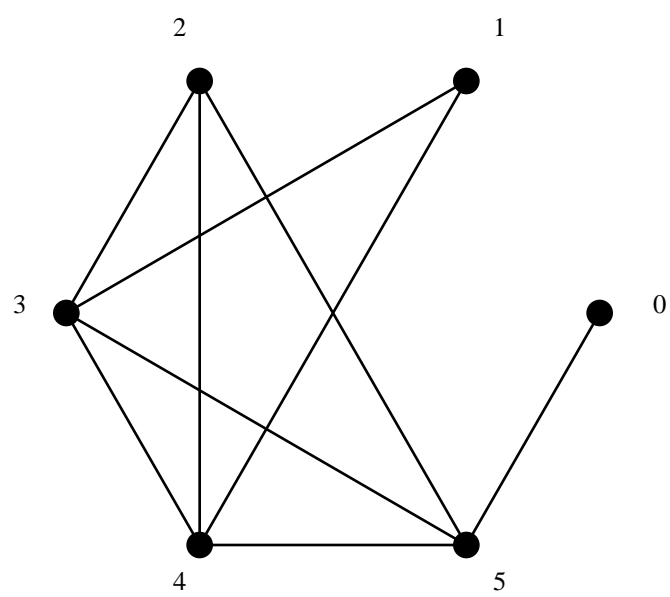

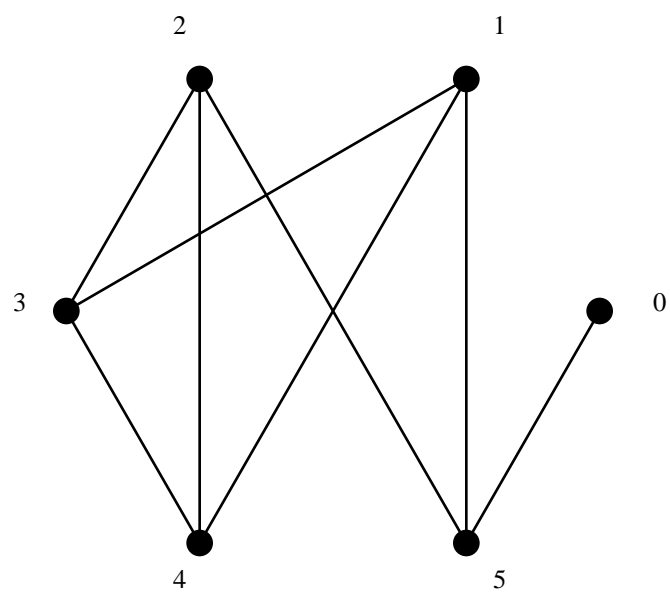

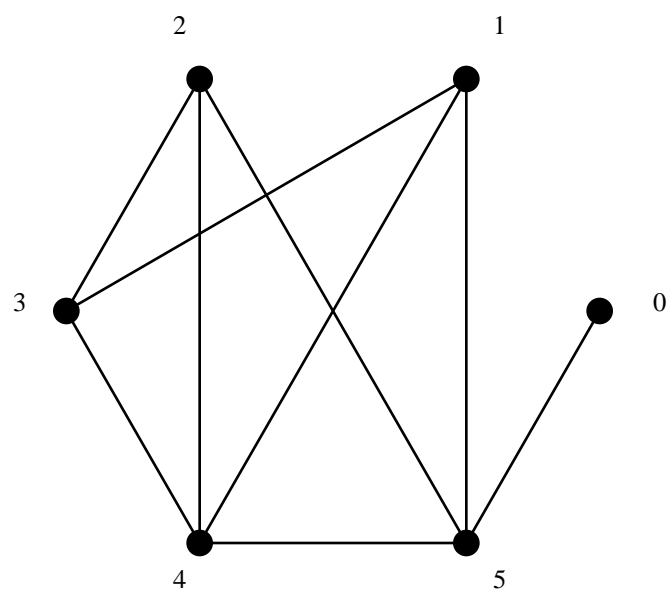

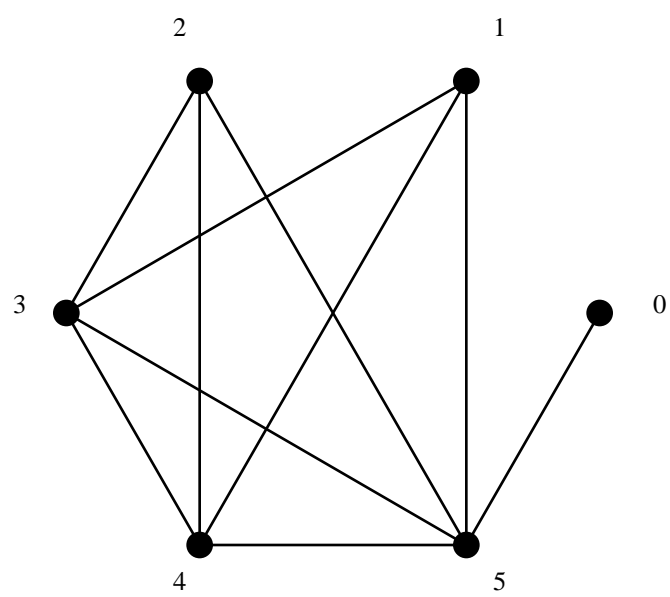

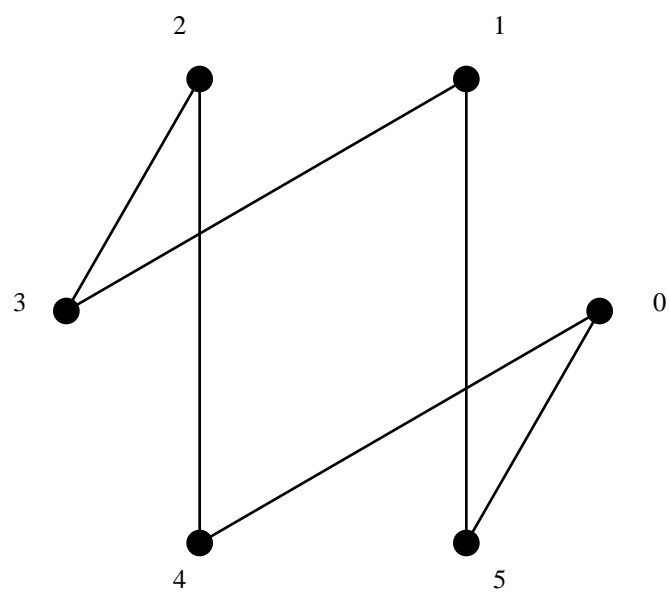

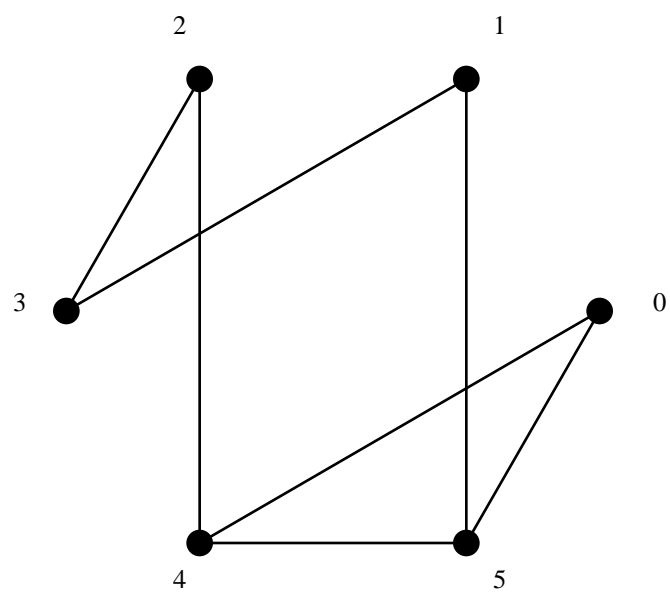

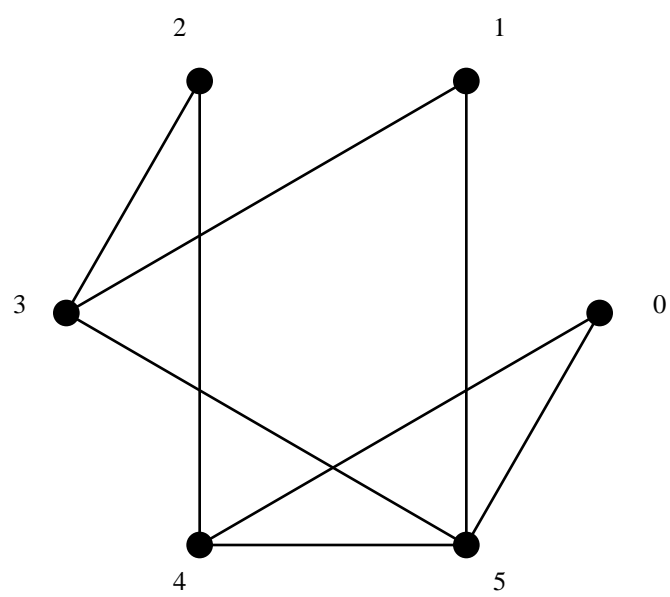

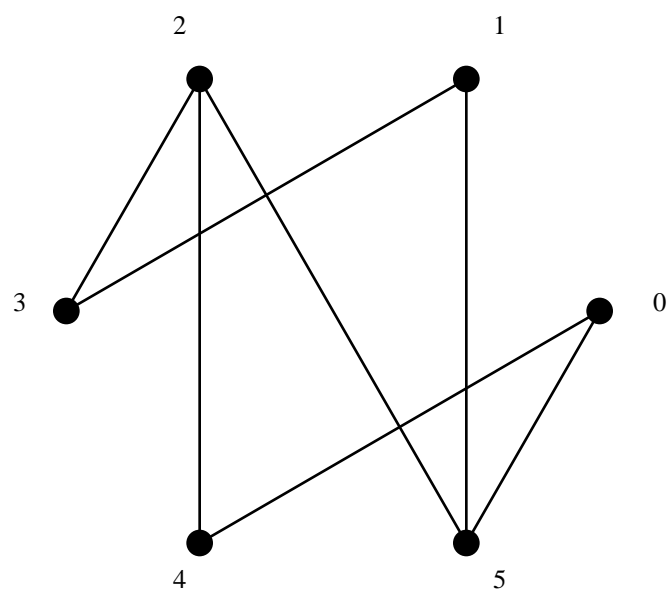

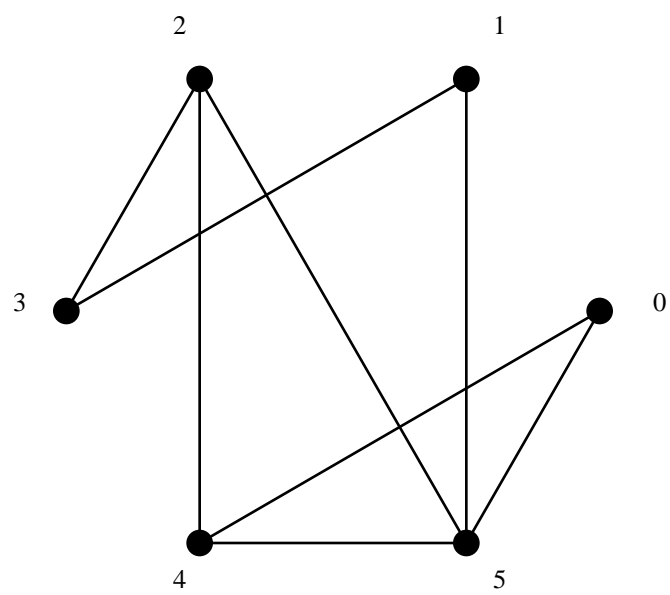

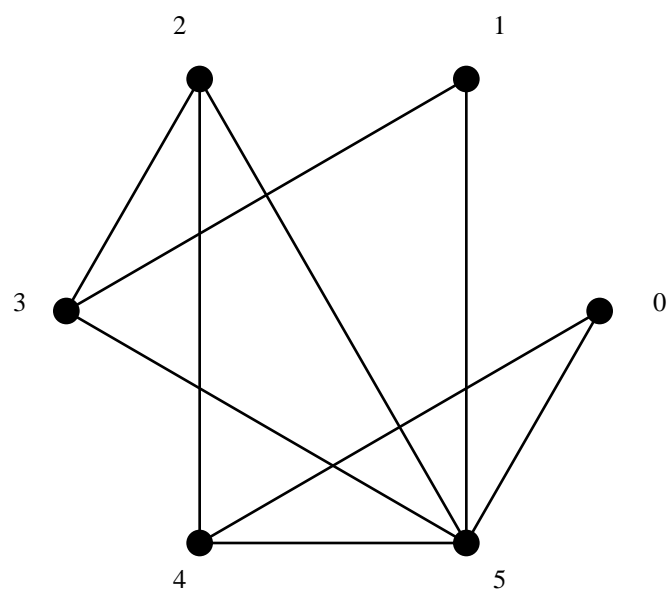

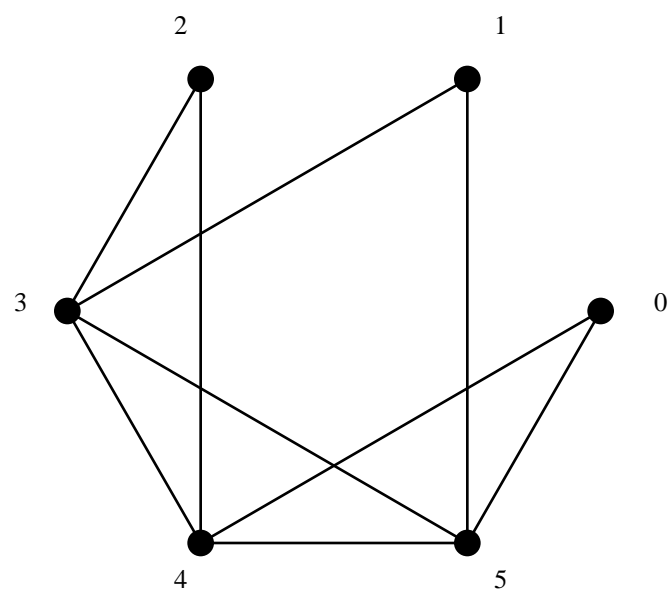

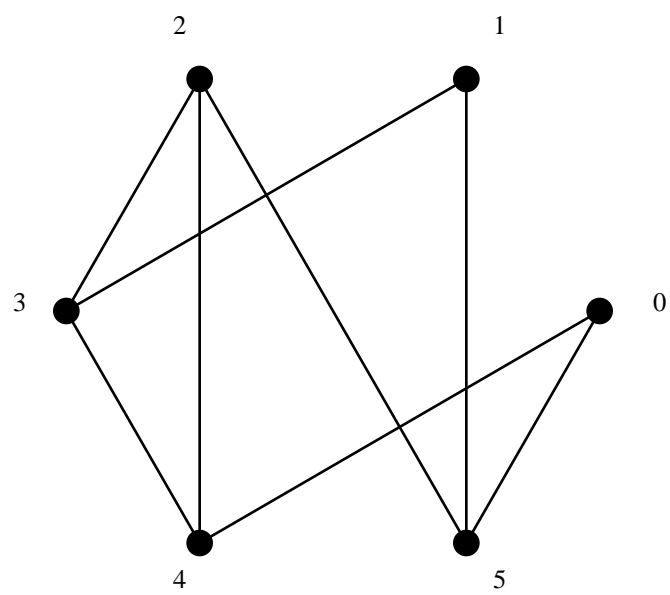

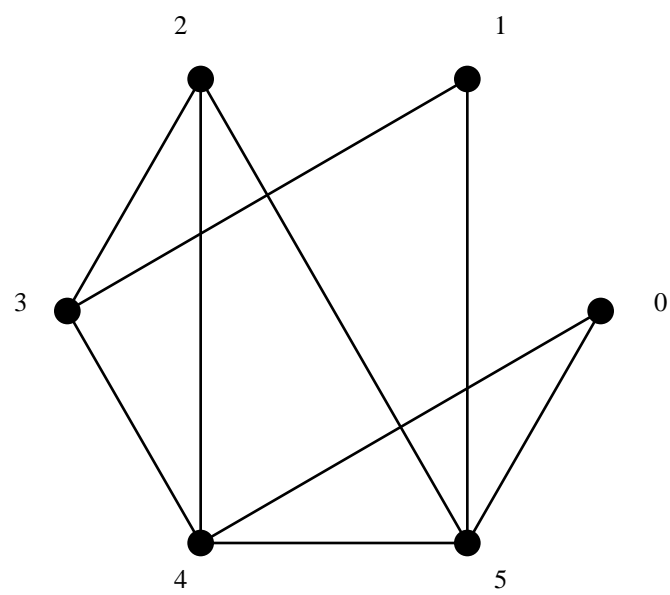

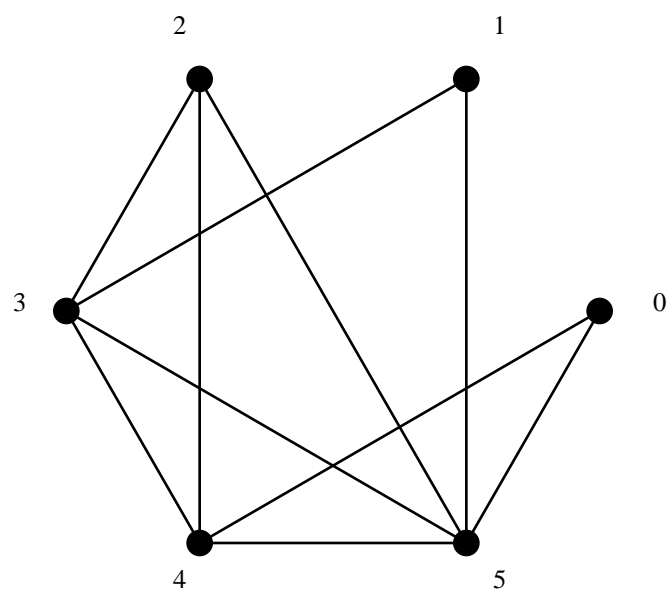

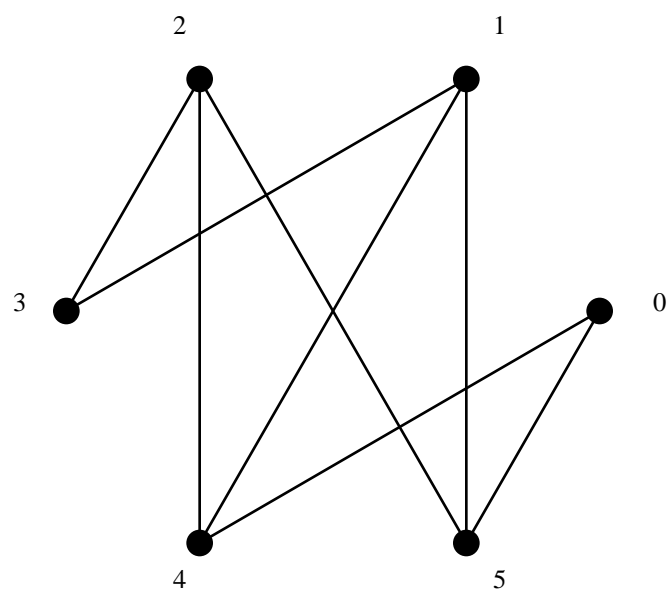

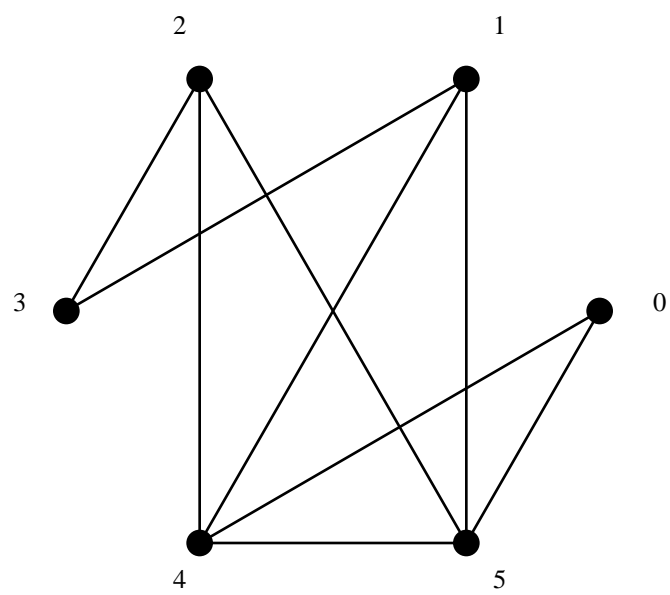

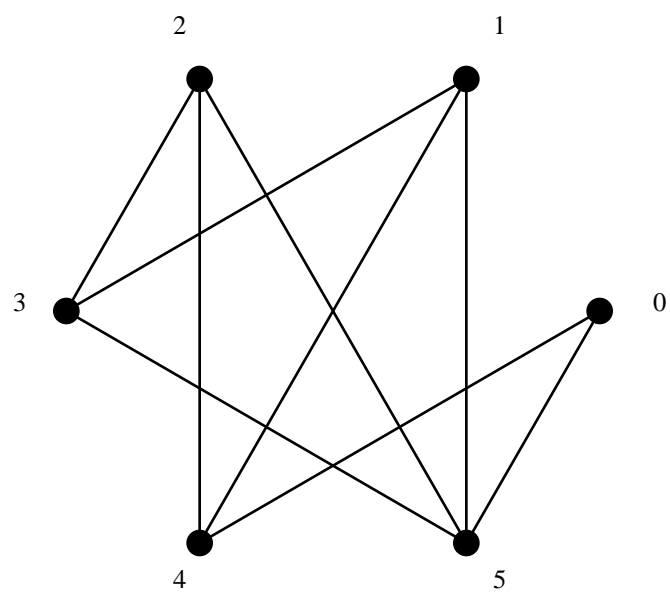

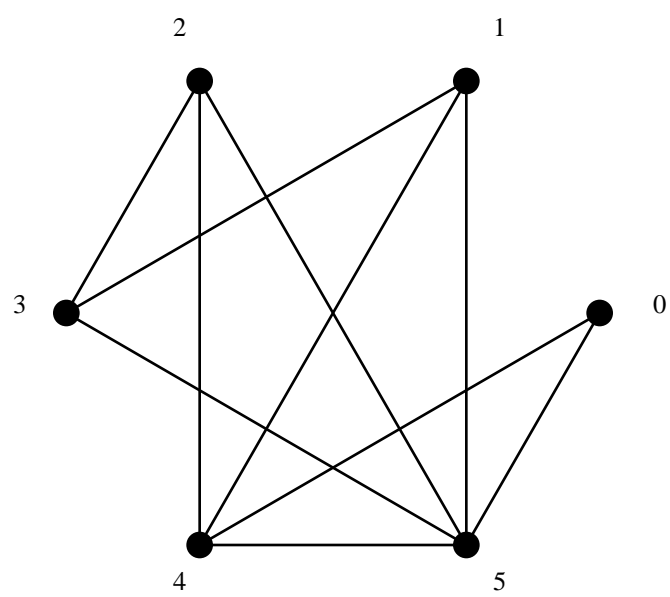

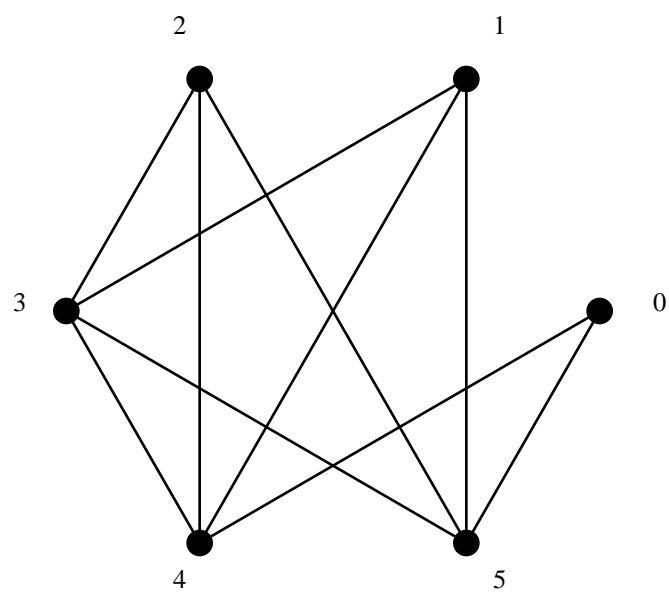

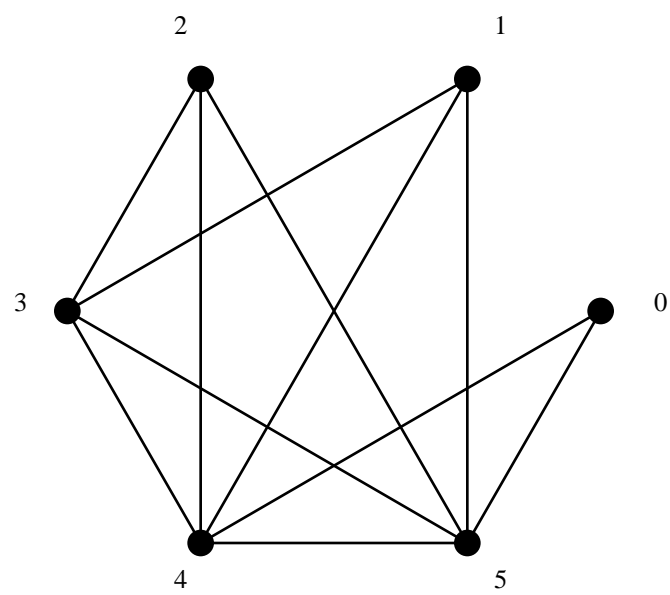

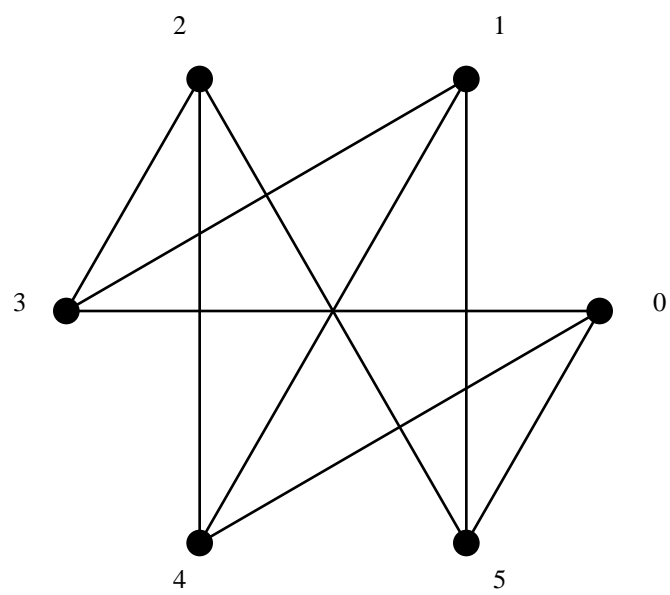

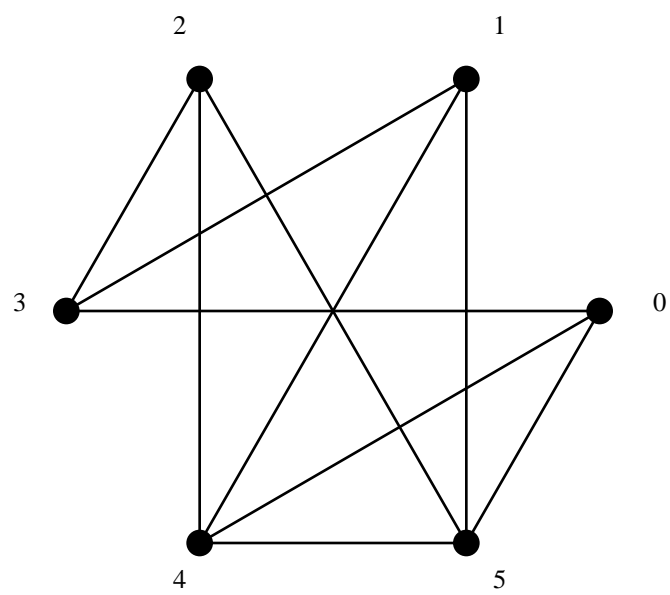

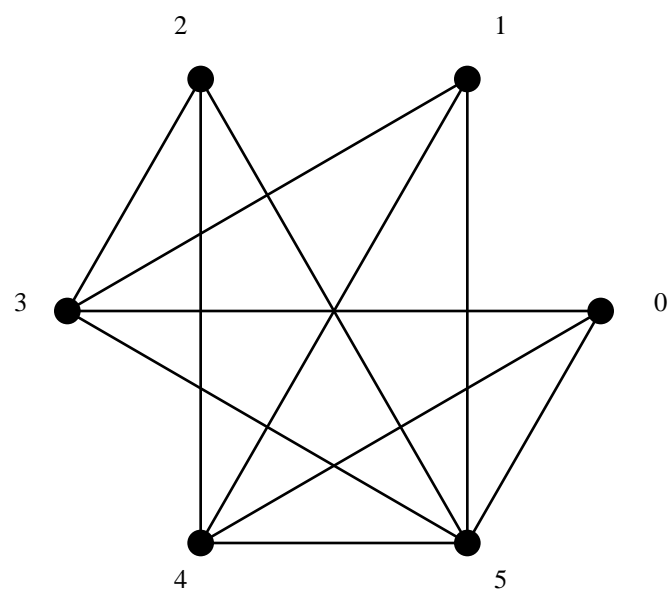

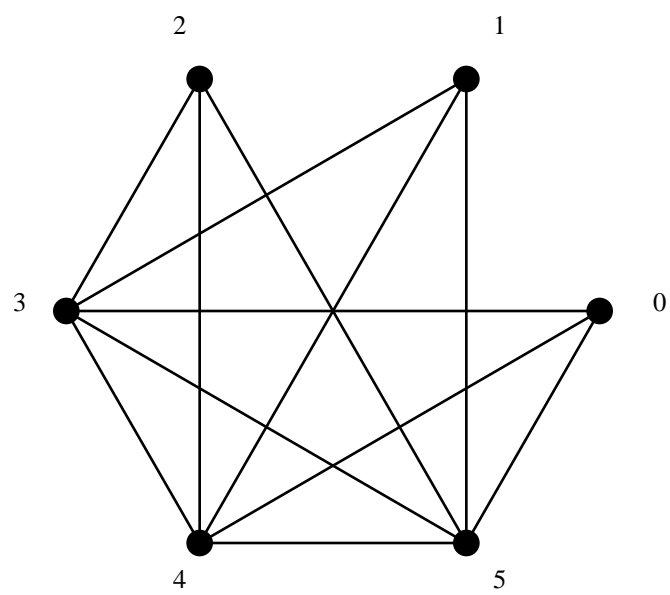

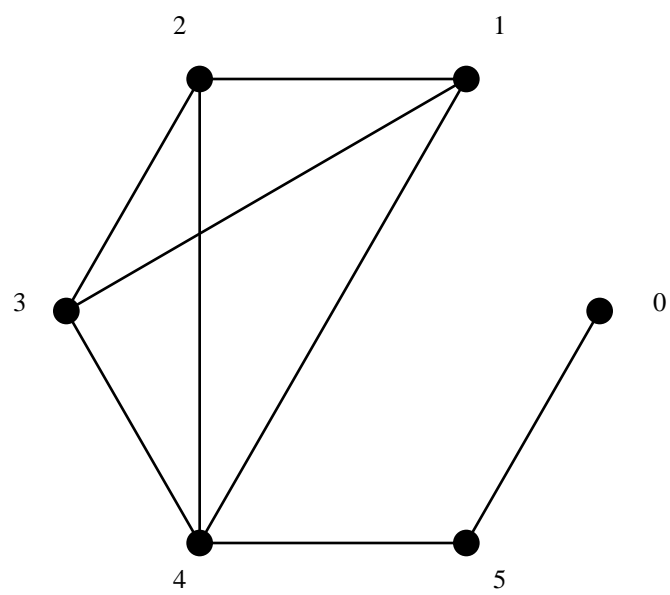

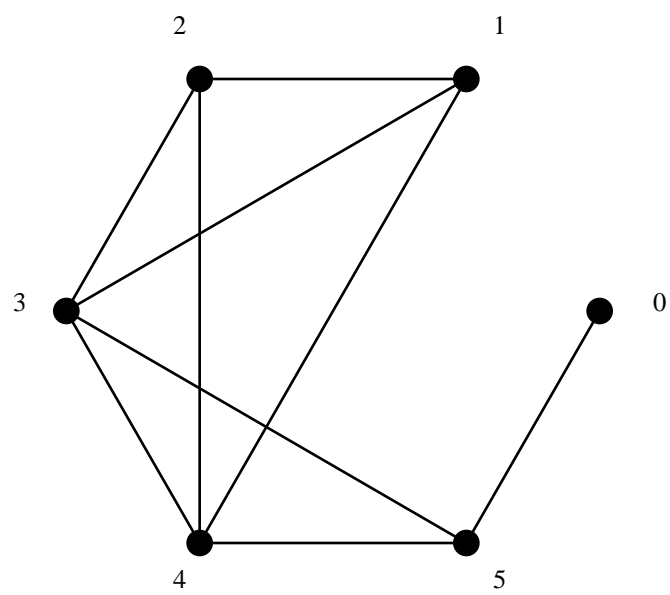

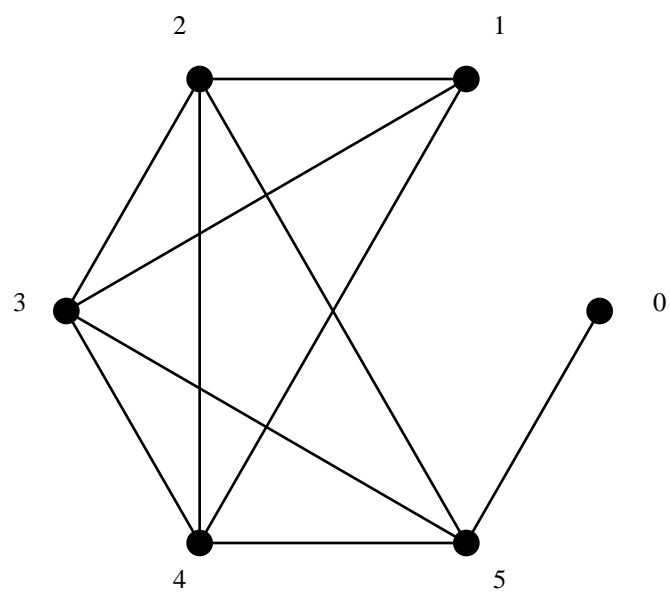

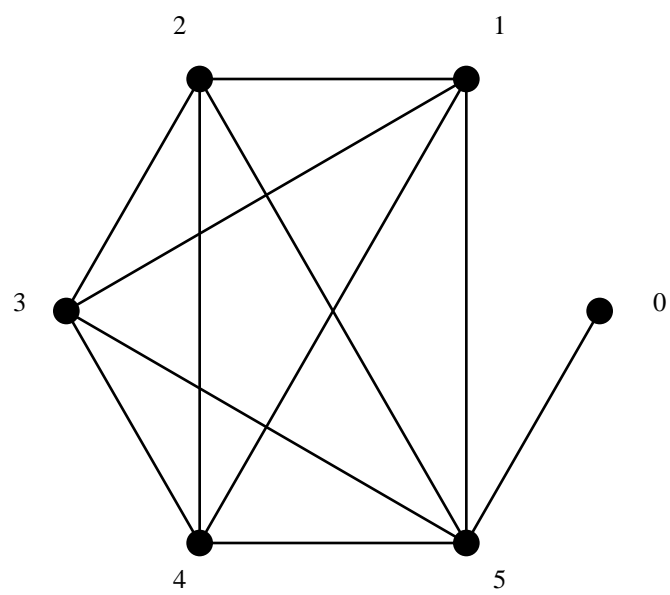

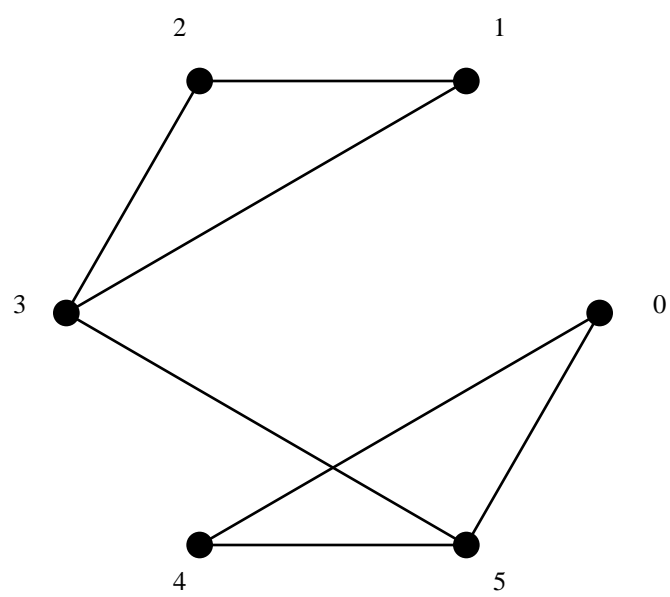

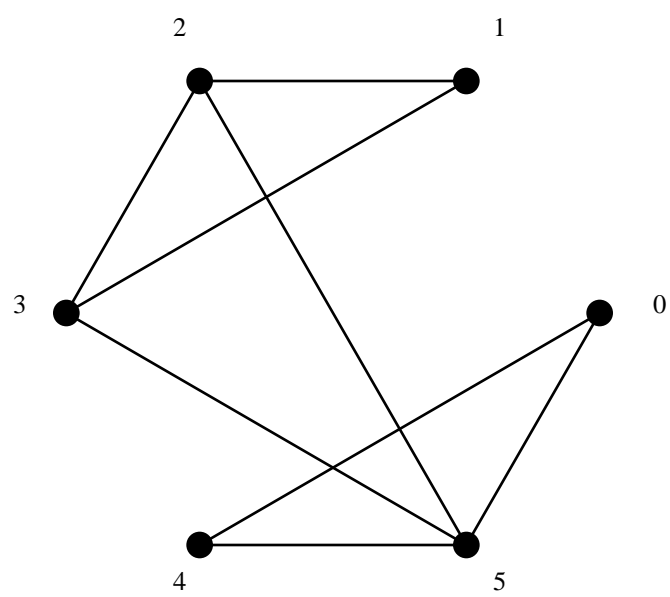

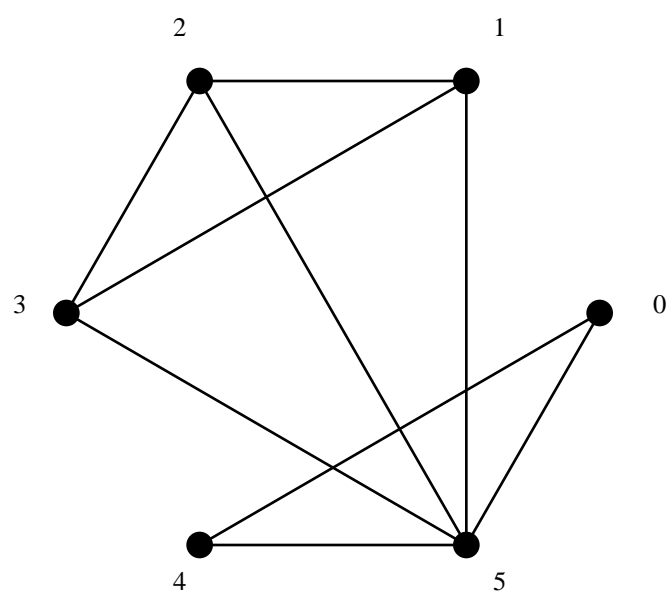

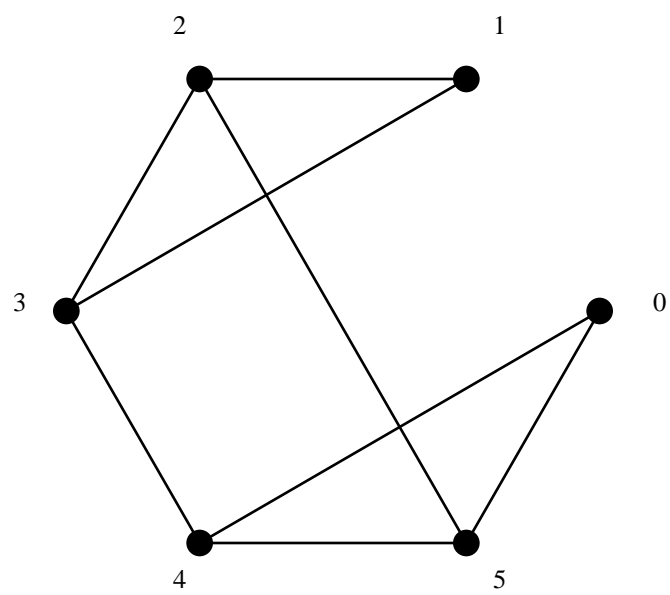

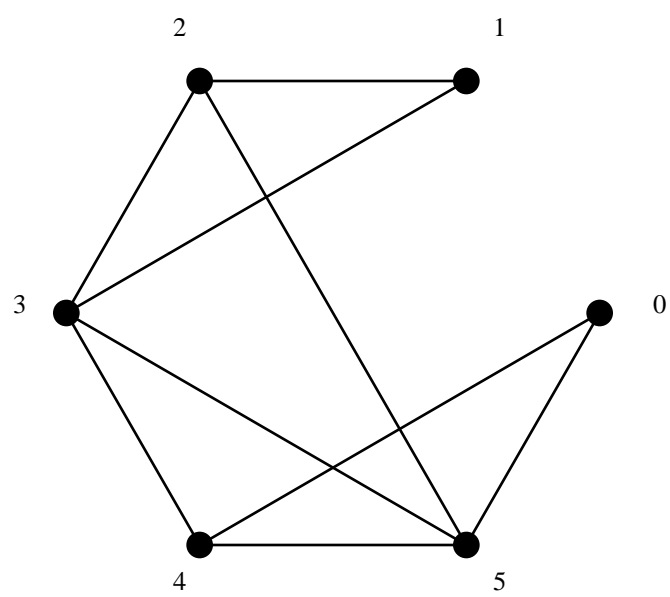

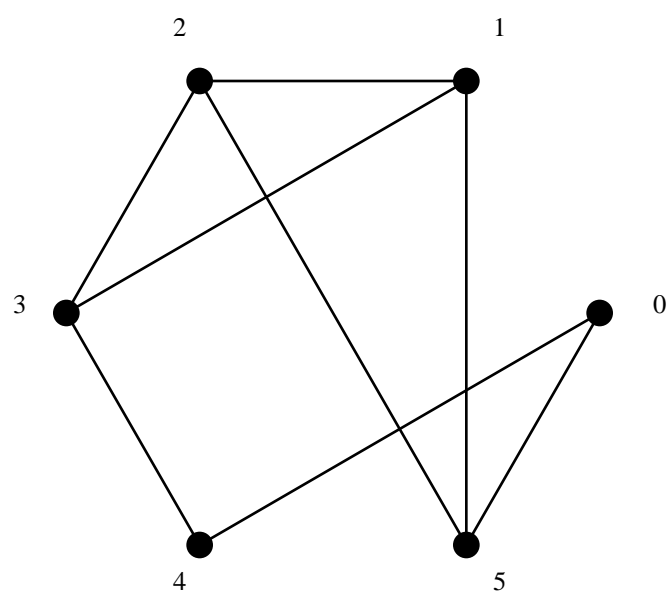

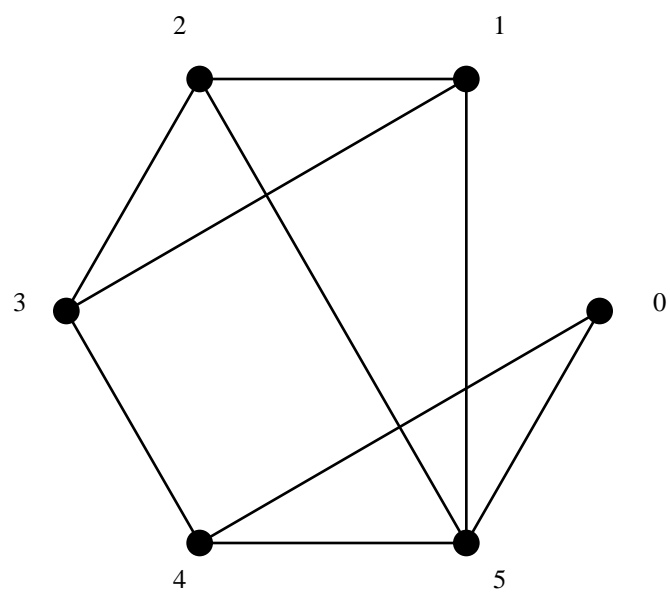

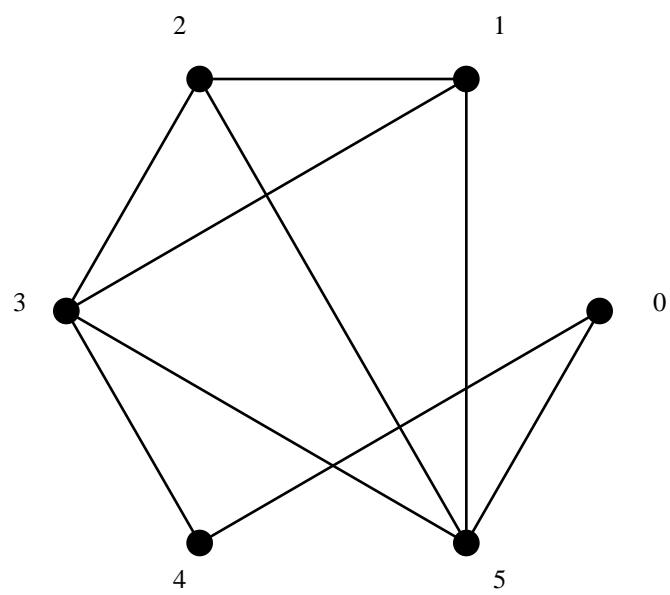

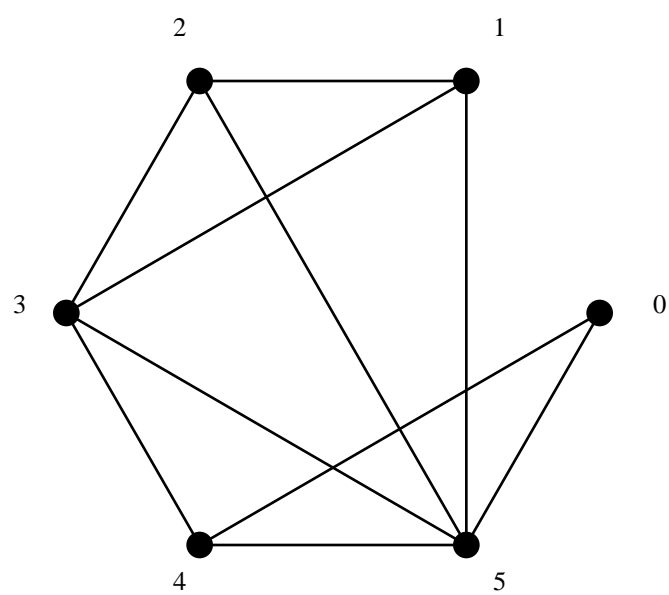

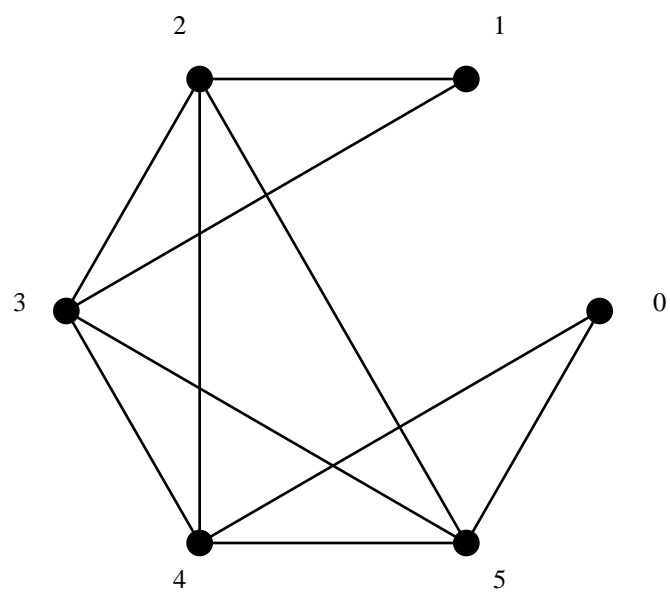

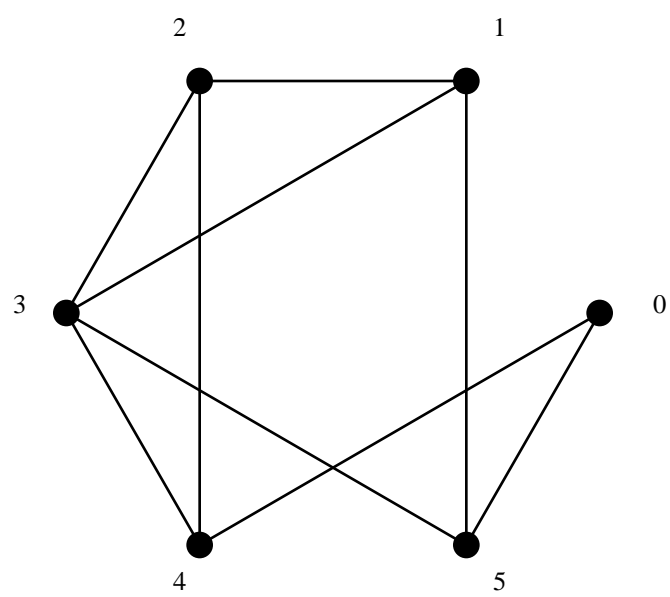

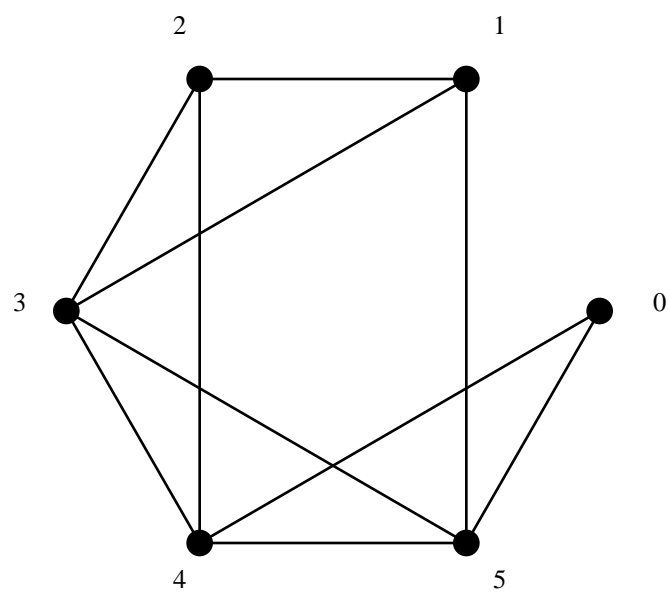

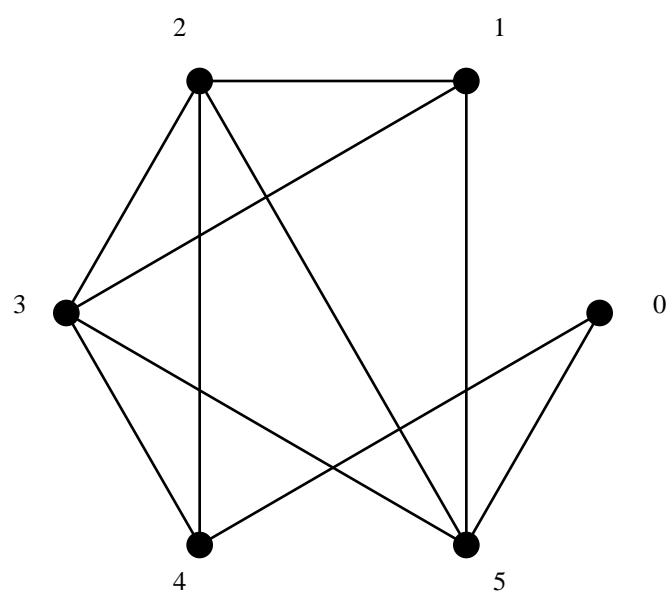

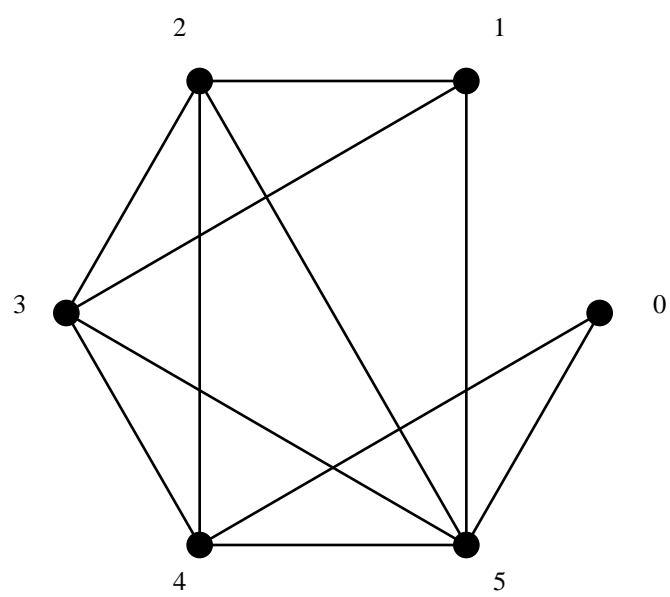

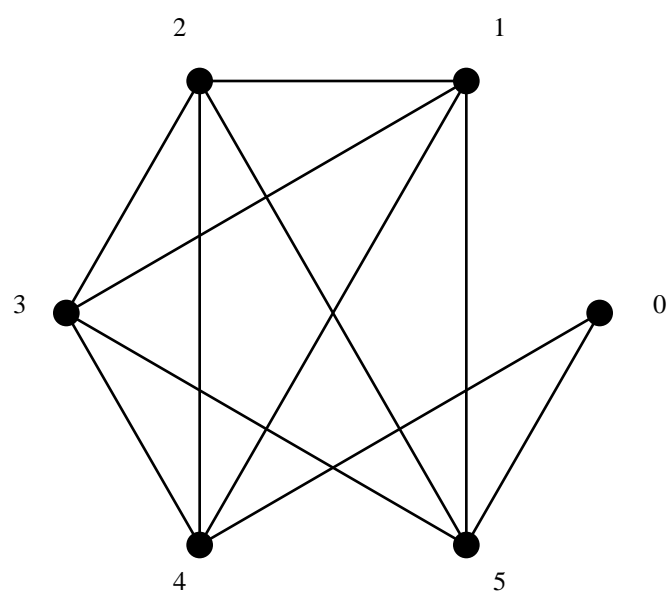

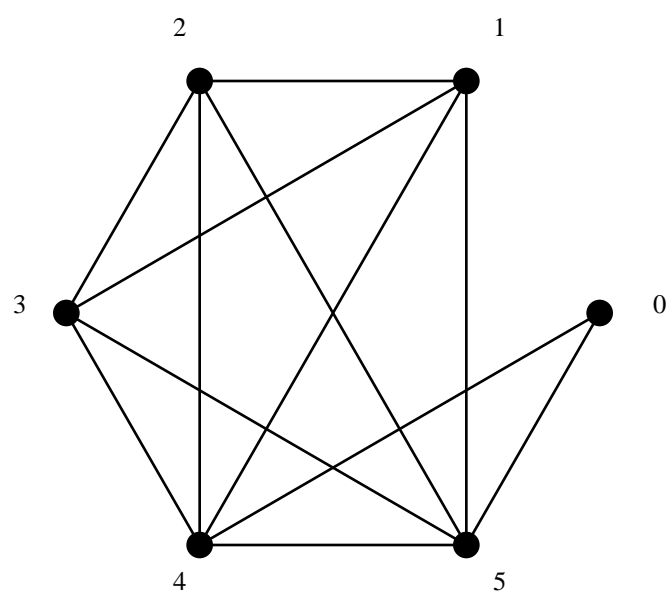

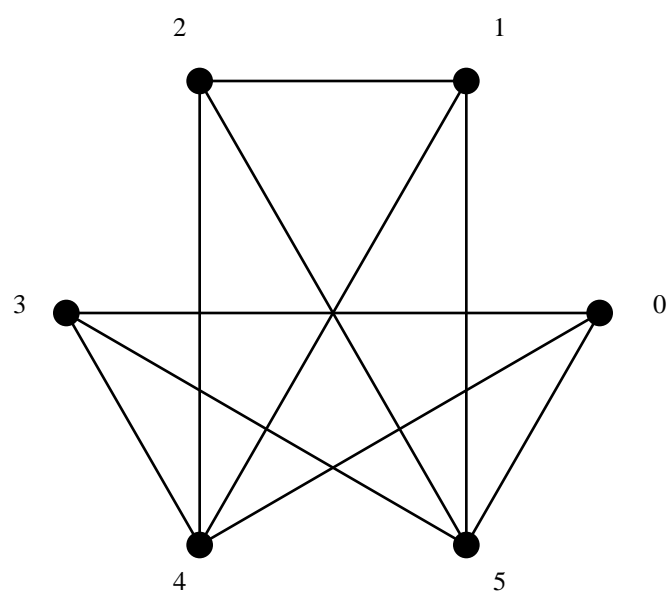

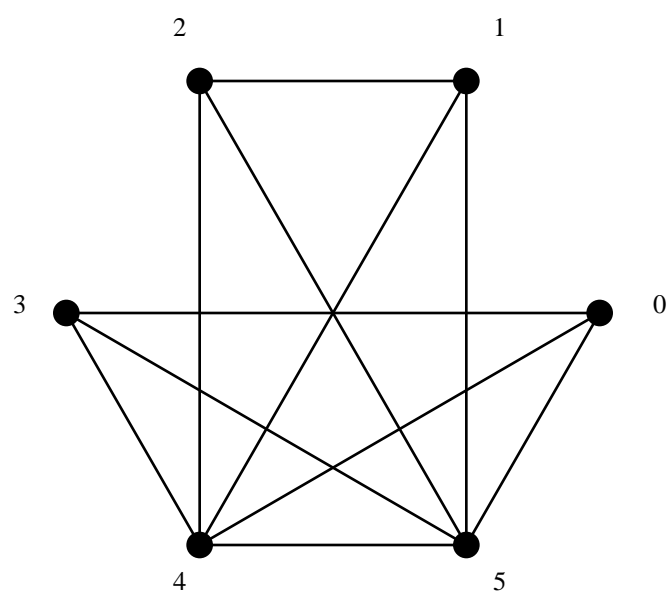

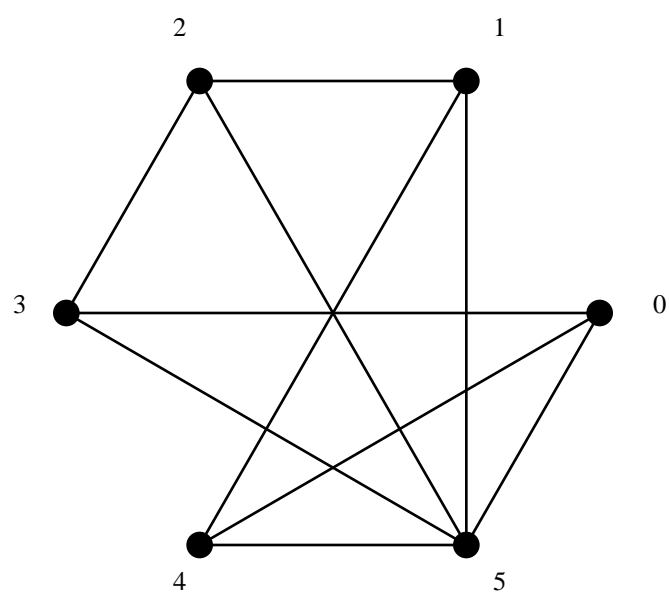

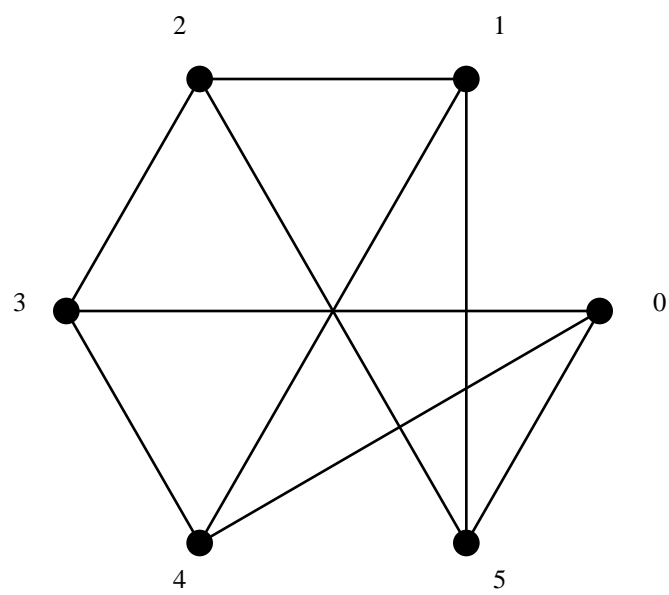

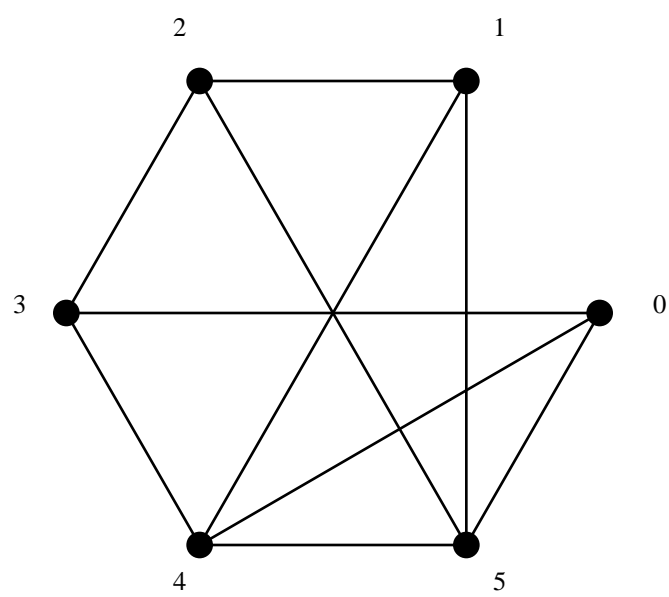

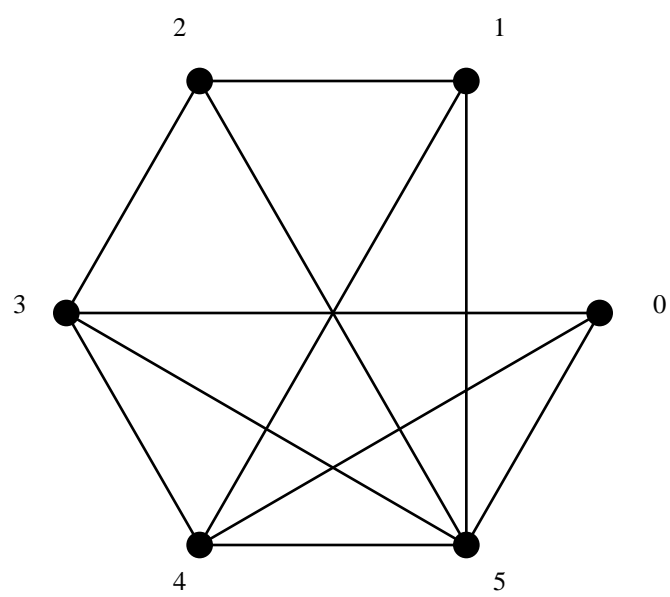

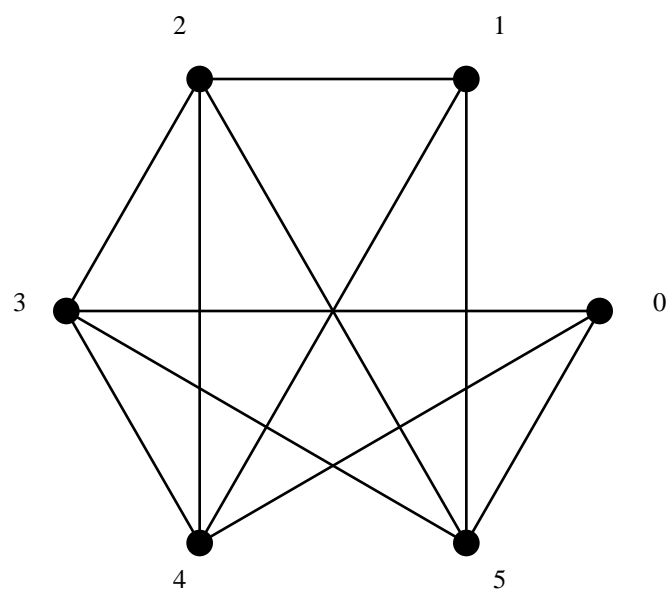

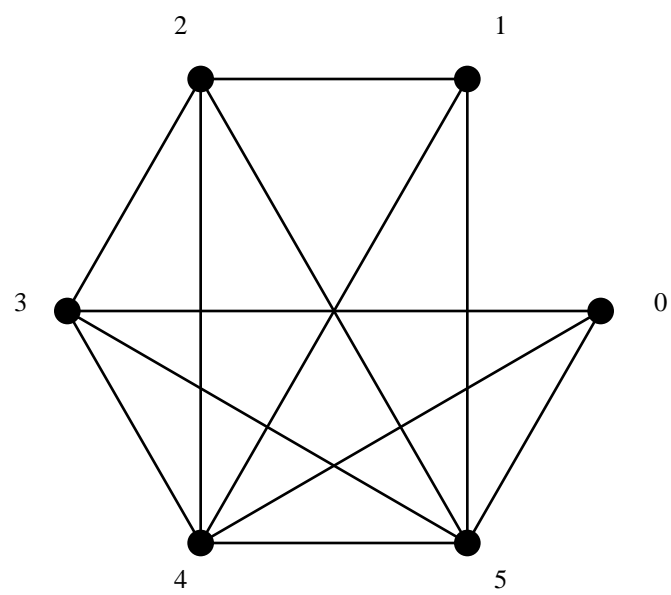

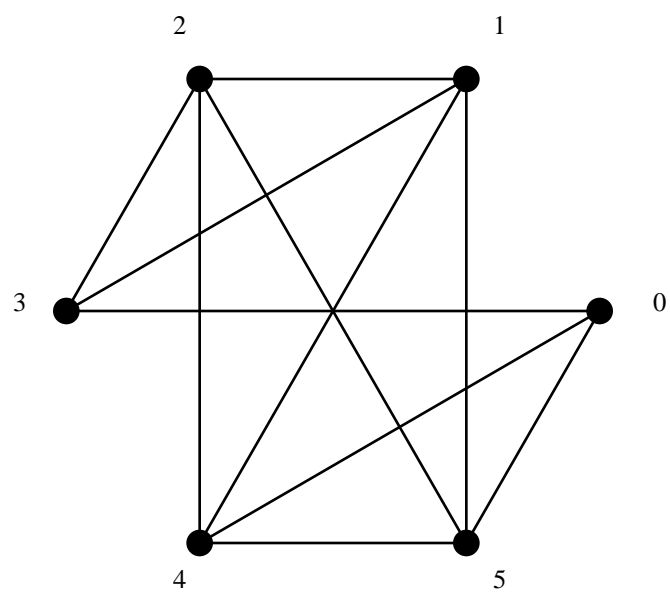

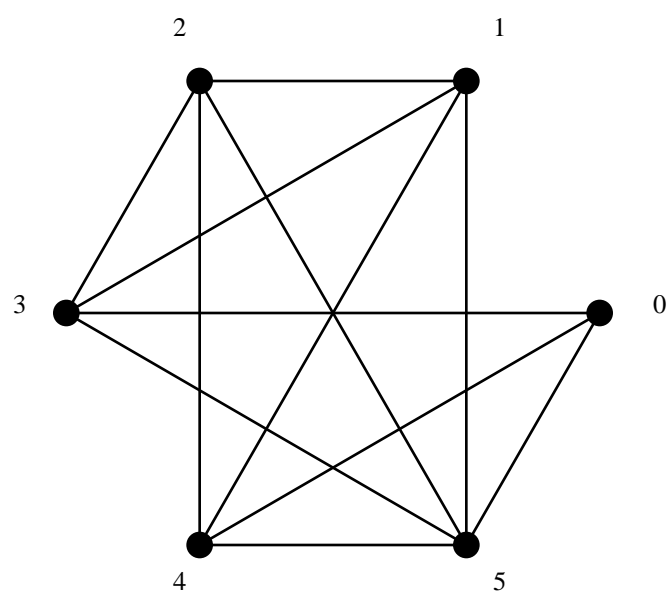

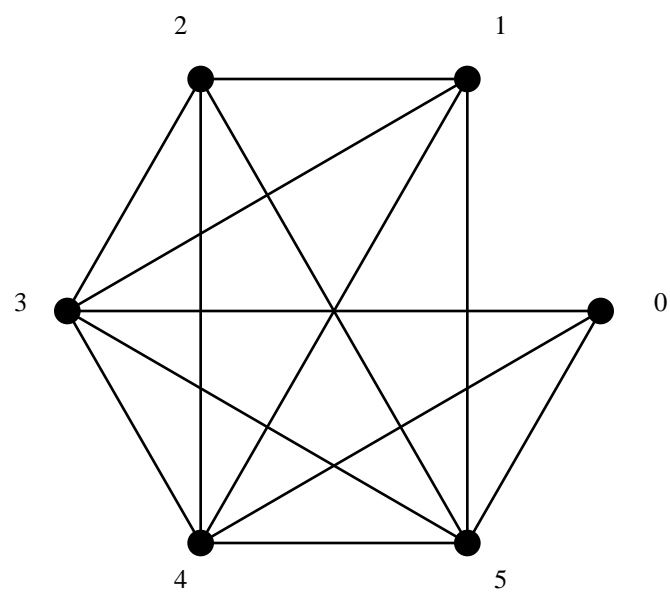

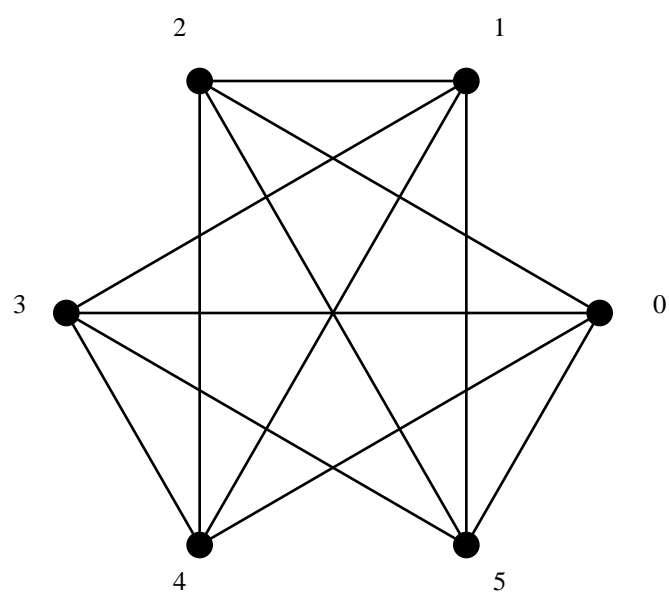

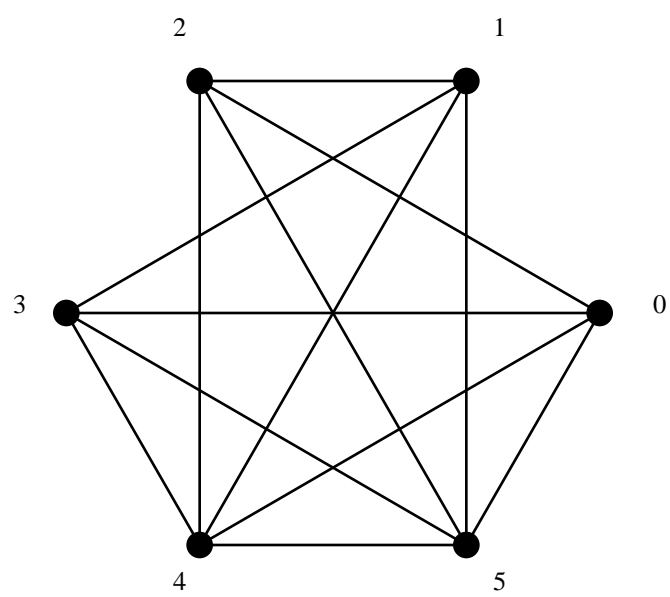

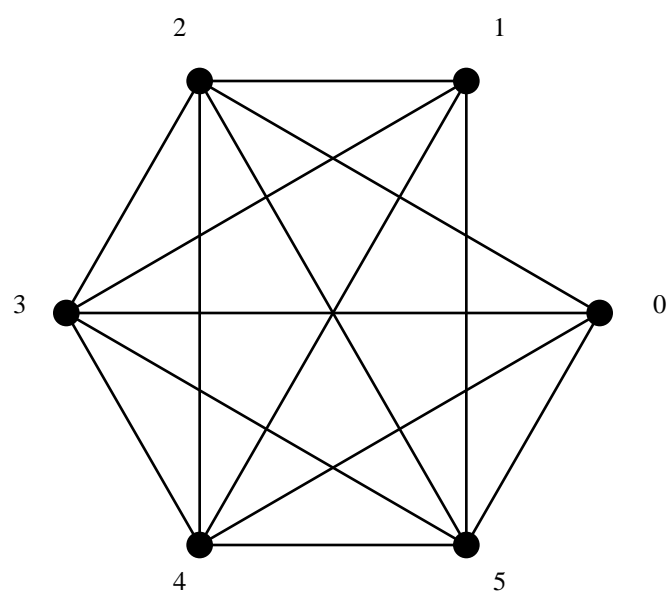

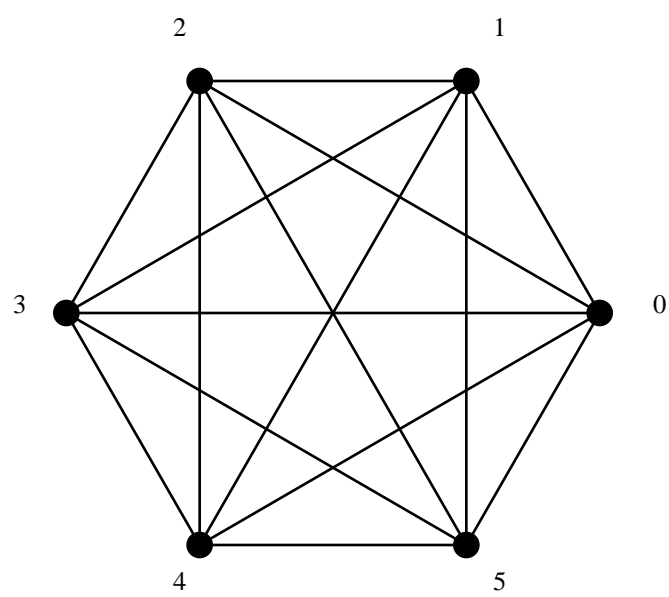

Supplement: S1 Fig — All graphlets of order 6 are shown in the newly introduced order. In the lower left corner of each page, the graphlet’s orbits are listed in their order. (PDF) [file pone.0147078.s004.pdf]
